# Nuclear Receptor Expression Defines a Set of Prognostic Biomarkers for Lung Cancer

**DOI:** 10.1371/journal.pmed.1000378

**Published:** 2010-12-14

**Authors:** Yangsik Jeong, Yang Xie, Guanghua Xiao, Carmen Behrens, Luc Girard, Ignacio I. Wistuba, John D. Minna, David J. Mangelsdorf

**Affiliations:** 1 Department of Pharmacology, University of Texas Southwestern Medical Center, Dallas, Texas, United States of America; 2 Howard Hughes Medical Institute, University of Texas Southwestern Medical Center, Dallas, Texas, United States of America; 3 Hamon Center for Therapeutic Oncology Research, University of Texas Southwestern Medical Center, Dallas, Texas, United States of America; 4 Simmons Cancer Center, University of Texas Southwestern Medical Center, Dallas, Texas, United States of America; 5 Department of Clinical Sciences, University of Texas Southwestern Medical Center, Dallas, Texas, United States of America; 6 Department of Internal Medicine, University of Texas Southwestern Medical Center, Dallas, Texas, United States of America; 7 Department of Thoracic/Head and Neck Medical Oncology, MD Anderson Cancer Center, University of Texas, Houston, Texas, United States of America; 8 Department of Pathology, MD Anderson Cancer Center, University of Texas, Houston, Texas, United States of America; Vanderbilt University, United States of America

## Abstract

**Background:**

The identification of prognostic tumor biomarkers that also would have potential as therapeutic targets, particularly in patients with early stage disease, has been a long sought-after goal in the management and treatment of lung cancer. The nuclear receptor (NR) superfamily, which is composed of 48 transcription factors that govern complex physiologic and pathophysiologic processes, could represent a unique subset of these biomarkers. In fact, many members of this family are the targets of already identified selective receptor modulators, providing a direct link between individual tumor NR quantitation and selection of therapy. The goal of this study, which begins this overall strategy, was to investigate the association between mRNA expression of the NR superfamily and the clinical outcome for patients with lung cancer, and to test whether a tumor NR gene signature provided useful information (over available clinical data) for patients with lung cancer.

**Methods and Findings:**

Using quantitative real-time PCR to study NR expression in 30 microdissected non-small-cell lung cancers (NSCLCs) and their pair-matched normal lung epithelium, we found great variability in NR expression among patients' tumor and non-involved lung epithelium, found a strong association between NR expression and clinical outcome, and identified an NR gene signature from both normal and tumor tissues that predicted patient survival time and disease recurrence. The NR signature derived from the initial 30 NSCLC samples was validated in two independent microarray datasets derived from 442 and 117 resected lung adenocarcinomas. The NR gene signature was also validated in 130 squamous cell carcinomas. The prognostic signature in tumors could be distilled to expression of two NRs, short heterodimer partner and progesterone receptor, as single gene predictors of NSCLC patient survival time, including for patients with stage I disease. Of equal interest, the studies of microdissected histologically normal epithelium and matched tumors identified expression in normal (but not tumor) epithelium of NGFIB3 and mineralocorticoid receptor as single gene predictors of good prognosis.

**Conclusions:**

NR expression is strongly associated with clinical outcomes for patients with lung cancer, and this expression profile provides a unique prognostic signature for lung cancer patient survival time, particularly for those with early stage disease. This study highlights the potential use of NRs as a rational set of therapeutically tractable genes as theragnostic biomarkers, and specifically identifies short heterodimer partner and progesterone receptor in tumors, and NGFIB3 and MR in non-neoplastic lung epithelium, for future detailed translational study in lung cancer.

*Please see later in the article for the Editors' Summary*

## Abstract 2

David Mangelsdorf and colleagues show that nuclear receptor expression is strongly associated with clinical outcomes of lung cancer patients, and this expression profile is a potential prognostic signature for lung cancer patient survival time, particularly for individuals with early stage disease.

## Editors' Summary

**Background:**

Lung cancer, the most common cause of cancer-related death, kills 1.3 million people annually. Most lung cancers are “non-small-cell lung cancers” (NSCLCs), and most are caused by smoking. Exposure to chemicals in smoke causes changes in the genes of the cells lining the lungs that allow the cells to grow uncontrollably and to move around the body. How NSCLC is treated and responds to treatment depends on its “stage.” Stage I tumors, which are small and confined to the lung, are removed surgically, although chemotherapy is also sometimes given. Stage II tumors have spread to nearby lymph nodes and are treated with surgery and chemotherapy, as are some stage III tumors. However, because cancer cells in stage III tumors can be present throughout the chest, surgery is not always possible. For such cases, and for stage IV NSCLC, where the tumor has spread around the body, patients are treated with chemotherapy alone. About 70% of patients with stage I and II NSCLC but only 2% of patients with stage IV NSCLC survive for five years after diagnosis; more than 50% of patients have stage IV NSCLC at diagnosis.

**Why Was This Study Done?:**

Patient responses to treatment vary considerably. Oncologists (doctors who treat cancer) would like to know which patients have a good prognosis (are likely to do well) to help them individualize their treatment. Consequently, the search is on for “prognostic tumor biomarkers,” molecules made by cancer cells that can be used to predict likely clinical outcomes. Such biomarkers, which may also be potential therapeutic targets, can be identified by analyzing the overall pattern of gene expression in a panel of tumors using a technique called microarray analysis and looking for associations between the expression of sets of genes and clinical outcomes. In this study, the researchers take a more directed approach to identifying prognostic biomarkers by investigating the association between the expression of the genes encoding nuclear receptors (NRs) and clinical outcome in patients with lung cancer. The NR superfamily contains 48 transcription factors (proteins that control the expression of other genes) that respond to several hormones and to diet-derived fats. NRs control many biological processes and are targets for several successful drugs, including some used to treat cancer.

**What Did the Researchers Do and Find?:**

The researchers analyzed the expression of NR mRNAs using “quantitative real-time PCR” in 30 microdissected NSCLCs and in matched normal lung tissue samples (mRNA is the blueprint for protein production). They then used an approach called standard classification and regression tree analysis to build a prognostic model for NSCLC based on the expression data. This model predicted both survival time and disease recurrence among the patients from whom the tumors had been taken. The researchers validated their prognostic model in two large independent lung adenocarcinoma microarray datasets and in a squamous cell carcinoma dataset (adenocarcinomas and squamous cell carcinomas are two major NSCLC subtypes). Finally, they explored the roles of specific NRs in the prediction model. This analysis revealed that the ability of the NR signature in tumors to predict outcomes was mainly due to the expression of two NRs—the short heterodimer partner (SHP) and the progesterone receptor (PR). Expression of either gene could be used as a single gene predictor of the survival time of patients, including those with stage I disease. Similarly, the expression of either nerve growth factor induced gene B3 (NGFIB3) or mineralocorticoid receptor (MR) in normal tissue was a single gene predictor of a good prognosis.

**What Do These Findings Mean?:**

These findings indicate that the expression of NR mRNA is strongly associated with clinical outcomes in patients with NSCLC. Furthermore, they identify a prognostic NR expression signature that provides information on the survival time of patients, including those with early stage disease. The signature needs to be confirmed in more patients before it can be used clinically, and researchers would like to establish whether changes in mRNA expression are reflected in changes in protein expression if NRs are to be targeted therapeutically. Nevertheless, these findings highlight the potential use of NRs as prognostic tumor biomarkers. Furthermore, they identify SHP and PR in tumors and two NRs in normal lung tissue as molecules that might provide new targets for the treatment of lung cancer and new insights into the early diagnosis, pathogenesis, and chemoprevention of lung cancer.

**Additional Information:**

Please access these Web sites via the online version of this summary at http://dx.doi.org/10.1371/journal.pmed.1000378.

- The Nuclear Receptor Signaling Atlas (NURSA) is consortium of scientists sponsored by the US National Institutes of Health that provides scientific reagents, datasets, and educational material on nuclear receptors and their co-regulators to the scientific community through a Web-based portal
- The Cancer Prevention and Research Institute of Texas (CPRIT) provides information and resources to anyone interested in the prevention and treatment of lung and other cancers
- The US National Cancer Institute provides detailed information for patients and professionals about all aspects of lung cancer, including information on non-small-cell carcinoma and on tumor markers (in English and Spanish)
- Cancer Research UK also provides information about lung cancer and information on how cancer starts
- MedlinePlus has links to other resources about lung cancer (in English and Spanish)
- Wikipedia has a page on nuclear receptors (note that Wikipedia is a free online encyclopedia that anyone can edit; available in several languages)

## Introduction

The prevalence of lung cancer as the primary cause of cancer death in the United States has led to renewed efforts to obtain biomarker signatures that provide either prognostic or predictive information to guide therapy for individual patients (i.e., “personalized medicine”) [1]–[3]. Multiple genome-wide expression studies have demonstrated the usefulness of this approach for lung cancer prognosis [4]–[6]. However, in general, each of these studies has identified different sets of genes, even when the various studies are used to cross-validate one another, and in the majority of these studies the individual genes identified have had little impact for understanding tumor pathogenesis or as therapeutic targets. Thus, identification of prognostic biomarkers that also provide hypotheses for mechanism-based studies of carcinogenesis and offer new therapeutic targets (sometimes referred to as “theragnostics”) would be of significant benefit.

Nuclear receptors (NRs) are a large family of ligand-dependent transcription factors that respond to a number of hormonal and diet-derived lipids, including endocrine steroids, fat-soluble vitamins, fatty acids, and cholesterol metabolites [7]. NRs are also among the most successful targets of drugs approved to treat many diseases, including cancer [8]. Previously, we have shown that NR expression profiling can be used to reveal the mechanistic basis of the hierarchical transcriptional networks that govern a number of physiological processes, including development, differentiation, reproduction, circadian rhythm, and metabolism [9]–[13]. In the present study, we wished to investigate the potential role of the 48 members of the NR superfamily as “theragnostic” indicators in lung cancer. The strategy of examining expression of NRs, which are known therapeutic targets with defined mechanisms of action, differs from previous, open-ended genome-wide microarray studies. Thus, our goal was to determine whether one can use NR expression signatures as clinical tools for prognosis for patients with lung cancer, which also might lead to NR-selective therapies targeted at hormonal manipulation of lung cancer. As an additional aspect of this study, we wished to provide open access to our data by including a Sweave document [14]–[16] that contains a literate programming package to permit the full reproduction of our analysis.

## Methods

### Sample Collection

#### Samples from MD Anderson Cancer Center

**Figure 1:**
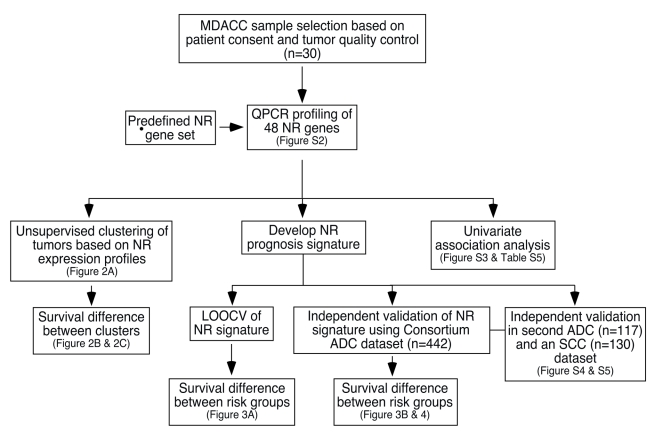
Schematic of the study design for development and validation of the NR prognostic gene signature. The flow chart details the design and implementation of this study. ADC, adenocarcinoma; SCC, squamous cell carcinoma.

All tissue samples were obtained by surgical resection from patients who had provided written informed consent under approval of the institutional review boards at MD Anderson Cancer Center (MDACC). Tissues were stored at −80°C after being snap frozen in liquid nitrogen. Serial sectioning of each sample was used to histologically evaluate tumor and normal tissue for subsequent microdissection [17]. Thirty primary tumor and corresponding normal tissues (including 22 adenocarcinomas and eight squamous cell carcinomas) were selected randomly from 379 similar samples in the MDACC lung tumor collection based on stringent, predefined quality control procedures before any data analysis (see Figure 1 for overall study design). Detailed sample selection procedures are described in Text S1. Among the 30 patient samples, 17 were diagnosed with stage I (the earliest stage) disease, four with stage II disease, five with stage III disease, and four with stage IV disease. A comparative analysis of the 30 samples demonstrated that they represent an objective sampling of the MDACC lung cancer tumor collection (Figure S1; Table S1). RNAs were isolated from each sample using the Qiagen RNeasy Mini Kit (Qiagen Sciences).

#### Samples from the National Cancer Institute Director's Consortium and other datasets

Validation of the prediction model from the MDACC samples was performed using an independent dataset from a recently published National Cancer Institute (NCI) Director's Consortium study of lung cancer involving 442 resected non-small-cell lung cancers (NSCLCs) [6]. This Consortium dataset represents one of the largest microarray datasets of NSCLC samples that has been collected and studied using a common protocol. From this study, the Affymetrix U133A microarray data for the 48 NR gene expression signatures were excerpted and used as shown in Figure 1 to validate the prognostic value of NR expression. In a similar manner, the MDACC signature was validated in the NR expression microarray data taken from a second set of 117 adenocarcinomas [18]. Finally, the NR signature from the Consortium adenocarcinoma dataset was cross-validated using the 48-gene NR expression profile excerpted from the microarray of 130 squamous cell carcinomas [19].

### Reverse Transcription and Quantitative PCR Assay

All cDNAs were prepared for quantitative PCR (QPCR) (TaqMan method) as previously described [10]. Briefly, 2 µg of total RNA was DNAse-treated with 2 U of DNAse I in final volume of 20 µl containing 4.2 µM MgCl_2_. The reverse transcription reaction was performed in 100 µl final volume, followed by addition of 100 µl of DEPC-H_2_O. Human universal cDNAs for broadly expressed NRs or tissue-specific cDNAs for restricted-expression NRs was used to construct a standard curve of the following concentrations: 0 (i.e., no template control), 0.008, 0.04, 0.2, 1, 5, and 25 ng of 18S RNA; and 0 (i.e., no template control), 0.016, 0.08, 0.4, 2.0, 10, and 50 ng of each NR RNA. These quantities were based on the RNA concentration used for the reverse transcription reaction. A negative reverse transcription sample and a control for genomic DNA contamination were included for both 18S and NR. Per sample, 10 ng of cDNA was assayed in triplicate wells of a 384-well plate. The final forward and reverse primer concentrations used were 75 nM for 18S rRNA and 300 nM for all NRs. For this study the 48 NRs plus the two common splice variants for PPARγ (i.e., PPARγ2) and PPARδ (i.e., PPARδ2) were included in the analysis of all samples from the MDACC patient set. Primer sequences have been reported elsewhere (http://www.nursa.org/datasets.cfm?doi=10.1621/datasets.05005).

### QPCR Data Analysis

Data were imported into Microsoft Excel and evaluated for PCR efficiency (*e*), *e* = 10^−1/slope^, where the slope was obtained from the standard curve calculated by the sequence detection system software of the ABI7900 instrument (Applied Biosystems) for the endogenous 18S reference and target NR. Relative mRNA amounts were calculated by quantity  =  *e* ^–Ct^, where Ct is cycle time. The calculated quantities were averaged (avg), and the standard deviations (stdev) and coefficients of variation (CV  =  stdev/avg) were determined for the 18S and NR of each sample. Data points that showed CV >17% were considered outliers and removed. A 17% CV cutoff correlates with the maximum allowable standard deviation that can distinguish a 2-fold change with 99% confidence when samples are assayed in triplicate wells for both the endogenous reference and the gene of interest. Note that only one point per replicate may be removed. Normalized values for expression of each NR were calculated using normalized value  =  NR quantity avg/18S quantity avg. The standard deviation of the normalized value was calculated as (normalized value) × [(CV of reference)^2^ + (CV of gene of interest)^2^]^½^. Normalized values are represented as a bar graph. QPCR data analysis procedures have been described previously [10],[12]. The entire QPCR dataset of NR expression in normal and tumor samples from the 30-patient cohort is available in Figure S2 and online at http://www.nursa.org/datasets.cfm?doi=10.1621/datasets.05010.

### Microarray Data Preprocessing

Consortium microarray raw data [3],[6] were downloaded from the NCI's caArray database and preprocessed by robust multichip average background correction and quantile–quantile normalization [20]. All gene expression values were log-transformed (on a base 2 scale). Average values were used for the different probe sets corresponding to the same gene.

### Unsupervised Clustering Analysis

The hierarchical clustering algorithm [21] was used to group NR expression versus the 30-MDACC-patient cohort based on the QPCR expression profile. Gene expression values were log-transformed (on a base 2 scale) in a manner similar to the transformation of the microarray data. Euclidian distance and average link were used in the hierarchical clustering algorithm.

### Supervised Classification Analysis

Supervised classification was performed using Recursive Partitioning and Regression Trees (RPART) [22]–[25], and was implemented using R software version 2.10.0. A detailed description of the implementation is provided in Text S1.

### Survival Analysis

Overall survival time was calculated from the date of surgery until death or the last follow-up contact. Recurrence-free survival time was defined as the time interval between the date of surgery and the date of disease recurrence or death from any cause, whichever came first, or date of last follow-up evaluation. Survival curves were estimated using the product-limit method of Kaplan-Meier [26] and were compared using the log-rank test. Cox proportional-hazards analysis [27] was also performed, with survival time as the dependent variable.

### Sweave Report

A Sweave document is provided () to permit others to reproduce any or all parts of our statistical analyses. Sweave is a literate programming R package that combines the source code (in R) and documentation (in LaTeX) in one file and thereby permits reproduction of published high-throughput data analysis [14]–[16].

## Results

### Association of NR Gene Expression with Survival Time

The overall schema for the design and data analysis of this study is shown in Figure 1. The expression of all 48 members (plus two splice isoforms) of the NR superfamily was investigated first in a 30-patient sample set to develop a prognostic signature. Detailed clinical data on the 30-patient cohort are given in Tables S1–S3. The QPCR datasets of NR expression are shown and summarized in Figure S2 and Table S4. Raw datasets are available at http://www.nursa.org/datasets.cfm?doi  = 10.1621/datasets.05010.

#### Univariate analysis

We first explored whether the mRNA expression of NR genes was associated with survival outcomes for patients with lung cancer. Table S5 shows hazard ratios (HRs) and corresponding *p*-values for the association between each NR gene and survival outcome from the univariate Cox regression models. The mRNA expression of 37 NR genes was significantly associated with survival outcomes (*p*<0.05). Figure S3 shows the cumulative distribution function (CDF) of the *p*-values representing the associations between individual NR gene expression and survival time from univariate Cox models. These data show that the NR gene superfamily as a whole has statistically significant association with survival outcome (*p*<0.00001) based on a Kolmogorov-Smirnov test of the difference between the *p*-value distributions of NR sets and random gene sets.

#### Unsupervised clustering analysis

**Figure 2:**
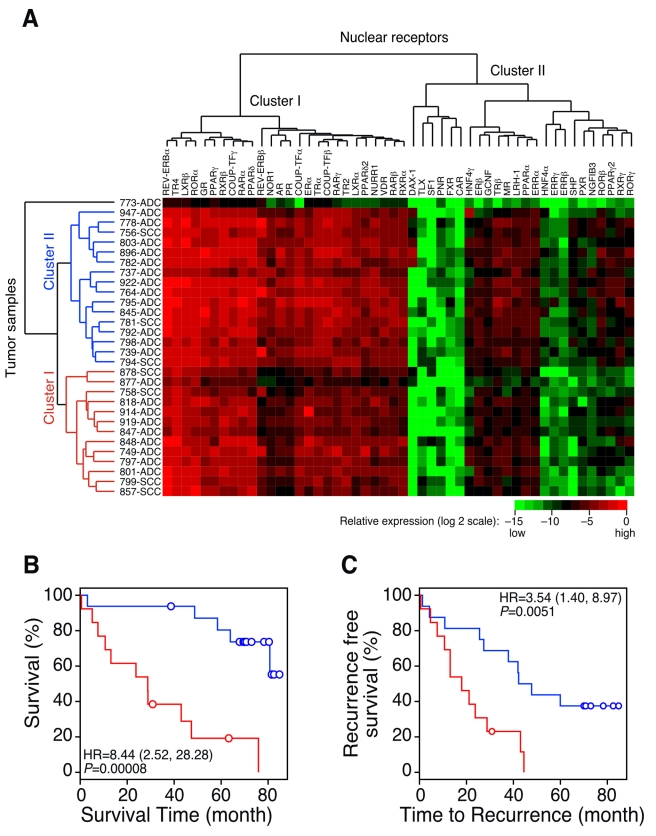
QPCR analysis of the NR gene expression signature in patients with lung cancer. (A) Unsupervised cluster analysis of the 30-patient MDACC lung cancer cohort using the QPCR profile of the NR superfamily. Horizontal and vertical axes represent NR and lung cancer patient clusters, respectively. (B and C) Kaplan-Meier plots showing the association of the NR gene signature with overall patient survival (B) and disease recurrence (C). *p*-values were obtained using the log-rank test. Red represents sample Cluster I and blue represents Cluster II, defined by an unsupervised clustering algorithm using the NR gene profiling data in (A). Circles indicate censored samples. ADC, adenocarcinoma; SCC, squamous cell carcinoma.

Unsupervised cluster analysis (with euclidian distance and average link) of NR expression in lung tumors revealed two distinct clusters of tissue samples (Figure 2A). Note that one tissue sample (773-ADC) did not fall into either cluster and was treated as an outlier for the clustering analysis. The two major branches of the dendrogram (cluster 1 and cluster 2) were associated with both overall survival rates (*p* = 0.0079) and disease recurrence rates (*p* = 0.0927), but no other clinical features (Table 1). Indeed, Kaplan-Meier plots for survival time and disease recurrence showed that cluster 1 and cluster 2 segregated patients into those with poor and good prognostic outcomes, respectively (*p* = 0.00008 for survival time; *p* = 0.0051 for disease recurrence) (Figure 2B and 2C). These findings suggest that the gene expression of the NR family is strongly associated with clinical outcomes for patients with lung cancer.

**Table 1:** Patient demographics summarized by unsupervised cluster analysis of lung tumors.

Characteristic	Subcategory	Cluster 1	Cluster 2	*p*-value^a^
**Sample size**		13	16	
**Age (mean ± standard error)**		62.6±2.4	63±2.1	0.902
**Gender (% female)**		38%	56%	0.4621
**Race (% non-white)**		0%	13%	0.488
**Histology (ADC/SCC)**		8/5	13/3	0.4058
**Cancer stage**	Stage I	62%	56%	0.8324
	Stage II	8%	19%	
	Stage III	15%	19%	
	Stage IV	15%	6%	
**Death rate**		85%	31%	0.0079
**Disease recurrence rate**		92%	63%	0.0927
**Smoking status**	Non-smoker	15%	13%	1.0000
	Current/former smoker	11/3	8/3	1.00
	Packs per year	70.7±35.9	61.2±26.2	0.45
**Adjuvant therapy**		15%	6%	0.5731

a Indicates *p*-values by *t* test for age and by Fisher's exact test for the other variables comparing cluster 1 and 2.

ADC, adenocarcinoma; SCC, squamous cell carcinoma.

### Development and Validation of a Prognostic NR Signature for Lung Cancer

**Figure 3:**
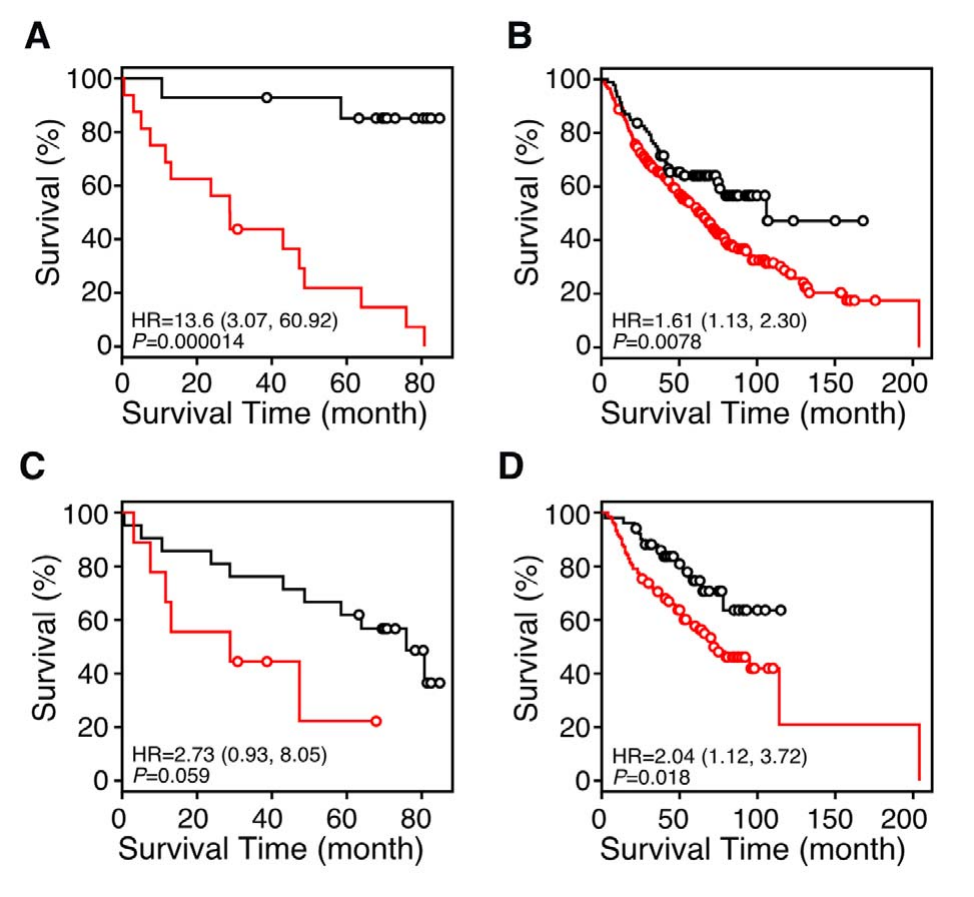
Kaplan-Meier plots showing the predictive power of the NR gene signature in datasets from the NCI Director's Consortium. (A) LOOCV of the recursive partitioning tree model (RPART) for the 30-sample MDACC QPCR dataset using all 48 NRs. The HR for the predicted high-risk versus the predicted low-risk signatures was 13.6; 95% CI, 3.07–60.92; *p* = 0.000014. (B and C) Independent validation of the 48-NR gene expression signature between the MDACC cohort and the Consortium cohort. The MDACC cohort training set (*n* = 30) was tested in the Consortium cohort (*n* = 442) (B), and vice versa (C). (D) Independent validation of the NR gene signature in the 442-sample multi-institute Consortium using RPART analysis. The microarray datasets were divided into two groups, one for the training cohort (*n* = 256) and the other for the testing cohort (*n* = 186). *p*-values were obtained by the log-rank test. Red and black lines represent predicted high- and low-risk groups, respectively. Circles indicate censored samples. Note that SHP expression is the single co-variable (or predictor) in the classification model that describes the survival time differences shown in (B), demonstrating that SHP is a single gene predictor that represents the entire 48-NR gene profile.

Standard classification and regression tree analysis [22]–[25] was used to build a prognostic model based on NR gene expression in the MDACC NSCLC 30-patient cohort. We first evaluated the performance of the prognosis model using the leave-one-out cross-validation (LOOCV) method. The HR, i.e., risk of death, for the predicted high-risk versus the predicted low-risk signatures using tumor samples was 13.6; 95% confidence interval (CI), 3.07–60.92; *p* = 0.000014 (Figure 3A). The relatively large CI for the HR in the MDACC cohort is due to the small sample size in this dataset. These results prompted us to validate the NR signature in a larger dataset.

Because the majority of gene expression data now available from lung cancer samples comes from microarray expression studies, we investigated whether the NR expression profile could be validated within a completely independent dataset taken from the NCI Director's Consortium study of lung cancer involving 442 resected NSCLCs [6]. We first validated the 30-sample QPCR dataset on the 442-sample microarray dataset, and then we developed an NR signature from the microarray dataset and validated it on the QPCR data. Both directions of training and testing provided statistically significant predictive power for patient survival time (Figure 3B and 3C).

As a further validation test of the NR gene signature, we divided the 442-sample microarray data into training and testing sets, and analyzed the data using the predictive RPART model. We used the same training and testing strategy as in the genome-wide analyses of these data [6]. The training set (*n* = 256) included samples from the University of Michigan Cancer Center (*n* = 177) and the Moffitt Cancer Center (*n* = 79), and the testing set (*n* = 186) included samples from the Memorial Sloan-Kettering Cancer Center (*n* = 104) and the Dana-Farber Cancer Institute (*n* = 82). Using the NR expression profile from training data to build a predictive model yielded a HR of 2.04 (95% CI, 1.12–3.72; *p* = 0.018) for the predicted high-risk versus the predicted low-risk signature in testing data (Figure 3D). The higher HR value for the QPCR dataset likely reflects the greater dynamic range and quantitative nature of the QPCR assay, and the greater homogeneity of the microdissected samples.

As yet further confirmation of the prognostic NR gene signature, the NR prognosis signature developed using the MDACC dataset was validated in another dataset containing 117 adenocarcinoma samples [18]. Again, the results show that patients in the predicted low-risk group have significantly longer survival times than those in the predicted high-risk group (*p* = 0.0053; Figure S4). The results from this second independent dataset further confirmed the robustness of the NR prognosis signature.

Finally, we also examined whether the NR prognosis signature might be applicable to squamous cell carcinomas. To that end, the NR prognosis signature developed using the Consortium lung adenocarcinoma dataset was validated in a previously published dataset from Raponi et al. containing 130 lung squamous cell carcinomas [19]. Patients in the predicted low-risk group had significantly longer survival times than those in the predicted high-risk group (*p* = 0.018; Figure S5A). We then used lung squamous cell carcinomas (from the Raponi et al. dataset) to develop a NR prognosis signature and validated it in the lung adenocarcinomas (Consortium dataset). Again, the results showed that NR expression has significant predictive power (Figure S5B). Based on these results, we conclude that the NR prognosis signature may not be limited only to adenocarcinomas.

To underscore the significance of the 48-NR gene signature, we used 1,000 random gene sets, each comprising 48 transcripts, to build prediction models in the Consortium training set and then tested them in the Consortium testing set. The distribution of the association between the predicted risk groups and the survival outcomes (*p*-values) is shown in Figure S6; only 28 of the 1,000 random sets reached the significance level of the NR signature (*p = *0.018), thereby demonstrating that the NR signature is highly selective and nonrandom. Taken together, these results strongly support the utility of the NR gene signature as a prognostic marker, even when applied and cross-validated independently by two different gene expression platforms (i.e., QPCR and microarray) and in multiple independent lung cancer datasets.

### NR Signature Prediction Is Independent of Clinical Variables

We examined whether the association between the NR signature and the survival outcome was independent of clinical variables by using a multivariate Cox proportional-hazards analysis that included NR risk score, gender, age at diagnosis, use of adjuvant chemotherapy, use of adjuvant radiation therapy, and stage as the co-variables. We analyzed the Consortium testing dataset, which included samples from the Memorial Sloan-Kettering Cancer Center and the Dana-Farber Cancer Institute. The NR risk scores used in this analysis were derived from the prediction model built from the Consortium training dataset (from the University of Michigan Cancer Center and the Moffitt Cancer Center). Surprisingly, this multivariate analysis revealed that the association between NR risk scores and survival time was independent of other clinical variables (HR = 1.98; 95% CI, 1.04–3.77; *p* = 0.037) (Table 2). These data reveal that a significant association exists between a patient's NR profile and survival time when adjusted for other clinical variables. As expected, the correlation between tumor stage and patient survival time was also highly significant, confirming this clinical feature as a well-recognized prognostic marker used in the clinic. It is interesting to note that gender also was significantly correlated with patient survival time in our analysis (males had a higher risk of death than females).

**Table 2:** Death HRs from multivariate Cox regression analysis from two independent datasets.

Variable	MSK and CAN/DF Dataset^a^	
	HR (95% CI)	*p*-value
Gender	1.88 (1.11, 3.17)	0.019
Age at diagnosis	1.02 (0.99, 1.05)	0.22
Adjuvant chemotherapy	2.02 (1.14, 3.59)	0.016
Adjuvant radiation therapy	1.48 (0.81, 2.71)	0.210
Stage	2.76 (1.56, 4.88)	0.00046
NR signature^b^	1.98 (1.04, 3.77)	0.037

a Memorial Sloan-Kettering Cancer Center (MSK) and Dana-Farber Cancer Institute (CAN/DF).

b The NR signature for the Memorial Sloan-Kettering Cancer Center and Dana-Farber Cancer Institute dataset (*n* = 186) was derived from the prediction model built from the University of Michigan Cancer Center and Moffitt Cancer Center training dataset.

Next, we investigated whether combining clinical variables and NR gene expression could improve upon the prognosis based on clinical variables alone. The Consortium microarray dataset was divided into two groups, one for the training cohort and the other for the testing cohort. From these samples, the two principal components of NR gene expression and clinical variables (including gender, age, stage, and adjuvant therapy) were used to build a prediction model using the classification tree approach (Figure S7). This analysis shows that the predicted risk groups from the prognosis model based on the combination of clinical variables and NR expression have a stronger association with survival time (HR = 3.21, 95% CI, 2.01–5.12; *p* = 2.43×10^−7^) than models using the NR signature alone (HR = 1.98, 95% CI, 1.71–4.30; *p* = 0.037) or the clinical variables alone (HR = 2.71, *p* = 1.02×10^−5^).

### Refinement of the NR Signature into Single Gene Predictors

**Figure 4:**
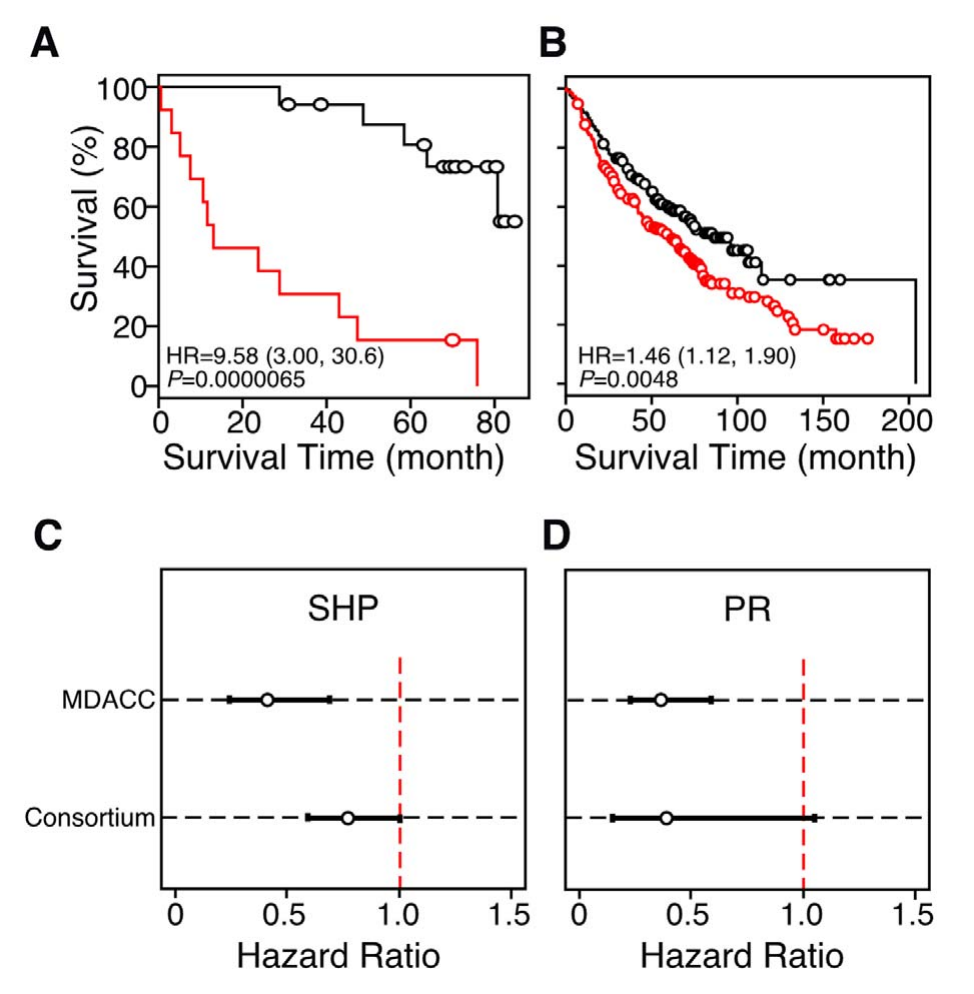
Identification of single NR gene biomarkers for lung cancer prognosis. (A and B) Kaplan-Meier survival plots using PR in the single gene prediction model. The MDACC cohort was tested using LOOCV (A), or it was used as a training set and independently tested in the multi-site Consortium cohort (B). For this analysis mRNA expression values for SHP were removed from the dataset in order to test the effect of other NR genes as biomarkers. In this case, PR expression is the single co-variable (or predictor) in the classification model that describes the survival time differences shown in (B), demonstrating that PR is a single gene predictor that represents the NR gene profile when SHP expression is excluded. *p*-values were obtained using the log-rank test. Red and black lines represent high- and low-risk groups, respectively. Circles indicate censored samples. (C and D) HRs from univariate Cox regression models for SHP and PR expression, respectively, in the MDACC and multi-site Consortium datasets.

We next explored the roles of specific NRs in the prediction models. To address this question, we further re-interrogated the classification tree model to see whether the prediction model outcomes shown in Figures 3A and 3B were due to the dominant effects of any specific NRs (see Text S1 for details). When using all 48 NR genes as input for the classification tree model, short heterodimer partner (SHP) expression was identified as the only co-variable left in the final RPART prediction model built from the 30-patient MDACC dataset. In other words, the prognosis performance of the 48-NR gene signature (shown in Figure 3A and 3B) is the same as that using SHP expression alone to build the models. Next, we removed SHP from the dataset, reanalyzed the prediction model, and found the model still had remarkable prognostic ability in the 30-patient LOOCV dataset (HR = 9.58; 95% CI, 3.00–30.6; *p* = 0.0000065) (Figure 4A). Furthermore, the classification tree structure revealed that when the prediction model excluded SHP, progesterone receptor (PR) was the single gene signature used. The detailed classification tree structures are shown in the Sweave document (Text S2) and plotted in Figure S8. The single gene PR signature was further validated in the testing cohort of the Consortium dataset (HR = 1.46; 95% CI, 1.12–1.90; *p* = 0.0048) (Figure 4B). The protective effect based on SHP and PR expression was further strengthened by univariate Cox regression modeling, which consistently showed that expression of both NRs correlated with significantly lower HRs in the microarray dataset (Figure 4C and 4D). Thus, SHP and PR represent single gene markers.

### SHP Expression Predicts Survival Time in Patients with Early Stage Lung Cancer

**Figure 5:**
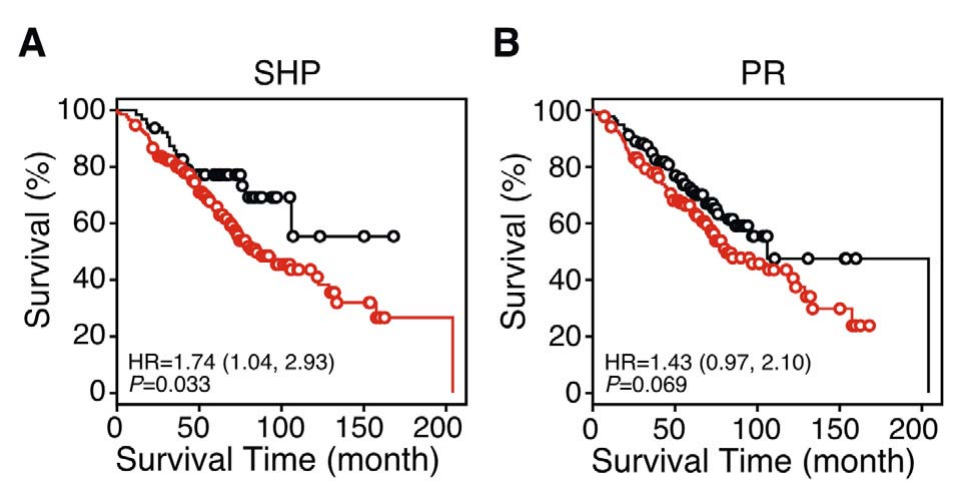
Kaplan-Meier survival plots showing single NR gene predictors in patients with stage I lung cancer. Predictive models for SHP (A) and PR (B) were trained in the MDACC samples and tested in the patients with stage I lung cancer in the Consortium cohort. *p*-values were obtained by the log-rank test. Red and black lines represent predicted high- and the low-risk groups, respectively. Circles indicate censored samples.

Since the prognosis for patients with early stage (i.e., stage I) lung cancer has substantial clinical impact on guiding therapeutic strategy, we tested whether expression of specific NRs also has predictive power to refine the prognosis of patients with stage I lung cancer. We found that the predicted risk groups using SHP as a single gene classifier were significantly associated with survival outcome for patients with stage I lung cancer in the Consortium samples (Figure 5A; *p* = 0.033), whereas the PR signature was marginally predictive (Figure 5B; *p* = 0.069).

### NR Expression in Normal Tissue Predicts Survival and Disease Recurrence

We also examined the potential prognostic value of NR expression in histologically normal lung tissue obtained from areas adjacent to the tumors of the MDACC cohort used in the above studies. When the normal tissue expression data were analyzed using the classification tree model and validated by LOOCV, the NR signature provided statistically significant predictors of both disease recurrence (HR = 4.61, 95% CI, 1.74–12.30; *p* = 0.00099) and overall patient survival time (HR = 2.22, 95% CI, 0.85–5.81; *p* = 0.094) (Figure S9). In contrast to the identification of SHP and PR in the tumor samples as key predictors, the normal lung epithelium profile classification tree structures revealed two other NRs, NGFIB3 (nerve growth factor induced gene B3, also known as NR4A1) and mineralocorticoid receptor (MR, also known as NR3C2) to be single gene predictors for survival and disease recurrence. Although the prediction models for normal tissue require further validation in an independent dataset, this analysis suggests higher expression of NGFIB3 and MR is associated with a good prognosis.

To further understand the relationship between NR expression levels in the tumor versus adjacent normal tissue, we performed a pair-wise correlation of NR gene expression in tumor and adjacent normal lung from each patient's paired tissue set. For this analysis we used both the MDACC dataset and a recently published microarray dataset from Landi et al. comparing gene expression in 135 NSCLC adenocarcinomas and adjacent normal tissue [28]. In both datasets we found that the NR expression in tumor samples correlated significantly with NR expression in adjacent normal samples (Pearson correlations were 0.87, *p*<0.001, and 0.92, *p*<0.001, respectively) (Figure S10). This analysis suggests that, in general, there is a tight correlation between tumor and normal tissue NR expression within the same patient. We next asked which of the 48 NRs might be differentially expressed between tumor and adjacent normal samples using paired *t* tests. Again, the *t* tests derived from the two datasets were consistent (Pearson correlation  = 0.7, *p*<0.001). Furthermore, five of the top ten NR genes that were shown to differ significantly between tumor and normal tissue were common to both datasets (i.e., AR, MR, NGFIB3, PPARγ, and RXRγ). Of note was the finding that MR and NGFIB3 were among these receptors, further emphasizing their prognostic potential.

## Discussion

In this study, we investigated the expression of NRs in lung cancer and found that specifically targeting this superfamily of ligand-dependent transcription factors provided a novel prognostic biomarker. Several recent studies using genome-wide microarray experiments have proposed various sets of genetic signatures for lung cancer prognosis [4]–[6],[29]–[31]. Interestingly, the gene signatures from these studies have shared little if any overlap with one another [4],[5],[29]. In addition, because of the open-ended nature of genome-wide analyses, the signatures have provided little insight into the pathogenesis or pathophysiology of lung cancer. Moreover, these studies have yet to identify new therapeutic targets. In contrast, NRs represent a well-studied class of proteins that (1) govern complex cellular programs such as differentiation, inflammation, and metabolism [9]–[13], (2) are known transcriptional drivers of oncogenesis, and (3) are themselves the targets of validated drugs for many diseases including cancer [32]–[35]. This superfamily also includes a number of orphan receptors, many of which are currently being evaluated as potential new therapeutic targets for a number of diseases [8]. Thus, our study suggests that specifically targeting NRs may provide an alternative and clinically relevant new strategy for profiling lung tumors.

The immediate findings from this work also may have a number of important and practical implications for the use of the NR gene signature in a clinical setting. First, we demonstrated that the NR superfamily gene expression signature is an excellent predictor of both patient survival and recurrence of lung cancer that is comparable to predictors from other studies. As an example, Shedden et al. [6] compared six previously published gene signatures and showed that the HRs from these studies ranged from 0.93 to 2.30 (*p*-values ranged from *p*<0.001 to *p = *0.78). In comparison, the HR for our NR signature obtained in the Consortium dataset [6] was 2.04 (*p* = 0.018) (Figure 3D).

The prognostic potential of NRs was validated in independent datasets, and further analysis identified tumor expression of SHP (discussed below) and PR as robust single gene predictors. The demonstration of PR as a predictive marker is in line with a previous retrospective study where PR was shown to be associated with survival time in patients with lung adenocarcinoma [36]. Expression of PR, together with estrogen receptor α (ERα), is now well established as a clinical guide to both prognostic anticipation and therapeutic intervention for breast cancer. Indeed, in thinking about the next step in our studies, the finding that certain lung cancers express specific, known therapeutic NR targets (e.g., PR, ERα, ERβ, AR, RARs, and PPARs) brings up the possibility of treating patients whose tumors express these receptors with drugs (agonists or antagonists) that target the receptors. Although it remains to be established whether NR mRNA levels will correlate with NR protein expression, several recent studies support the concept that selective receptor modulators may be effective therapeutically. For example, a number of studies have suggested anti-estrogen therapy as a lung cancer therapeutic [37]–[40], and in a mouse lung cancer study, the use of a PPARγ agonist had a synergistic effect in reducing tumor burden when used with cis-platinum [41]. Interestingly, treatment with progesterone has been shown to inhibit lung tumor xenograft growth in a preclinical study [36], whereas, in contrast, the Women's Health Initiative study reported increased death from lung cancer in postmenopausal women treated with both estrogen and progestins [42]. At present it is not clear whether this latter finding was due to the presence of estrogen or progestin, highlighting the need for further investigation. Nevertheless, a reasonable assumption based on the present study is that predicting responses to drugs like anti-estrogens might be accomplished by screening patients for NR expression using the methodology highlighted in this study. Evaluation of the QPCR profiles from our study revealed a high degree of patient-to-patient variability in NR expression (Figure S2), and this observation provides a strong rationale for using this approach to guide individualized treatment in the future. Similarly, our data suggest that NR profiling of individual tumors provides a clinical paradigm for identifying potential responders to NR drugs.

A second notable finding was that the NR signature could be used to refine the prognosis for patients with early stage lung cancer. Although the clinical characteristics from the MDACC dataset and the Consortium dataset are not homogeneous and the sample size is not big enough to validate the prognosis signatures for stages IA, IB, and II separately, the positive prognosis results across the two datasets indicate that the signatures may be applied across heterogeneous populations of patients. In interrogating this signature further, it was of considerable interest to find that the orphan nuclear receptor SHP is a singe gene prognostic lung cancer biomarker of early stage disease. SHP has been extensively studied for its role in liver lipid metabolism [43],[44] and as transcriptional repressor of other NRs [45]. Intriguingly, a recent report found that SHP expression was negatively associated with liver tumorigenesis in a mouse model [46]. These findings prompt further exploration into whether there is a connection between the known physiological role of SHP and lung tumorigenesis, or whether SHP has a unique pathophysiologic function in the disease pathogenesis. To that end, we note that FXR agonists, a PPARγ agonist (rosiglitazone), agents that inhibit HNF-1α action, and a number of orphan drugs are all inducers of SHP expression [47]. These compounds might be tested in preclinical models to see whether they can induce SHP expression and inhibit lung tumorigenesis or malignant behavior. Also germline mutations in SHP or polymorphisms in FXR that regulate the level of SHP expression could play a role in SHP function in lung cancer pathogenesis or behavior. Together, these findings suggest that SHP expression may not only be a prognostic biomarker, but its presence in tumors may influence the expression of other genes. Indeed, in a preliminary experiment to address this idea we analyzed genome-wide RNA expression data using the Consortium samples to test the expression association between SHP (the single gene predictor) and a number of its known target genes, including Cyclin D1, Glut4, MTP, and PGC-1α. We found that SHP expression was associated with expression of three out of the four targets: Cyclin D1, Glut4, and MTP (*p*<0.0001).

A third noteworthy finding from our study was the ability to predict overall survival based on NR expression in normal tissue of patients with lung cancer. Whether this may be due to a “field effect” through changes in the epithelium, to induction in a paracrine fashion by the cancer, or to some other feature of normal lung epithelium is unknown. However, this finding does suggest that interrogating the histologically normal tissue may yield insight into lung cancer oncogenesis. To that end we note that the prognostic NR signature in normal tissue is completely different than that of the adjacent tumor. In contrast to that observed in tumors, the NR signature, as distilled using RPART analysis, revealed that NGFIB3 (NR4A1) and MR are single gene biomarkers found in normal tissue for predicting disease recurrence and overall survival time, respectively. NR4A family members have been shown to be tumor suppressors in a mouse model of myeloid leukemogenesis [48]. Similarly, low expression of MR has been shown to correlate with colorectal carcinoma recurrence [49]. These studies support the notion that higher expression of NR4A1 and MR might play a protective role against lung tumor pathogenesis.

A fourth finding of our study was the independent demonstration that the NR gene signature could be tested and cross-validated using two different gene expression platforms, QPCR and microarray. Given that microarray data do not have the dynamic or quantitative properties of data generated by QPCR, the cross-validation of the NR gene signature between different platforms strongly supports the idea that the NR superfamily may be a powerful prognostic predictor that also is functionally involved in lung cancer pathophysiology. Our results also suggest that a combination of a more robust collection process (microdissection instead of tissue mass) together with more quantitative measurements (QPCR instead of microarray) may reduce variability and strengthen the data. Indeed, the 95% CIs of HRs for both SHP and PR genes from the 30-patient dataset were smaller than those from Consortium data (with a sample size of 442) (Figure 4C and 4D). HRs of the high-risk versus low-risk group, defined using unsupervised cluster results, were also higher for the 30-patient dataset (Figure 2B) than for the Consortium data (Figure S11). Thus, while labor intensive, improving sample homogeneity and the quality of the expression data is likely to provide more reliable prognostic information.

Finally, the NR expression profile provides specific, testable hypotheses on the role of the NRs in lung cancer pathogenesis. For example, blocking the function of a highly expressed tumor cell NR could inhibit tumor growth or development, while over-expressing a low-abundance tumor cell NR could test its tumor suppressive capability. The finding that non-neoplastic tissue within the vicinity of the tumor also provided an NR gene signature that was predictive for survival time may provide the basis for testing NR function in the normal lung epithelium airway field where lung tumors develop. Perhaps one of the most surprising observations from this study is that an NR signature has not appeared in the prognostic signatures obtained in any of the previous global gene expression studies. This is true in spite of the fact that, at least in the multi-site Consortium database we analyzed, excerpting just the NR expression information yielded a predictive NR gene signature that was not discovered using global gene analysis [3],[6]. Thus, our study provides a strategic rationale for using an informed candidate gene profiling approach to identify prognostic markers and to interrogate specific gene families that may play roles in the cancer biology.

## Supporting Information

Figure S1 Patient survival time comparison between the selected 30 patients and the 379 patients from MDACC tissue bank. There was no significant difference in survival time between the two cohorts. Black line: large dataset, 310 patients, of which 127 died. Red line: small dataset, 30 patients, of which 16 died. Open circles indicate censored samples.
(0.09 MB PDF)

Figure S2 Expression profiles of the NR superfamily in lung tissues. Quantitative real-time PCR analysis was performed for 48 NRs (including two common splice variants each for PPARγ and PPARδ) in 30 pair-matched tissues (normal and tumor) from patients with lung cancer. Relative expression values were obtained as described in Methods. Ct>34 was scored as below detection. Open and filled bars represent normal and pair-matched tumor tissues from each patient, respectively. The patients are numbered from 1 to 30 (see Table S3) and grouped according to gender and survival status, with each patient being in the same position for each NR dataset.
(0.92 MB PDF)

Figure S3 CDF of the *p*-values of 48 NRs. The CDF represents associations between individual NR gene expression and survival from univariate Cox models from MDACC lung cancer cohort. Each dot represents a *p*-value for one NR gene, and the solid red line represents the expected CDF for randomly picked genes. The dashed green line corresponds to *p* = 0.05. Thirty-seven NR genes have *p*-values smaller than 0.05. SHP and PR genes are indicated in the plot.
(0.09 MB PDF)

Figure S4 Kaplan-Meier plot showing the predictive power of the NR gene signature in an additional set of adenocarcinomas. The NR gene expression signature developed from the QPCR dataset (MDACC cohort, *n* = 30) was validated in the microarray data from a cohort of 117 independent adenocarcinomas from Tomida et al. [18]. *p*-Values were obtained by the log-rank test. Red and black lines represent predicted high- and low-risk groups, respectively. Open circles indicate censored samples.
(0.13 MB PDF)

Figure S5 Kaplan-Meier plots showing the predictive power of the NR gene signature between lung adenocarcinomas and squamous cell carcinomas. Independent validation of the 48-NR gene expression signature was performed using the Consortium cohort (*n* = 442) as a training set and testing it in the microarray data from a cohort of 130 squamous cell carcinomas taken from Raponi et al. [19] (A), and vice versa (B). *p*-Values were obtained by the log-rank test. Red and black lines represent predicted high- and low-risk groups, respectively. Open circles indicate censored samples.
(0.25 MB PDF)

Figure S6 The histograms for *p*-values of 1,000 randomly selected lists of 48 genes. The histogram represents the association between the predicted risk groups and survival outcomes. The *p*-values were derived from prediction models built from 1,000 random lists, each comprising 48 genes in the Consortium training set and validated in the Consortium testing set. Only 28 random lists reach the significance level of 0.018 attained by the NR signature. Thus, the empirical *p*-value of 0.028 for the permutation test confirms the specificity of the NR signature.
(0.06 MB PDF)

Figure S7 Kaplan-Meier estimates of survival time based on NR expression when clinical variables are included in the analysis. The analysis for survival time was performed for clinical variables in the absence (A) or presence (B) of NR expression. The microarray dataset from the four-institute Consortium was divided into two groups, one for the training cohort and the other for the testing cohort. The analysis included the two principal component sets of 48 NR expression variables and clinical variables. The clinical variables included gender, age, stage, and treatments (i.e., those receiving adjuvant chemotherapy or not, and those receiving adjuvant radiation therapy or not) as co-variables in classification tree model. The final predictive tree structure can be seen in the Sweave report (Text S2). The predictive model was built in the training cohort and then validated in the testing cohort. In the testing cohort, patients in the predicted high-risk group live for a significantly shorter time than patients in the predicted low-risk group, (HR = 2.71, p = 1.02×10^−5^ for using clinical variables only; HR = 3.21, p = 2.43×10^−7^ for using clinical and NR signature). *p*-values were obtained by log-rank test. Red and black lines represent predicted high- and low-risk groups, respectively. Open circles indicate censored samples.
(0.14 MB PDF)

Figure S8 Classification tree structures built from MDACC and Consortium datasets. (A) Decision tree model built from MDACC samples using the NR gene expression signature. The gene expression of each patient was measured by QPCR. Seventeen patients within the high-risk group (SHP expression <−8.456) and 13 patients within the low-risk group (SHP expression ≥−8.456) are predicted by the decision tree. Sixteen out of 17 patients in the high-risk group have events, with an estimated rate of 2.27, while only one out of 13 patients in the low-risk group have events, with an estimated rate of 1.74. (B) Decision tree model built from MDACC samples using NR gene expression signature after removal of the SHP gene. Seventeen patients within the high-risk group (PR expression <−4.9) and 13 patients within the low-risk group (PR expression ≥4.9) are predicted by the decision tree. Thirteen out of 13 patients in the high-risk group have events, with an estimated rate of 2.90, while only four out of 17 patients in the low-risk group have events, with an estimated rate of 0.353.
(0.05 MB PDF)

Figure S9 Identification of NRs as prognostic biomarkers in normal lung tissue from patients with lung cancer. Kaplan-Meier plots of time to recurrence and survival are shown for NGFIB3 and MR, respectively. Note that these two plots are identical to those obtained when using the entire 48-NR gene set. (A) LOOCV of recursive partitioning tree model of the MDACC QPCR data in normal tissues shows that NGFIB3 is the single gene left in the predictive model for disease progression (HR = 4.61, 95% CI, 1.74–12.3; *p* = 0.00099). (B) Similar LOOCV analysis shows MR is a single gene predictor of the entire 48-NR gene set as associated with patient survival (HR = 2.22, 95% CI, 0.85–5.81; *p* = 0.094). Red and black lines represent high- and low-risk groups, respectively. Open circles indicate censored samples.
(0.13 MB PDF)

Figure S10 Scatter plots showing the correlations of NR gene expression between tumor and adjacent normal samples taken from either the MDACC (A) or Landi et al. (B) datasets. The *x*- and *y*-axes represent the log_2_ transformed expression values for normal and tumor samples, respectively. The figures show that NR expression in tumor samples is significantly correlated with that in the adjacent normal tissue. Pearson correlations were 0.87 (*p*<0.001) for the MDACC dataset and 0.92 (*p*<0.001) for the Landi et al. [28] dataset.
(1.36 MB PDF)

Figure S11 Kaplan-Meier plots of survival time of the Consortium cohort based on the 48 NR expression signatures. Unsupervised hierarchical cluster analysis of the microarray signature of the 48 NRs divides the 442 Consortium samples into two clusters. *p*-values were obtained using the log-rank test. Red and black lines represent high- and low-risk groups, respectively, and were derived from the unsupervised clustering algorithm using the NR gene signature. Open circles indicate censored samples.
(0.17 MB PDF)

Table S1 Comparison of patient characteristics between the selected 30 samples and the whole 379 samples of the MDACC lung tumor collection.
(0.04 MB PDF)

Table S2 Summary of patient clinical information.
(0.04 MB PDF)

Table S3 Clinical information on individual patients.
(0.06 MB PDF)

Table S4 Summary of NR expression data in normal and tumor lung tissue taken from lung cancer patients.
(0.05 MB PDF)

Table S5 Univariate Cox regression results for MDACC data.
(0.05 MB PDF)

Text S1 Supplementary methods.
(0.02 MB PDF)

Text S2 Sweave document. Nuclear receptor expression profiling defines a set of prognostic biomarkers for lung cancer.
(0.59 MB PDF)

## Abbreviations

CDF: cumulative distribution function
CI: confidence interval
HR: hazard ratio
LOOCV: leave-one-out cross-validation
MDACC: MD Anderson Cancer Center
MR: mineralocorticoid receptor
NR: nuclear receptor
NCI: National Cancer Institute
NSCLC: non-small-cell lung cancer
PR: progesterone receptor
QPCR: quantitative PCR
RPART: Recursive Partitioning and Regression Trees
SHP: short heterodimer partner
