# Supplementary material for: Nuclear Receptor Expression Defines a Set of Prognostic Biomarkers for Lung Cancer
Source: PLoS Med. 2010 Dec 14;7(12):e1000378. doi: 10.1371/journal.pmed.1000378 (PMC3001894; doi:10.1371/journal.pmed.1000378)
Supplement: Figure S1 — Patient survival time comparison between the selected 30 patients and the 379 patients from MDACC tissue bank. There was no significant difference in survival time between the two cohorts. Black line: large dataset, 310 patients, of which 127 died. Red line: small dataset, 30 patients, of which 16 died. Open circles indicate censored samples. (0.09 MB PDF) [file pmed.1000378.s001.pdf]

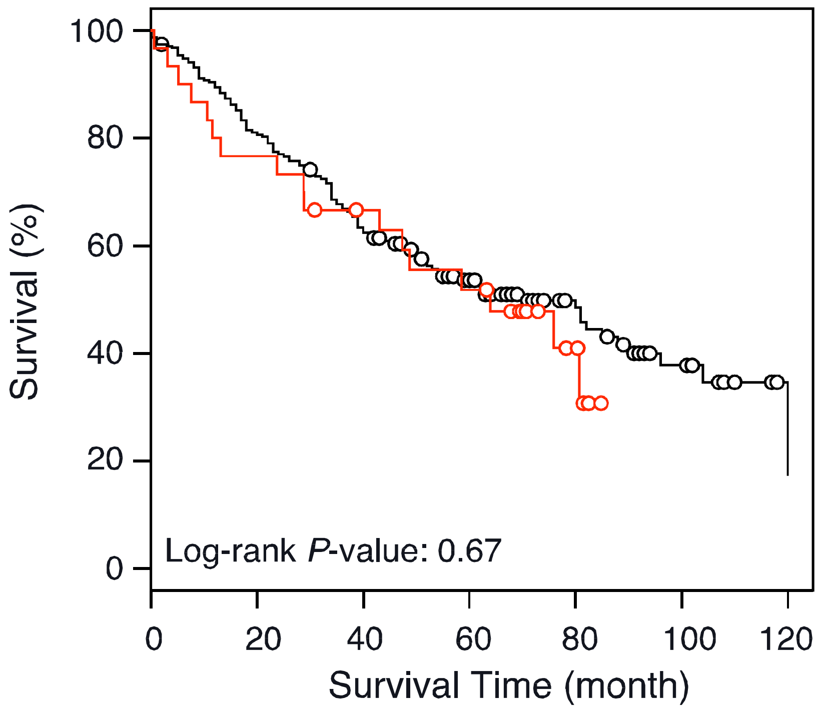

Figure S1. Patients' survival time comparison between the selected 30 patients and 379 patients from MDACC tissue bank.
There was no significant difference of survival time between the two cohorts. Black line: large dataset, 310 patients, 127 dead. Red line: small dataset, 30 patients, 16 dead. Open circles indicated censored samples.
