# Supplementary material for: Nuclear Receptor Expression Defines a Set of Prognostic Biomarkers for Lung Cancer
Source: PLoS Med. 2010 Dec 14;7(12):e1000378. doi: 10.1371/journal.pmed.1000378 (PMC3001894; doi:10.1371/journal.pmed.1000378)
Supplement: Figure S2 — Expression profiles of the NR superfamily in lung tissues. Quantitative real-time PCR analysis was performed for 48 NRs (including two common splice variants each for PPARγ and PPARδ) in 30 pair-matched tissues (normal and tumor) from patients with lung cancer. Relative expression values were obtained as described in Methods. Ct>34 was scored as below detection. Open and filled bars represent normal and pair-matched tumor tissues from each patient, respectively. The patients are numbered from 1 to 30 (see Table S3) and grouped according to gender and survival status, with each patient being in the same position for each NR dataset. (0.92 MB PDF) [file pmed.1000378.s002.pdf]

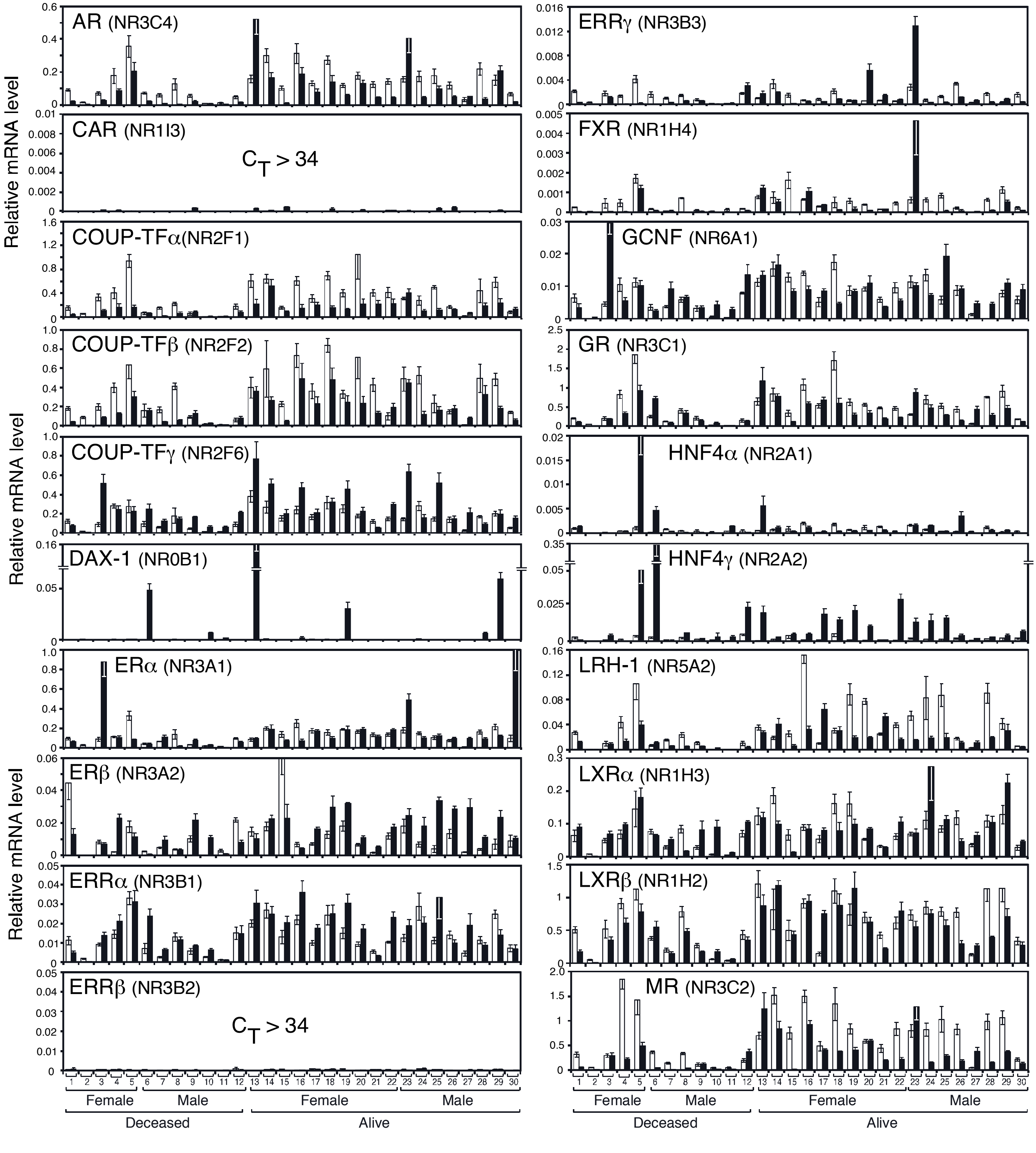

Figure S2. Expression profiles of the NR superfamily in lung tissues.
Quantitative real-time PCR analysis was performed for 48 NRs (including 2 common splice variants each for PPARγ and PPARδ) in 30 pair-matched tissues (normal and tumor) from lung cancer patients. Relative expression values were obtained as described in Methods. Ct > 34 was scored as below detection. Open and filled bars represent normal and pair-matched tumor tissues from each patient, respectively. The patients are numbered from 1–30 (see Table S3) and grouped according to gender and survival status with each patient being in the same position for each NR dataset.

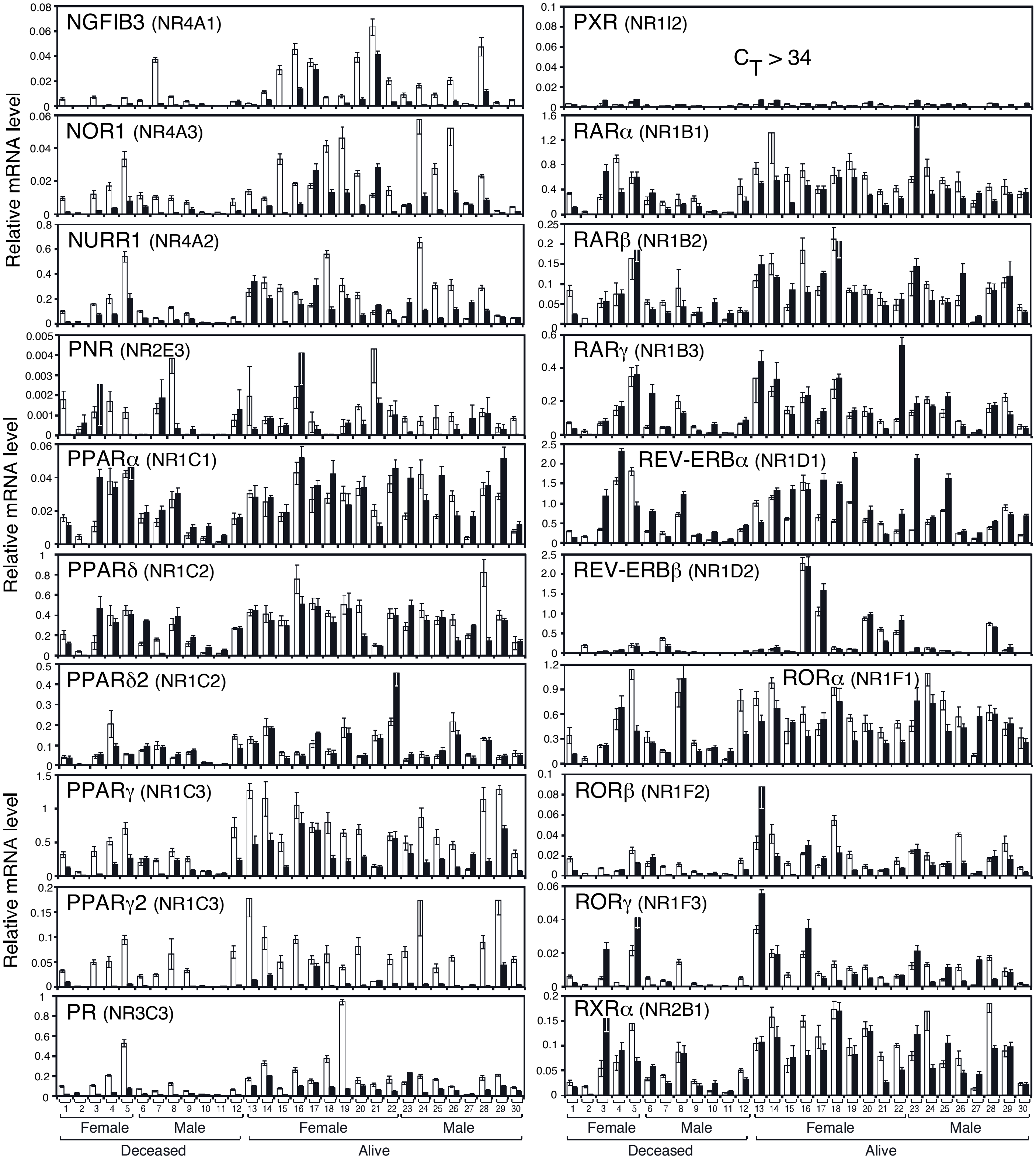

Figure S2 (continued). Expression profiles of the NR superfamily in lung tissues.
Quantitative real-time PCR analysis was performed for 48 NRs (including 2 common splice variants each for PPARγ and PPARδ) in 30 pair-matched tissues (normal and tumor) from lung cancer patients. Relative expression values were obtained as described in Methods. Ct > 34 was scored as below detection. Open and filled bars represent normal and pair-matched tumor tissues from each patient, respectively. The patients are numbered from 1–30 (see Table S3) and grouped according to gender and survival status with each patient being in the same position for each NR dataset.

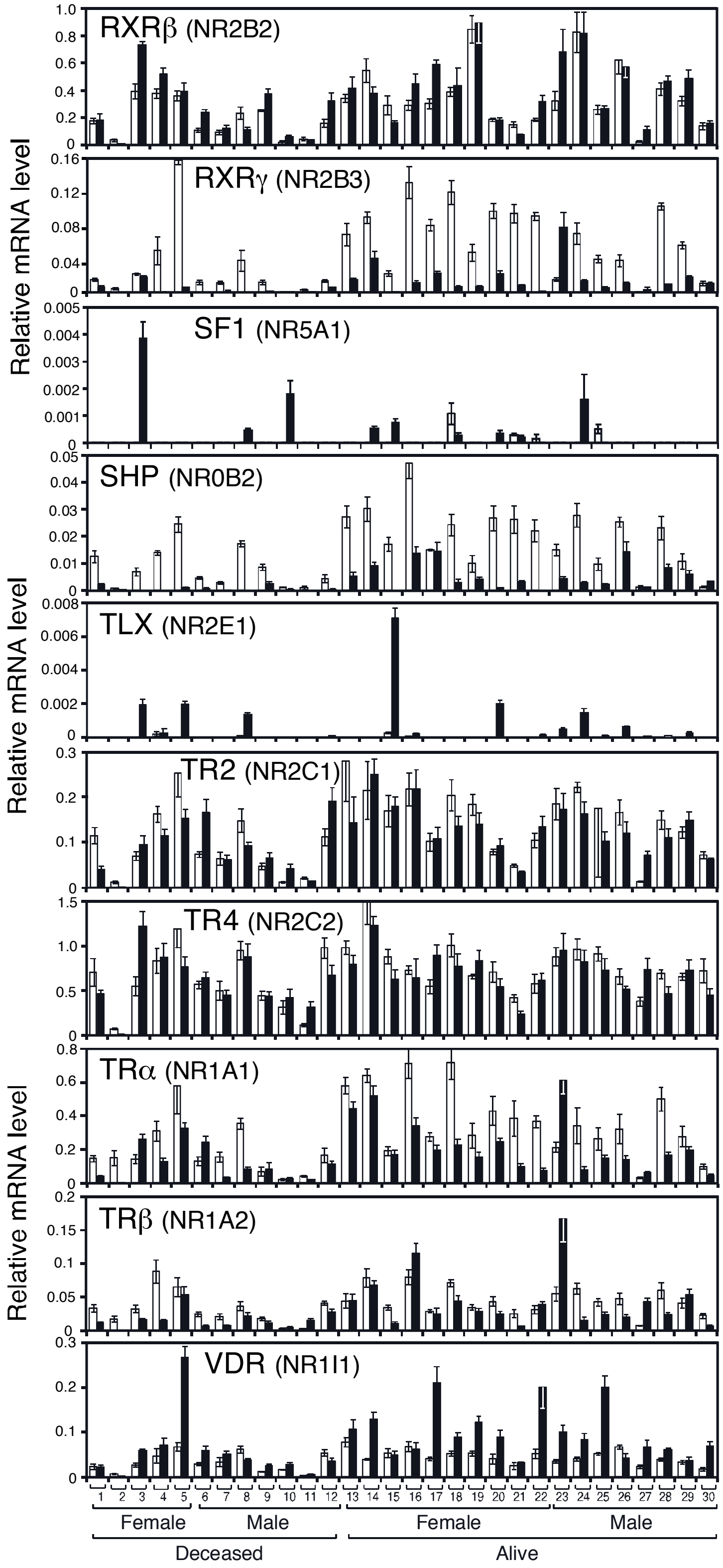

Figure S2 (continued). Expression profiles of the NR superfamily in lung tissues.
Quantitative real-time PCR analysis was performed for 48 NRs (including 2 common splice variants each for PPARγ and PPARδ) in 30 pair-matched tissues (normal and tumor) from lung cancer patients. Relative expression values were obtained as described in Methods. Ct > 34 was scored as below detection. Open and filled bars represent normal and pair-matched tumor tissues from each patient, respectively. The patients are numbered from 1–30 (see Table S3) and grouped according to gender and survival status with each patient being in the same position for each NR dataset.
