# Supplementary material for: Nuclear Receptor Expression Defines a Set of Prognostic Biomarkers for Lung Cancer
Source: PLoS Med. 2010 Dec 14;7(12):e1000378. doi: 10.1371/journal.pmed.1000378 (PMC3001894; doi:10.1371/journal.pmed.1000378)
Supplement: Figure S3 — CDF of the p-values of 48 NRs. The CDF represents associations between individual NR gene expression and survival from univariate Cox models from MDACC lung cancer cohort. Each dot represents a p-value for one NR gene, and the solid red line represents the expected CDF for randomly picked genes. The dashed green line corresponds to p = 0.05. Thirty-seven NR genes have p-values smaller than 0.05. SHP and PR genes are indicated in the plot. (0.09 MB PDF) [file pmed.1000378.s003.pdf]

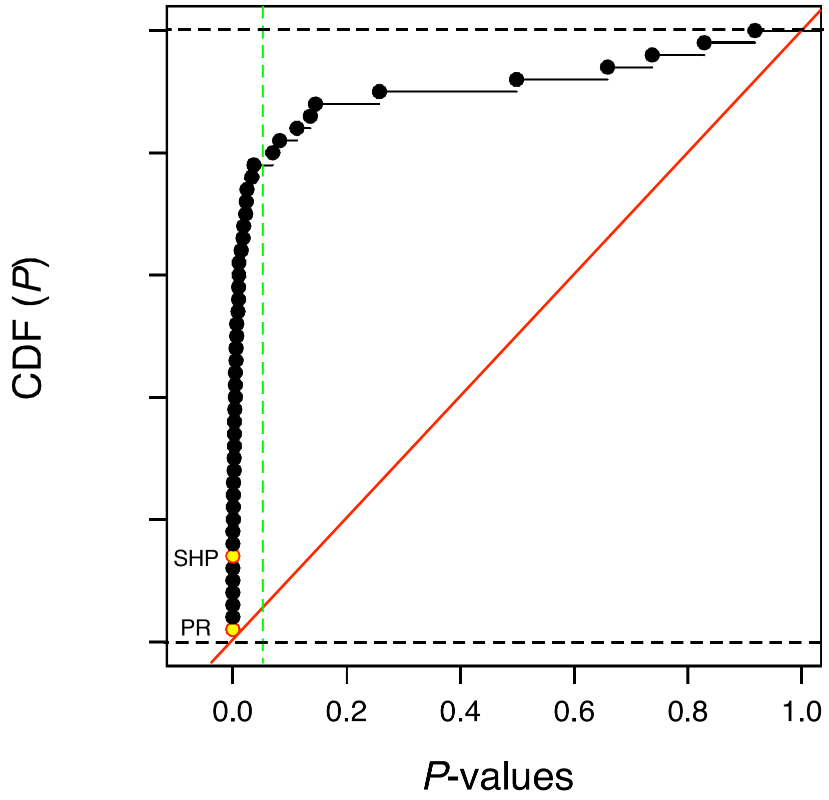

Figure S3. Cumulative distribution function (CDF) of the *P*-values of 48 nuclear receptors.
The CDF represents associations between the individual NR gene expression and survival from univariate Cox models from MDACC lung cancer cohort. Each dot represents a *P*-value for one NR gene and the solid red line represents the expected CDF for randomly picked genes. The dashed green line corresponds to *P*=0.05. 37 NR genes have *P*-values smaller than 0.05. SHP and PR genes are indicated in the plot.
