# Supplementary material for: Nuclear Receptor Expression Defines a Set of Prognostic Biomarkers for Lung Cancer
Source: PLoS Med. 2010 Dec 14;7(12):e1000378. doi: 10.1371/journal.pmed.1000378 (PMC3001894; doi:10.1371/journal.pmed.1000378)
Supplement: Figure S4 — Kaplan-Meier plot showing the predictive power of the NR gene signature in an additional set of adenocarcinomas. The NR gene expression signature developed from the QPCR dataset (MDACC cohort, n = 30) was validated in the microarray data from a cohort of 117 independent adenocarcinomas from Tomida et al. [18]. p-Values were obtained by the log-rank test. Red and black lines represent predicted high- and low-risk groups, respectively. Open circles indicate censored samples. (0.13 MB PDF) [file pmed.1000378.s004.pdf]

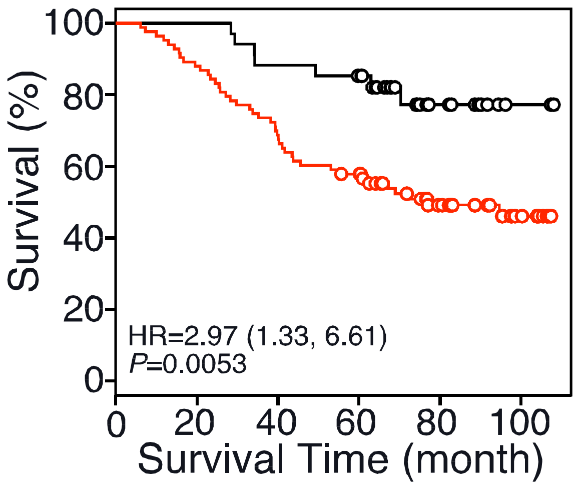

Figure S4. Kaplan-Meier plot showing the predictive power of the NR gene signature in an additional set of adenocarcinomas.
The NR gene-expression signature developed from the QPCR dataset (MDACC cohort, n=30) was validated in the microarray data from a cohort of 117 independent adenocarcinomas from Tomida et al. (18). *P*-values were obtained by the log-rank test. Red and black lines represent predicted high- and low-risk groups, respectively. Open circles indicate censored samples.
