# Supplementary material for: Nuclear Receptor Expression Defines a Set of Prognostic Biomarkers for Lung Cancer
Source: PLoS Med. 2010 Dec 14;7(12):e1000378. doi: 10.1371/journal.pmed.1000378 (PMC3001894; doi:10.1371/journal.pmed.1000378)
Supplement: Figure S5 — Kaplan-Meier plots showing the predictive power of the NR gene signature between lung adenocarcinomas and squamous cell carcinomas. Independent validation of the 48-NR gene expression signature was performed using the Consortium cohort (n = 442) as a training set and testing it in the microarray data from a cohort of 130 squamous cell carcinomas taken from Raponi et al. [19] (A), and vice versa (B). p-Values were obtained by the log-rank test. Red and black lines represent predicted high- and low-risk groups, respectively. Open circles indicate censored samples. (0.25 MB PDF) [file pmed.1000378.s005.pdf]

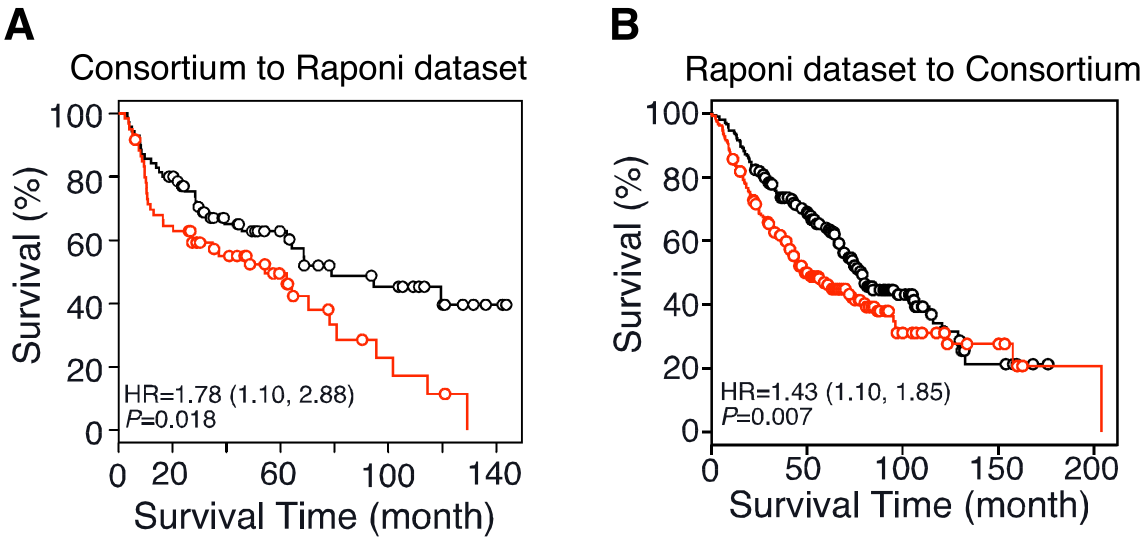

Figure S5. Kaplan-Meier plots showing the predictive power of the NR gene signature between lung adenocarcinomas and squamous cell carcinomas. Independent validation of the 48 NR gene-expression signature was performed using the Consortium cohort (n=442) as a training set and testing it in the microarray data from a cohort of 130 squamous cell carcinomas taken from Raponi et al. (19). (A), and vice versa (B). *P*-values were obtained by the log-rank test. Red and black lines represent predicted high- and low-risk groups, respectively. Open circles indicate censored samples.
