# Supplementary material for: Nuclear Receptor Expression Defines a Set of Prognostic Biomarkers for Lung Cancer
Source: PLoS Med. 2010 Dec 14;7(12):e1000378. doi: 10.1371/journal.pmed.1000378 (PMC3001894; doi:10.1371/journal.pmed.1000378)
Supplement: Figure S6 — The histograms for p-values of 1,000 randomly selected lists of 48 genes. The histogram represents the association between the predicted risk groups and survival outcomes. The p-values were derived from prediction models built from 1,000 random lists, each comprising 48 genes in the Consortium training set and validated in the Consortium testing set. Only 28 random lists reach the significance level of 0.018 attained by the NR signature. Thus, the empirical p-value of 0.028 for the permutation test confirms the specificity of the NR signature. (0.06 MB PDF) [file pmed.1000378.s006.pdf]

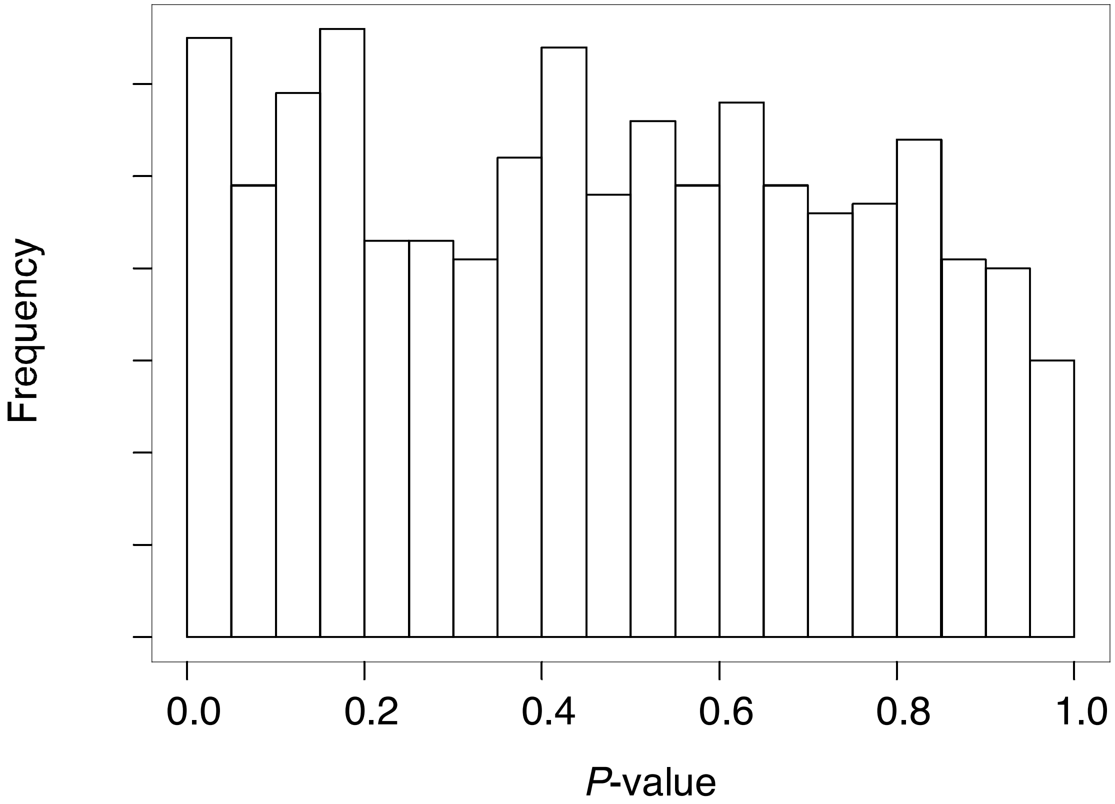

Figure S6. The histograms for *P*-values of 1000 randomly selected lists of 48 genes. The histogram represents the association between the predicted risk groups and survival outcomes. The *P*-values were derived from prediction models built from 1000 random lists each comprising 48 genes in the Consortium training set and validated in the Consortium testing set. Only 28 random lists reach the significance level of 0.018 for the NR signature. Thus, the empirical *P*-value of 0.028 for the permutation test confirms the specificity of the NR signature.
