# Supplementary material for: Nuclear Receptor Expression Defines a Set of Prognostic Biomarkers for Lung Cancer
Source: PLoS Med. 2010 Dec 14;7(12):e1000378. doi: 10.1371/journal.pmed.1000378 (PMC3001894; doi:10.1371/journal.pmed.1000378)
Supplement: Figure S7 — Kaplan-Meier estimates of survival time based on NR expression when clinical variables are included in the analysis. The analysis for survival time was performed for clinical variables in the absence (A) or presence (B) of NR expression. The microarray dataset from the four-institute Consortium was divided into two groups, one for the training cohort and the other for the testing cohort. The analysis included the two principal component sets of 48 NR expression variables and clinical variables. The clinical variables included gender, age, stage, and treatments (i.e., those receiving adjuvant chemotherapy or not, and those receiving adjuvant radiation therapy or not) as co-variables in classification tree model. The final predictive tree structure can be seen in the Sweave report (Text S2). The predictive model was built in the training cohort and then validated in the testing cohort. In the testing cohort, patients in the predicted high-risk group live for a significantly shorter time than patients in the predicted low-risk group, (HR = 2.71, p = 1.02×10^−5^ for using clinical variables only; HR = 3.21, p = 2.43×10^−7^ for using clinical and NR signature). p-values were obtained by log-rank test. Red and black lines represent predicted high- and low-risk groups, respectively. Open circles indicate censored samples. (0.14 MB PDF) [file pmed.1000378.s007.pdf]

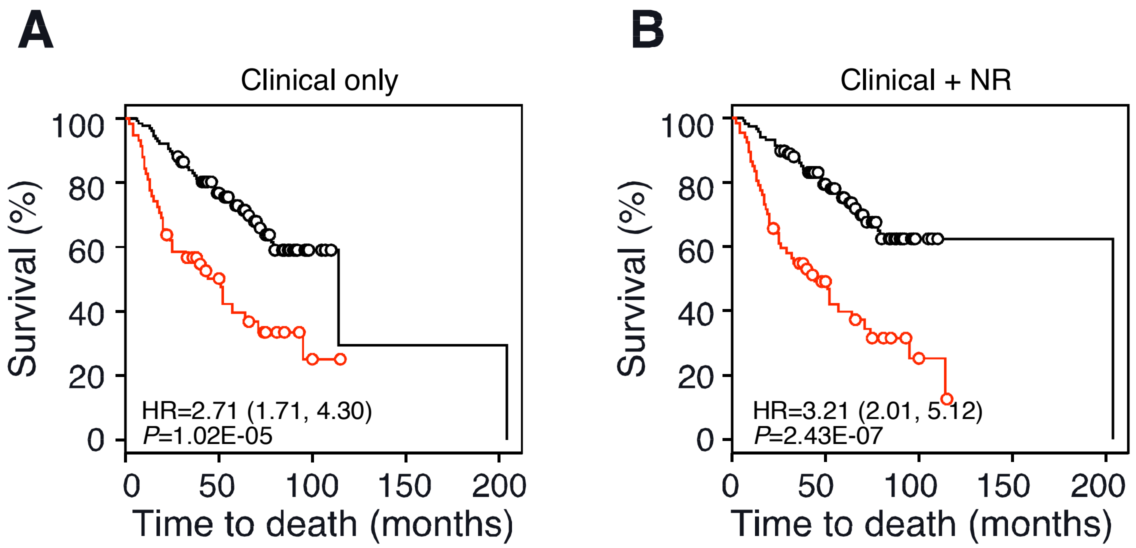

Figure S7. Kaplan-Meier estimates of survival time based on NR expression when clinical variables are included in the analysis.
The analysis for survival time was performed for clinical variables in the absence (A) or presence (B) of NR expression. The microarray dataset from the four institute Consortium was divided into two groups, one for the training cohort and the other for the testing cohort. The analysis included the two principal component sets of 48 NR expression variables and clinical variables. The clinical variables include gender, age, stage, treatments (i.e., those receiving adjuvant chemotherapy or not, and those receiving adjuvant radiation therapy or not) as co-variables in classification tree model. The final predictive tree structure can be seen in the Sweave report (Supplemental information online). The predictive model was built in the training cohort and then validated in the testing cohort. In the testing cohort, patients in the predicted high risk group live for significantly shorter time than patients in the predicted low risk group, (HR = 2.71, P = 1.02×10^{-5} for using clinical variables only; HR = 3.21, P = 2.43×10^{-7} for using clinical and NR signature). P-values were obtained by log-rank test. Red and black lines represent predicted high and low-risk groups, respectively. Open circles indicated censored samples.
