# Supplementary material for: Nuclear Receptor Expression Defines a Set of Prognostic Biomarkers for Lung Cancer
Source: PLoS Med. 2010 Dec 14;7(12):e1000378. doi: 10.1371/journal.pmed.1000378 (PMC3001894; doi:10.1371/journal.pmed.1000378)
Supplement: Figure S8 — Classification tree structures built from MDACC and Consortium datasets. (A) Decision tree model built from MDACC samples using the NR gene expression signature. The gene expression of each patient was measured by QPCR. Seventeen patients within the high-risk group (SHP expression <−8.456) and 13 patients within the low-risk group (SHP expression ≥−8.456) are predicted by the decision tree. Sixteen out of 17 patients in the high-risk group have events, with an estimated rate of 2.27, while only one out of 13 patients in the low-risk group have events, with an estimated rate of 1.74. (B) Decision tree model built from MDACC samples using NR gene expression signature after removal of the SHP gene. Seventeen patients within the high-risk group (PR expression <−4.9) and 13 patients within the low-risk group (PR expression ≥4.9) are predicted by the decision tree. Thirteen out of 13 patients in the high-risk group have events, with an estimated rate of 2.90, while only four out of 17 patients in the low-risk group have events, with an estimated rate of 0.353. (0.05 MB PDF) [file pmed.1000378.s008.pdf]

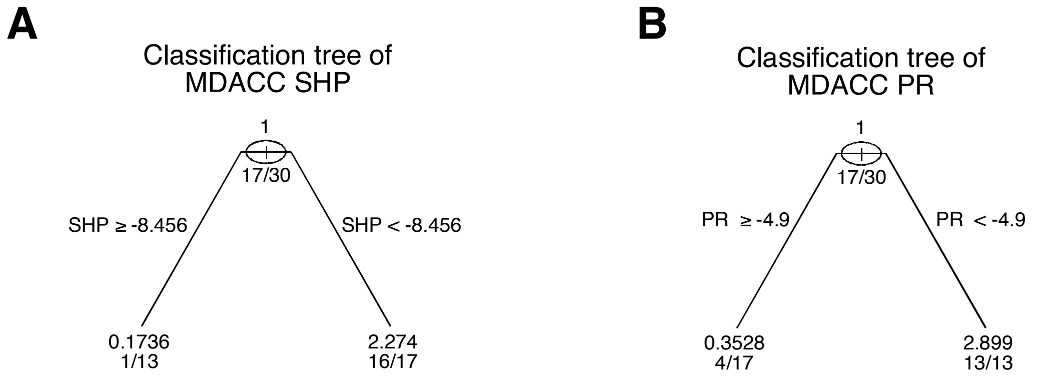

Figure S8. Classification tree structures built from MDACC and Consortium datasets.
(A) Decision tree model built from MDACC samples using the NR gene expression signature. The gene expression of each patient was measured by QPCR. Seventeen patients within the high risk group (SHP expression < -8.456) and 13 patients within the low risk group (SHP expression ≥ -8.456) are predicted by the decision tree. Sixteen out of 17 patients in the high risk group have events with an estimated rate of 2.27, while only 1 out of 13 patients in the low risk group have events with an estimated rate of 1.74.
(B) Decision tree model built from MDACC samples using NR gene expression signature after removal of the SHP gene. Seventeen patients within the high risk group (PR expression < -4.9) and 13 patients within the low risk group (PR ≥ 4.9) are predicted by the decision tree. Thirteen out of 13 patients in the high risk group have events with an estimated rate of 2.90, while only 4 out of 17 patients in the low risk group have events with an estimated rate of 0.353.
