# Supplementary material for: Nuclear Receptor Expression Defines a Set of Prognostic Biomarkers for Lung Cancer
Source: PLoS Med. 2010 Dec 14;7(12):e1000378. doi: 10.1371/journal.pmed.1000378 (PMC3001894; doi:10.1371/journal.pmed.1000378)
Supplement: Figure S9 — Identification of NRs as prognostic biomarkers in normal lung tissue from patients with lung cancer. Kaplan-Meier plots of time to recurrence and survival are shown for NGFIB3 and MR, respectively. Note that these two plots are identical to those obtained when using the entire 48-NR gene set. (A) LOOCV of recursive partitioning tree model of the MDACC QPCR data in normal tissues shows that NGFIB3 is the single gene left in the predictive model for disease progression (HR = 4.61, 95% CI, 1.74–12.3; p = 0.00099). (B) Similar LOOCV analysis shows MR is a single gene predictor of the entire 48-NR gene set as associated with patient survival (HR = 2.22, 95% CI, 0.85–5.81; p = 0.094). Red and black lines represent high- and low-risk groups, respectively. Open circles indicate censored samples. (0.13 MB PDF) [file pmed.1000378.s009.pdf]

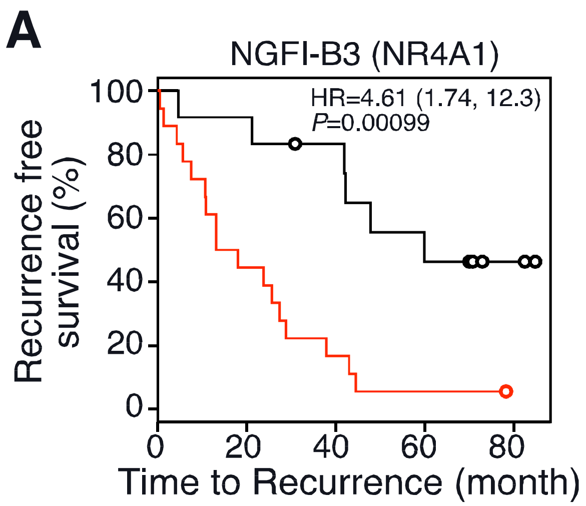

Figure S9. Identification of NRs as prognostic biomarkers in normal lung tissue from lung cancer patients.

Kaplan-Meier plots of time to recurrence and survival are shown for NGFI-B and MR, respectively. Note that these two plots are identical to those obtained when using the entire 48 NR gene set. (A) LOOCV of recursive-partitioning tree model of the MDACC QPCR data in normal tissues shows that NGFI-B is the single gene left in the predictive model for disease progression (HR = 4.61, 95% CI 1.74 to 12.3; P = 0.00099). (B) Similar LOOCV analysis shows MR is a single-gene predictor of the entire 48 NR gene set as associated with patient survival (HR = 2.22, 95% CI 0.85 to 5.81; P = 0.094). Red and black lines represent high- and low-risk groups, respectively. Open circles indicate censored samples.
