# Supplementary material for: Nuclear Receptor Expression Defines a Set of Prognostic Biomarkers for Lung Cancer
Source: PLoS Med. 2010 Dec 14;7(12):e1000378. doi: 10.1371/journal.pmed.1000378 (PMC3001894; doi:10.1371/journal.pmed.1000378)
Supplement: Figure S10 — Scatter plots showing the correlations of NR gene expression between tumor and adjacent normal samples taken from either the MDACC (A) or Landi et al. (B) datasets. The x- and y-axes represent the log2 transformed expression values for normal and tumor samples, respectively. The figures show that NR expression in tumor samples is significantly correlated with that in the adjacent normal tissue. Pearson correlations were 0.87 (p<0.001) for the MDACC dataset and 0.92 (p<0.001) for the Landi et al. [28] dataset. (1.36 MB PDF) [file pmed.1000378.s010.pdf]

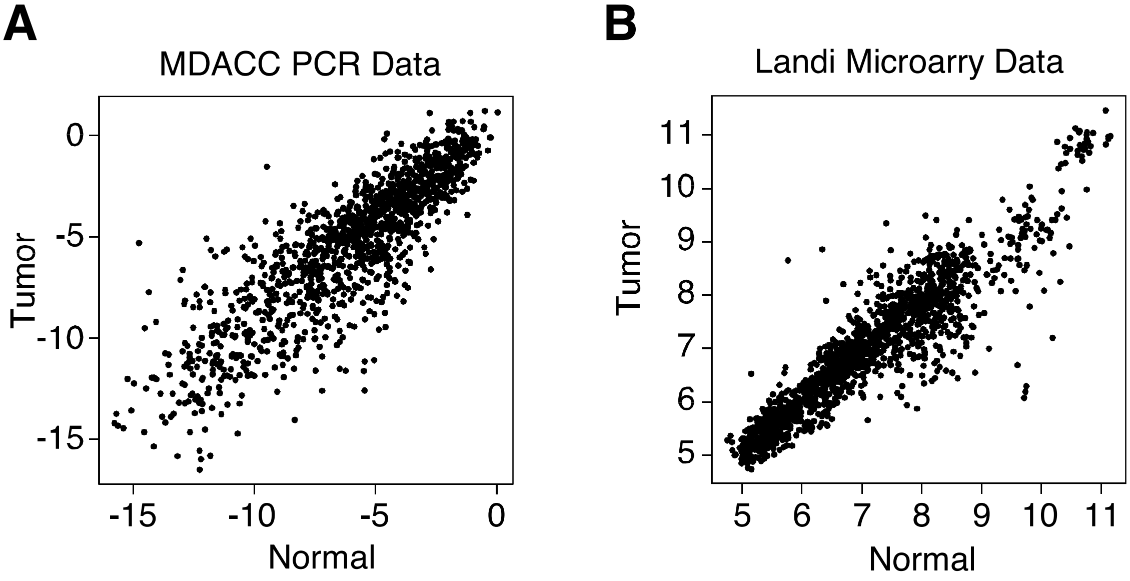

Figure S10. Scatter plots showing the correlations of NR gene expression between tumor and adjacent normal samples taken from either the MDACC (A) or Landi et al. (B) datasets.
The x- and y-axes represent the log2 transformed expression values for normal and tumor samples, respectively. The figures show that the NR expression in tumor samples is significantly correlated with that in the adjacent normal tissue. Pearson correlations were 0.87 ($P < 0.001$) for the MDACC dataset and 0.92 ($P < 0.001$) for the Landi et al. dataset (28).
