# Supplementary material for: Nuclear Receptor Expression Defines a Set of Prognostic Biomarkers for Lung Cancer
Source: PLoS Med. 2010 Dec 14;7(12):e1000378. doi: 10.1371/journal.pmed.1000378 (PMC3001894; doi:10.1371/journal.pmed.1000378)
Supplement: Figure S11 — Kaplan-Meier plots of survival time of the Consortium cohort based on the 48 NR expression signatures. Unsupervised hierarchical cluster analysis of the microarray signature of the 48 NRs divides the 442 Consortium samples into two clusters. p-values were obtained using the log-rank test. Red and black lines represent high- and low-risk groups, respectively, and were derived from the unsupervised clustering algorithm using the NR gene signature. Open circles indicate censored samples. (0.17 MB PDF) [file pmed.1000378.s011.pdf]

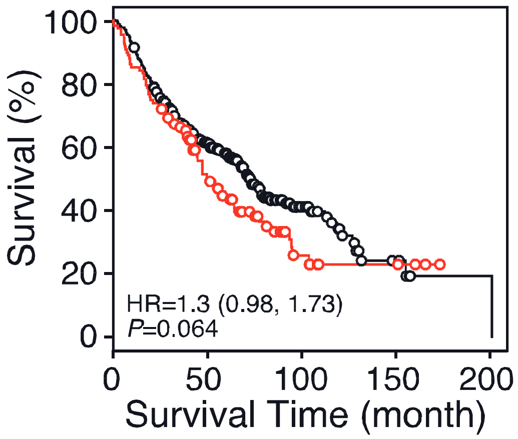

Figure S11. Kaplan-Meier plots of survival time of the Consortium cohort based on the 48 NR expression signatures.

Unsupervised hierarchical cluster analysis of the microarray signature of the 48 NRs divides the 442 Consortium samples into two clusters. *P*-values were obtained using the log-rank test. Red and black colors were defined by unsupervised clustering algorithm using NR gene signature. Open circles indicate censored samples.
