# Supplementary material for: Nuclear Receptor Expression Defines a Set of Prognostic Biomarkers for Lung Cancer
Source: PLoS Med. 2010 Dec 14;7(12):e1000378. doi: 10.1371/journal.pmed.1000378 (PMC3001894; doi:10.1371/journal.pmed.1000378)
Supplement: Table S1 — Comparison of patient characteristics between the selected 30 samples and the whole 379 samples of the MDACC lung tumor collection. (0.04 MB PDF) [file pmed.1000378.s012.pdf]

Table S1. Comparison of patients characteristics between the selected 30 samples and the whole 379 samples.

		Small dataset (n=30)	Big dataset (n=379)	*P* value
Gender	Female	15 (50%)	174 (45.9%)	0.7064
	Male	15 (50%)	205 (54.1%)	
Stage	I	17 (56.7%)	171 (49.6%)	0.0370
	II	4 (13.3%)	70 (20.3%)	
	III	5 (16.7%)	94 (27.3%)	
	IV	4 (13.3%)	10 (2.9%)	
Histology	SCC	8 (26.7%)	125 (32.8%)	0.5493
	ADC	22 (73.3%)	256 (67.2%)	

Abbreviations: ADC, adenocarcinoma; SCC, squamous cell carcinoma.
