# Supplementary material for: Nuclear Receptor Expression Defines a Set of Prognostic Biomarkers for Lung Cancer
Source: PLoS Med. 2010 Dec 14;7(12):e1000378. doi: 10.1371/journal.pmed.1000378 (PMC3001894; doi:10.1371/journal.pmed.1000378)
Supplement: Table S2 — Summary of patient clinical information. (0.04 MB PDF) [file pmed.1000378.s013.pdf]

Table S2. Summary of patient clinical information.

Feature	Cohort (n=30)
Age (y)	
Median	64.7
Range	44.0-77.7
Mean	63.3
Gender	
Female	15
Male	15
Race	
White	28
Black	1
Asian	1
TNM Stage	
I	17
II	4
III	5
IV	4
Tumor type	
ADC	22
SCC	8
Survival	
Dead	17
Female	7
Male	10
Alive	13
Female	8
Male	5
Smoking history†	
No	4
Yes	26
Adjuvant therapy	
No	27
Yes	3

Abbreviations: ADC, adenocarcinoma; SCC, squamous cell carcinoma; TNM, tumor size, node involvement, metastasis status.

†, Patients who had smoked at least 100 cigarettes in their lifetime were defined as smokers.
