# Supplementary material for: Nuclear Receptor Expression Defines a Set of Prognostic Biomarkers for Lung Cancer
Source: PLoS Med. 2010 Dec 14;7(12):e1000378. doi: 10.1371/journal.pmed.1000378 (PMC3001894; doi:10.1371/journal.pmed.1000378)
Supplement: Table S3 — Clinical information on individual patients. (0.06 MB PDF) [file pmed.1000378.s014.pdf]

Table S3. Clinical information on individual patients.

Sample ID		Age	Gender	Race	Tobacco history	Pack-Years	Stage	Death	Survival Time (months)	Progression	Time to Progression (months)	Pathology	N.A. therapy
1	847-ADC	60	F	W	Current	35	IB	Yes	0.5	Yes	0.5	T2,N0,M0	No
2	773-ADC	78	F	W	Current	50	IV	Yes	11.6	Yes	5.6	T4,N2,M1	No
3	848-ADC	48	F	W	Current	45	IB	Yes	23.7	Yes	23.7	T2,N0,M0	No
4	801-SCC	63	F	W	Current	60	IV	Yes	28.8	Yes	28.8	T4,N1,M1	No
5	845-ADC	58	F	W	Former	35	IIB	Yes	48.7	Yes	27.4	T2,N1,M0	No
6	947-ADC	68	M	W	Current	75	IIIB	Yes	3.1	Yes	1.2	T4,N0,M0	No
7	758-SCC	70	M	W	Former	8	IIIA	Yes	5.1	Yes	4.7	T2,N2,M0	No
8	857-SCC	71	M	W	Current	80	IB	Yes	7.5	Yes	7.5	T2,N0,M0	No
9	919-ADC	70	M	W	Current	70	IB	Yes	10.6	Yes	10.6	T2,N0,M0	No
10	878-SCC	63	M	W	Former	43	IIIB	Yes	13.1	Yes	13.1	T4,N2,M0	Yes
11	877-ADC	57	M	W	Current	75	IIB	Yes	28.8	Yes	18.0	T2,N1,M0	No
12	797-ADC	69	M	W	Current	77	IB	Yes	43.0	Yes	43.0	T2,N0,M0	No
13	896-ADC	49	F	W	Current	30	IA	No	38.7	Yes	25.6	T1,N0,M0	No
14	922-ADC	46	F	As	Never	0	IA	No	69.5	Yes	42.2	T1,N0,M0	No
15	799-SCC	62	F	W	Current	100	IB	Yes	47.3	Yes	21.1	T2,N0,M0	No
16	778-ADC	58	F	W	Former	20	IB	No	81.5	Yes	59.9	T2,N0,M0	No
17	764-ADC	58	F	W	Current	105	IB	No	70.8	No	70.8	T2,N0,M0	No
18	781-SCC	76	F	W	Current	56	IA	No	67.8	Yes	37.8	T1,N0,M0	No
19	803-ADC	65	F	B	Current	50	IIB	No	80.4	Yes	10.8	T2,N1,M0	No
20	739-ADC	73	F	W	Former	60	IIIA	Yes	63.9	Yes	47.7	T2,N2,M0	No
21	737-ADC	74	F	W	Current	40	IA	No	84.8	No	84.8	T1,N0,M0	No
22	749-ADC	70	F	W	Never	0	IB	No	30.9	No	30.9	T2,N0,M0	No
23	795-ADC	64	M	W	Never	0	IA	No	78.3	No	78.3	T1,N0,M0	No
24	794-SCC	67	M	W	Current	100	IIB	No	70.0	No	70.0	T2,N1,M0	No
25	792-ADC	68	M	W	Current	100	IA	Yes	80.7	Yes	41.9	T1,N0,M0	No
26	798-ADC	59	M	W	Current	84	IV	No	82.5	No	82.5	T2,N0,M1	No
27	818-ADC	44	M	W	Never	0	IV	Yes	75.9	Yes	13.1	T4,N1,M1	Yes
28	756-SCC	56	M	W	Current	85	IIIA	No	73.0	No	73.0	T1,N2,M0	No
29	782-ADC	69	M	W	Current	150	IA	Yes	58.5	Yes	4.3	T1,N0,M0	No
30	914-ADC	66	M	W	Former	80	IB	No	63.2	Yes	44.5	T2,N0,M0	No

Abbreviations: ADC, adenocarcinoma; SCC, squamous cell carcinoma; W, white; B, black; As, asian; TNM, tumor size, node involvement, metastasis status.
