# Supplementary material for: Nuclear Receptor Expression Defines a Set of Prognostic Biomarkers for Lung Cancer
Source: PLoS Med. 2010 Dec 14;7(12):e1000378. doi: 10.1371/journal.pmed.1000378 (PMC3001894; doi:10.1371/journal.pmed.1000378)
Supplement: Table S4 — Summary of NR expression data in normal and tumor lung tissue taken from lung cancer patients. (0.05 MB PDF) [file pmed.1000378.s015.pdf]

Table S4. Summary of NR expression data in normal and tumor lung tissue taken from lung cancer patients.

Broadly expressed in both normal and tumor (n=22)		Selectively expressed in either tumor or normal tissue			Low to undetectable* expression in normal and tumor (n=3)
		Tumor (n=7)	Normal (n=18)		
ERα	RARγ	COUP-TFγ	AR	PPARγ	CAR
ERRα	REV-ERBα	DAX-1	COUP-TFα	PPARγ2	ERRβ
GCNF	REV-ERBβ	ERβ	COUP-TFβ	PR	PXR
GR	RORα	HNF4α	ERRγ	RORβ	
LXRα	RXRα	HNF4γ	FXR	RORγ	
LXRβ	RXRβ	TLX	LRH-1	RXRγ	
PPARα	TR2	SF1	MR	SHP	
PPARδ	TR4		NGFIB3		
PPARδ2	TRα		NOR1		
RARα	TRβ		NURR1		
RARβ	VDR		PNR		

* Ct > 34.

Raw primary data can be found at www.NURSA.org.
