# Supplementary material for: Nuclear Receptor Expression Defines a Set of Prognostic Biomarkers for Lung Cancer
Source: PLoS Med. 2010 Dec 14;7(12):e1000378. doi: 10.1371/journal.pmed.1000378 (PMC3001894; doi:10.1371/journal.pmed.1000378)
Supplement: Table S5 — Univariate Cox regression results for MDACC data. (0.05 MB PDF) [file pmed.1000378.s016.pdf]

Table S5. Univariate Cox regression results for MDACC.

Nuclear Receptors	HR	*P*-value
PR	0.35	3.10E-05
RXRγ	0.32	4.86E-05
NOR1	0.39	7.52E-05
NURR1	0.42	8.16E-05
COUP-TFβ	0.42	2.24E-04
MR	0.39	2.35E-04
RORβ	0.48	4.47E-04
NGFIB3	0.35	4.85E-04
LRH1	0.48	6.14E-04
AR	0.36	6.80E-04
SHP	0.41	7.79E-04
PPARγ2	0.42	9.96E-04
RARα	0.51	1.81E-03
COUP-TFα	0.58	2.19E-03
RARβ	0.54	2.32E-03
ERRγ	0.4	2.64E-03
RORγ	0.46	3.16E-03
PPARγ	0.47	3.39E-03
LXRβ	0.54	3.68E-03
ERα	0.5	4.06E-03
PPARδ2	0.55	4.88E-03
FXR	0.34	5.19E-03
TRα	0.52	5.77E-03
VDR	0.55	6.20E-03
RORα	0.59	6.74E-03
GR	0.55	7.69E-03
RXRβ	0.57	9.46E-03
TRβ	0.62	1.07E-02
REV-ERBβ	0.52	1.39E-02
ERβ	0.62	1.58E-02
RXRα	0.58	1.64E-02
REV-ERBα	0.59	2.01E-02
PPARδ	0.59	2.05E-02
TR2	0.64	2.31E-02
RARγ	0.59	2.51E-02
PPARα	0.64	3.30E-02
COUP-TFγ	0.64	4.35E-02

Nuclear Receptors	HR	*P*-value
ERRα	0.66	5.48E-02
GCNF	0.65	8.26E-02
LXRα	0.68	9.23E-02
HNF4γ	0.62	1.21E-01
TR4	0.73	1.50E-01
PXR	0.76	1.58E-01
HNF4α	0.69	1.60E-01
PNR	0.77	2.92E-01
CAR	0.81	4.08E-01
ERRβ	0.91	6.82E-01
SF-1	1.08	7.67E-01
TLX	1.05	8.38E-01
DAX-1	0.99	9.58E-01
