# Supplementary material for: Nuclear Receptor Expression Defines a Set of Prognostic Biomarkers for Lung Cancer
Source: PLoS Med. 2010 Dec 14;7(12):e1000378. doi: 10.1371/journal.pmed.1000378 (PMC3001894; doi:10.1371/journal.pmed.1000378)
Supplement: Text S1 — Supplementary methods. (0.02 MB PDF) [file pmed.1000378.s017.pdf]

Text S1: Supplementary Methods

Sample selection procedure

Selection of 30 samples from the MDACC lung cancer tumor bank was performed in the following way: We started with all 118 of the microdissected samples available from the cases stored in the Lung SPORE tissue bank that were diagnosed with adenocarcinomas or squamous cell carcinomas and collected between March 2001 and August 2002. Among the 118 samples, 57 had the Lung SPORE consents available. All 57 cases were screened and 31 tumors were selected that exhibited >70% malignant cells based on examination at microdissection. One sample was excluded due to low RNA quality, which resulted in the 30 samples used for our analysis.

To investigate whether the samples used to develop the NR gene signature are representative of all lung cancer patient samples, we compared the characteristics and survival time of the 30 samples used in this study with 379 samples randomly selected from MDACC lung cancer tissue bank. The results (Supplementary Figure S1) showed that the survival time and distributions were very similar between the two cohorts. Also, there was no significant difference in gender and histology (Supplementary Table S1).

Classification method

Recursive Partitioning and Regression Trees (RPART) was used as the supervised classification method in this paper. Recursive partitioning creates a

decision tree (http://en.wikipedia.org/wiki/Decision_tree_learning) that strives to correctly classify members of the population based on several dichotomous dependent variables (http://en.wikipedia.org/wiki/Dependent_variable), and it is a widely used classification method in biomedical research [22,23,24,25]. Recursive partitioning is a nonparametric method and does not make distribution assumptions for the predictor variables. The algorithm itself is simple and intuitive. At each step, the recursive partitioning program determines for each variable (in this case for each of the NR genes) a cutoff point that best splits all of the individuals into low risk and high risk groups and selects the variable that performs best. Next, the process is repeated on each of the resulting subpopulations. The iteration will stop until either a subpopulation contains one class of individuals or the subpopulation is too small to subdivide. In this study, the response variable in the recursive partitioning model was the survival time, either overall survival or recurrence-free survival; the co-variables in the model are all NR genes. The program RPART (version 3.1), a freely available R package [50], was implemented to generate the decision tree. All parameters were used as the default values set in the package. The relative risk of each individual patient (relative to the overall population in the training data) was predicted from the tree model. The patients with predicted relative risk greater than one were considered as high-risk group, and otherwise as low-risk group. In our analysis, there was no gene selection step before model building and all the parameters used in the prediction model were predefined as the default value in R program; therefore, the testing data were not used for the model building

procedure, similar to a blinded testing procedure. In order to explore the roles of individual NRs or subsets of NRs in prediction models, we looked into the tree structure of prediction models and found that SHP expression was the only co-variable left in the prediction model built from the 30-patient MDACC data set. In order to see the prognosis ability of other NR genes, we removed SHP from the prediction model and rebuilt the classification tree. In this second analysis (without SHP), PR expression was found to be the only co-variable left in the prediction model. All tree structures and parameters used in the prediction model can be found in the supplemental SWEAVE report (Text S2).
