# Supplementary material for: Nuclear Receptor Expression Defines a Set of Prognostic Biomarkers for Lung Cancer
Source: PLoS Med. 2010 Dec 14;7(12):e1000378. doi: 10.1371/journal.pmed.1000378 (PMC3001894; doi:10.1371/journal.pmed.1000378)
Supplement: Text S2 — Sweave document. Nuclear receptor expression profiling defines a set of prognostic biomarkers for lung cancer. (0.59 MB PDF) [file pmed.1000378.s018.pdf]

Text S2: Sweave Document

Nuclear Receptor Expression Profiling Defines a Set of Prognostic Biomarkers for Lung Cancer

Yangsik Jeong, Yang Xie, Guanghua Xiao, Carmen Behrens, Luc Girard,
Ignacio I Wistuba, John D Minna & David J Mangelsdorf

```
> library(survival)
> library(rpart)
> library(survivalROC)
```

```
> pv.expr <- function(x, digits = 1) {
+     if (!x)
+         return(0)
+     exponent <- floor(log10(x))
+     base <- round(x/10^exponent, digits)
+     ifelse(x > 1e-04, paste("pv = ", base * (10^exponent), sep = ""),
+         paste("pv = ", base, "E", exponent, sep = ""))
+ }
```

Unsupervised cluster analysis of the MDACC dataset

```
> mda <- read.csv("MDA_data_Jan 24 2010.csv", row.names = 1)
> mda.pcr <- mda[, -(1:4)]
> mda.pcr[mda.pcr == 0] <- min(mda.pcr[mda.pcr != 0])
> mda[, -(1:4)] <- mda.pcr <- log2(mda.pcr)
```

```
> rgb.palette <- colorRampPalette(c("green", "black", "red"), space = "rgb")
> heatmap(t(mda.pcr), scale = "none", col = rgb.palette(13), margins = c(4,
+     4), cex.axis = 1)
```

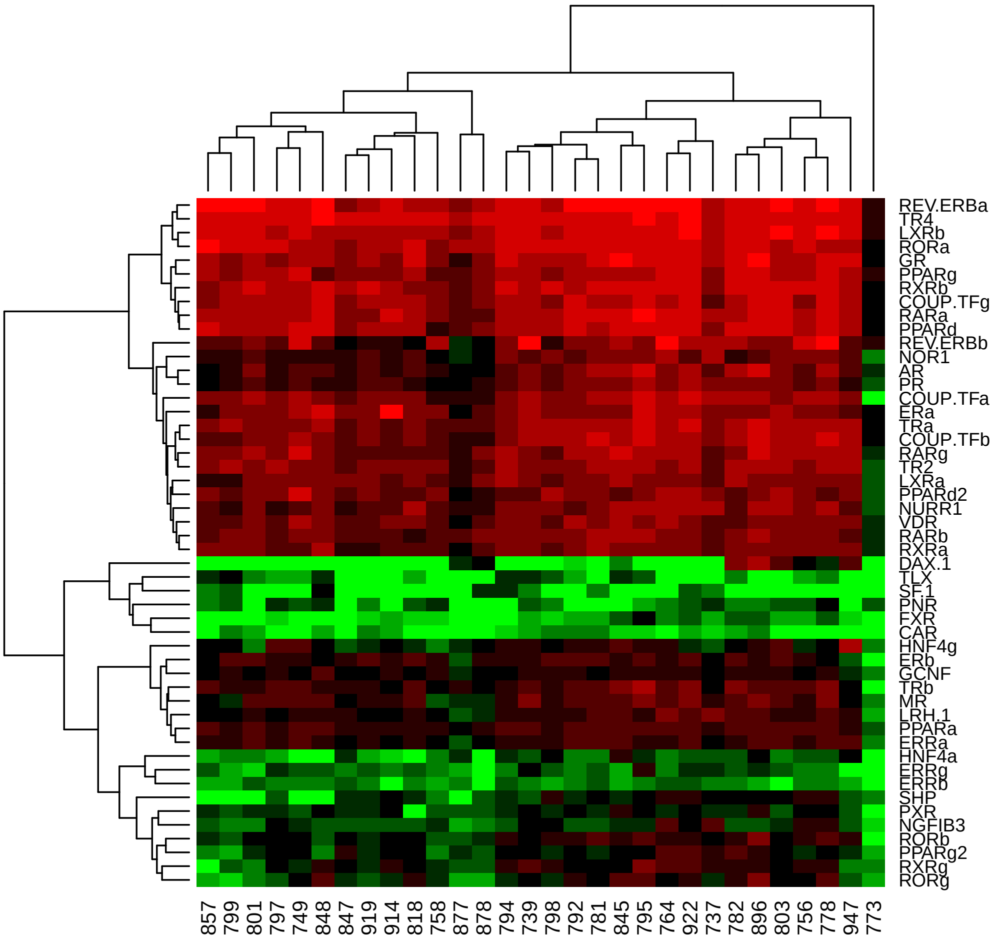

Figure 2A. Unsupervised cluster analysis of the 30 MDACC lung cancer patient cohort using the QPCR profile of the NR superfamily.

```
> cluster <- cutree(hclust(dist(mda.pcr)), k = 3)
> mda.clust <- data.frame(cluster, mda[, 1:4])[cluster != 3, ]
```

Note that one tissue sample (sample ID = 773) did not fall into either cluster and was treated as an outlier for the clustering analysis. Next, we tested whether the two major branches of the dendrogram were associated with both overall survival rates and disease recurrence rates.

```
> sf <- survfit(Surv(Survival_Time, Dead) ~ cluster, data = mda.clust)
> logrank <- survdiff(Surv(Survival_Time, Dead) ~ cluster, data = mda.clust)
> logrank

Call:
survdiff(formula = Surv(Survival_Time, Dead) ~ cluster, data = mda.clust)
```

```
           N Observed Expected (O-E)^2/E (O-E)^2/V
cluster=1 16        5    11.65       3.8      15.6
cluster=2 13       11     4.35      10.2      15.6

 Chisq= 15.6  on 1 degrees of freedom, p= 7.95e-05

> pv <- pchisq(logrank$chisq, 1, lower.tail = F)
> summary(coxph(Surv(Survival_Time, Dead) ~ cluster, data = mda.clust))

Call:
coxph(formula = Surv(Survival_Time, Dead) ~ cluster, data = mda.clust)

  n= 29

          coef exp(coef) se(coef)     z Pr(>|z|)
cluster 2.1332    8.4419   0.6168 3.458 0.000543 ***
---
Signif. codes:  0 Ś***Š 0.001 Ś**Š 0.01 Ś*Š 0.05 Ś.Š 0.1 Ś Š 1

        exp(coef) exp(-coef) lower .95 upper .95
cluster     8.442     0.1185      2.52     28.28

Rsquare= 0.383   (max possible= 0.958 )
Likelihood ratio test= 14  on 1 df,   p=0.0001829
Wald test            = 11.96  on 1 df,   p=0.0005432
Score (logrank) test = 15.57  on 1 df,   p=7.95e-05

> plot(sf, main = "", xlab = "Survival time (month)", ylab = "Survival",
+     cex.lab = 1.5, mark = c(1, 19), cex = 1, col = 1:2)
> text(60, 0.5, pv.expr(pv), cex = 1.5)
```

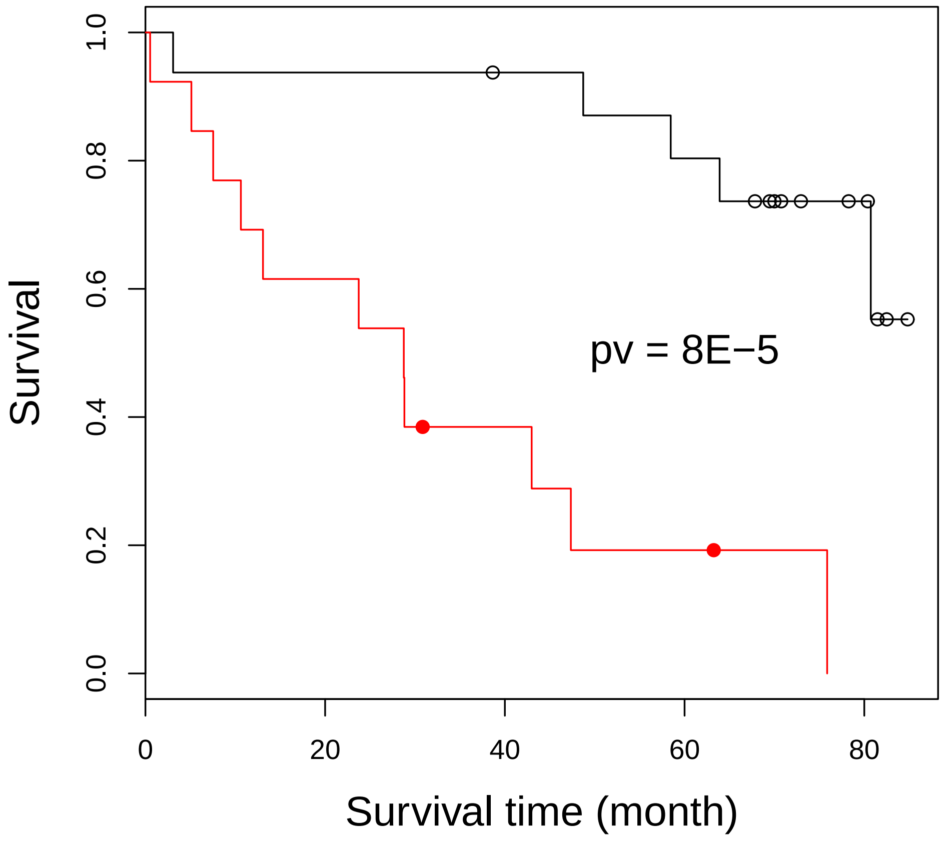

Figure 2B. Kaplan-Meier plot showing the association of the NR gene signature with overall patient survival.

```
> sf <- survfit(Surv(TOE, Progression) ~ cluster, data = mda.clust)
> logrank <- survdiff(Surv(TOE, Progression) ~ cluster, data = mda.clust)
> logrank

Call:
survdiff(formula = Surv(TOE, Progression) ~ cluster, data = mda.clust)

           N Observed Expected (O-E)^2/E (O-E)^2/V
cluster=1 16       10    15.64      2.04      7.84
cluster=2 13       12     6.36      5.01      7.84

 Chisq= 7.8  on 1 degrees of freedom, p= 0.00511

> pv <- pchisq(logrank$chisq, 1, lower.tail = F)
> summary(coxph(Surv(TOE, Progression) ~ cluster, data = mda.clust))
```

```
Call:
coxph(formula = Surv(TOE, Progression) ~ cluster, data = mda.clust)

  n= 29

          coef exp(coef) se(coef)     z Pr(>|z|)
cluster 1.2635    3.5377   0.4746 2.662  0.00776 **
---
Signif. codes:  0 Ś***Š 0.001 Ś**Š 0.01 Ś*Š 0.05 Ś.Š 0.1 Ś Š 1

        exp(coef) exp(-coef) lower .95 upper .95
cluster     3.538     0.2827     1.396     8.968

Rsquare= 0.222   (max possible= 0.986 )
Likelihood ratio test= 7.26  on 1 df,   p=0.007037
Wald test            = 7.09  on 1 df,   p=0.007765
Score (logrank) test = 7.89  on 1 df,   p=0.00498
```

```
> {
+     plot(sf, main = "", xlab = "Time to recurrence (month)",
+         ylab = "Recurrence free survival", cex.lab = 1.5, mark = c(1,
+             19), cex = 1, col = 1:2)
+     text(50, 0.9, pv.expr(pv), cex = 1.5)
+ }
```

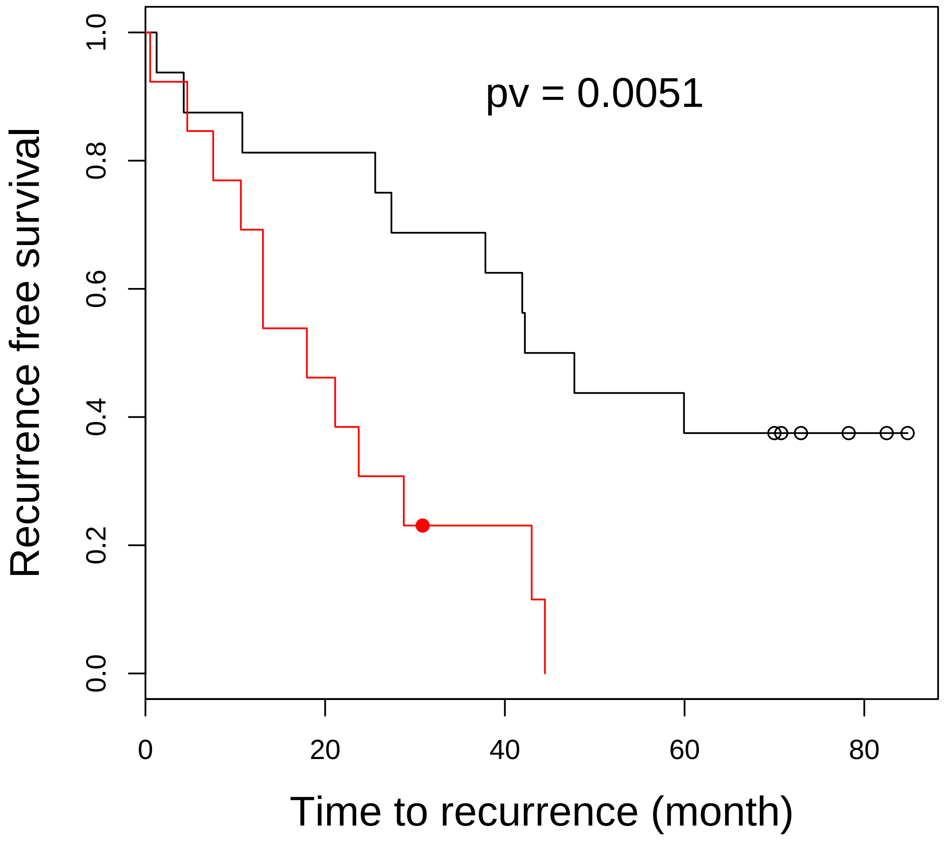

Figure 2C. Kaplan-Meier plot showing the association of the NR gene signature with disease recurrence.

Classification tree models for the MDACC dataset

```
> mda.surv <- mda[, -(3:4)]
> fit <- rpart(Surv(Survival_Time, Dead) ~ ., data = mda.surv)
> print(fit)

n= 30

node), split, n, deviance, yval
      * denotes terminal node

1) root 30 42.274540 1.0000000
  2) SHP>=-8.455706 13  6.053077 0.1735668 *
  3) SHP< -8.455706 17 12.545500 2.2736220 *
```

```
> res <- rep(0, 30)
> for (i in 1:30) {
+     fit <- rpart(Surv(Survival_Time, Dead) ~ ., data = mda.surv[-i,
+         ])
+     res[i] <- (predict(fit, newdat = mda.surv[i, ]) > 1)
+ }
> sf <- survfit(Surv(Survival_Time, Dead) ~ res, data = mda.surv)
> summary(coxph(Surv(Survival_Time, Dead) ~ res, data = mda.surv))

Call:
coxph(formula = Surv(Survival_Time, Dead) ~ res, data = mda.surv)

  n= 30

       coef exp(coef) se(coef)     z Pr(>|z|)
res  2.6149   13.6659   0.7626 3.429 0.000606 ***
---
Signif. codes:  0 Ś***Š 0.001 Ś**Š 0.01 Ś*Š 0.05 Ś.Š 0.1 Ś Š 1

    exp(coef) exp(-coef) lower .95 upper .95
res     13.67    0.07318     3.065     60.92

Rsquare= 0.477   (max possible= 0.963 )
Likelihood ratio test= 19.45  on 1 df,   p=1.031e-05
Wald test            = 11.76  on 1 df,   p=0.0006063
Score (logrank) test = 18.94  on 1 df,   p=1.35e-05

> logrank <- survdiff(Surv(Survival_Time, Dead) ~ res, data = mda.surv)
> logrank

Call:
survdiff(formula = Surv(Survival_Time, Dead) ~ res, data = mda.surv)

       N Observed Expected (O-E)^2/E (O-E)^2/V
res=0 14        2     10.5      6.88      18.9
res=1 16       15      6.5     11.11      18.9

 Chisq= 18.9  on 1 degrees of freedom, p= 1.35e-05

> pv <- pchisq(logrank$chisq, 1, lower.tail = F)

> plot(sf, conf.int = F, main = "MDACC LOOCV", xlab = "Survival time (month)",
+     ylab = "Survival", cex.lab = 1.2, mark = c(1, 19), cex = 1,
+     col = 1:2)
> text(50, 0.6, pv.expr(pv), cex = 1.5)
```

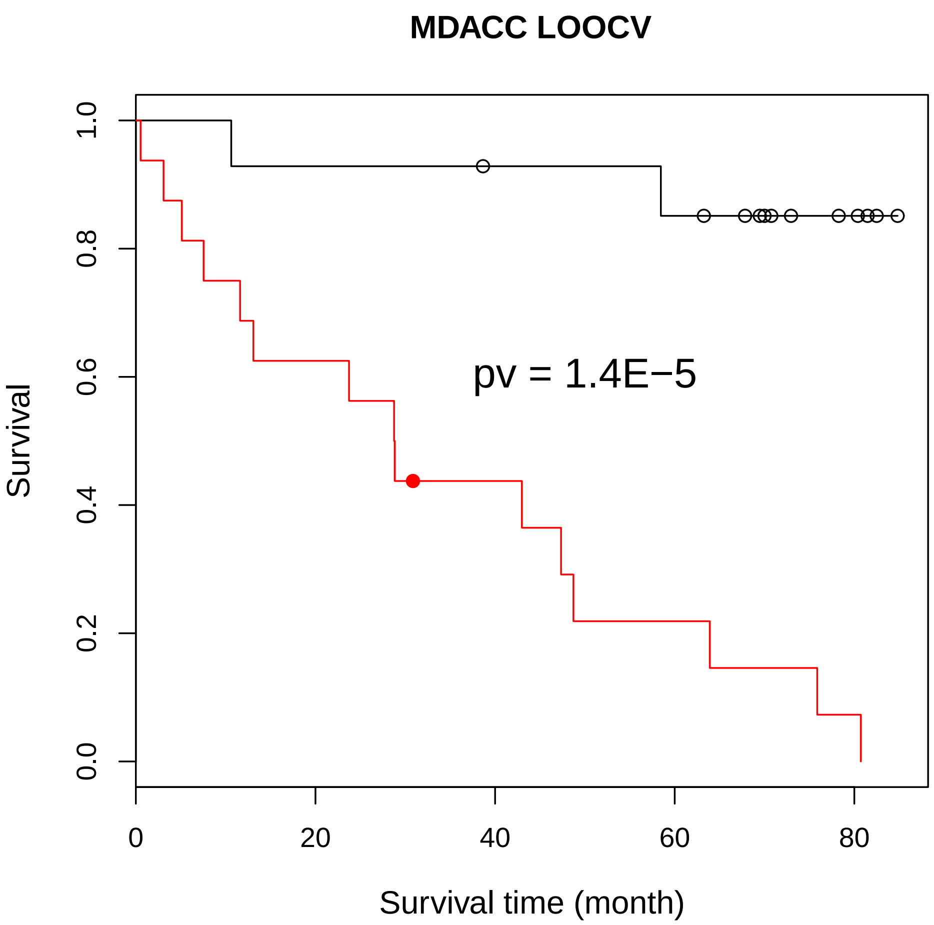

Figure 3A. Leave one out cross validation (LOOCV) of the recursive-partitioning tree model (RPART) for the 30-sample MDACC QPCR dataset using all 48 NRs.

Classification tree models for the consortium dataset

```
> Consortium <- read.csv("Consortium_data.csv", row.names = 1)

> dat.train <- Consortium[Consortium$TESTTYPE == "Train", c(1,
+     2, 10:57)]
> dat.test <- Consortium[Consortium$TESTTYPE == "Test", c(1, 2,
+     10:57)]
> fit <- rpart(Surv(month, death) ~ ., data = dat.train)
> print(fit)
```

```
n=254 (1 observation deleted due to missingness)

node), split, n, deviance, yval
```

```
      * denotes terminal node

   1) root 254 383.721300 1.0000000
     2) SF.1>=5.035 238 355.235900 0.9321384
       4) COUP.TFb>=6.17875 202 291.440300 0.8316414
         8) PPARd< 6.396667 190 264.976500 0.7728499
          16) COUP.TFb< 7.47875 178 244.205400 0.7107610
            32) PPARd< 5.698333 7   1.733478 0.1332612 *
            33) PPARd>=5.698333 171 234.749600 0.7479395
              66) DAX.1< 4.6775 62  71.571530 0.4847427
               132) TRb< 6.335 9   1.758713 0.1206434 *
               133) TRb>=6.335 53  64.098200 0.5645271
                 266) ERRa< 7.2075 46  48.769070 0.4704948
                   532) COUP.TFg>=6.725 13  11.104640 0.1410123 *
                   533) COUP.TFg< 6.725 33  29.811300 0.6617683
                    1066) RXRg>=5.255 10   5.824201 0.2138250 *
                    1067) RXRg< 5.255 23  17.427060 0.9020286 *
                 267) ERRa>=7.2075 7  10.083650 1.4280350 *
              67) DAX.1>=4.6775 109 154.239700 0.9353668
               134) NOR1< 5.808333 41  64.483780 0.5934419
                 268) NURR1< 5.873333 15  11.672550 0.2041338 *
                 269) NURR1>=5.873333 26  43.908780 0.8997544
                   538) PR>=4.275 17  26.493760 0.5361375 *
                   539) PR< 4.275 9   6.248823 2.3703860 *
               135) NOR1>=5.808333 68  81.456440 1.2175480
                 270) MR>=5.945 58  68.849110 1.0750540
                   540) ERa>=5.621667 7   4.285743 0.3296640 *
                   541) ERa< 5.621667 51  58.806370 1.2095250
                    1082) PNR< 4.7225 16  26.455710 0.6444469 *
                    1083) PNR>=4.7225 35  26.466970 1.5237510
                      2166) COUP.TFb< 6.83625 26  12.252950 1.2556770 *
                      2167) COUP.TFb>=6.83625 9   8.916298 2.8217610 *
                 271) MR< 5.945 10   6.484173 2.5454740 *
          17) COUP.TFb>=7.47875 12   9.302349 2.1612190 *
         9) PPARd>=6.396667 12  13.595450 2.5960540 *
       5) COUP.TFb< 6.17875 36  52.937150 1.6650440
        10) ERRa< 7.0875 23  32.112400 1.2075800
          20) COUP.TFg>=6.63 12  12.389740 0.7048566 *
          21) COUP.TFg< 6.63 11  12.325520 2.2287740 *
        11) ERRa>=7.0875 13  12.719170 3.1216850 *
     3) SF.1< 5.035 16  13.967780 2.6992260 *
```

```
> group <- ifelse(predict(fit, newdat = dat.test) > 1, "High",
+     " Low")
> sf <- survfit(Surv(month, death) ~ group, data = dat.test)
> summary(coxph(Surv(month, death) ~ group, data = dat.test))
```

```
Call:
coxph(formula = Surv(month, death) ~ group, data = dat.test)
```

```
  n=186 (1 observation deleted due to missingness)

           coef exp(coef) se(coef)     z Pr(>|z|)
groupHigh 0.7124    2.0388   0.3061 2.327   0.0200 *
---
Signif. codes:  0 Ś***Š 0.001 Ś**Š 0.01 Ś*Š 0.05 Ś.Š 0.1 Ś Š 1

          exp(coef) exp(-coef) lower .95 upper .95
groupHigh     2.039     0.4905     1.119     3.715

Rsquare= 0.033   (max possible= 0.975 )
Likelihood ratio test= 6.25  on 1 df,   p=0.01242
Wald test            = 5.42  on 1 df,   p=0.01995
Score (logrank) test = 5.65  on 1 df,   p=0.01747

> logrank <- survdiff(Surv(month, death) ~ group, data = dat.test)
> logrank

Call:
survdiff(formula = Surv(month, death) ~ group, data = dat.test)

n=186, 1 observation deleted due to missingness.

             N Observed Expected (O-E)^2/E (O-E)^2/V
group= Low  51       13     22.3      3.89      5.64
group=High 135       61     51.7      1.68      5.64

 Chisq= 5.6  on 1 degrees of freedom, p= 0.0175

> pv <- pchisq(logrank$chisq, 1, lower.tail = F)

> dat.test <- Consortium[Consortium$TESTTYPE == "Test", ]
> summary(coxph(Surv(month, death) ~ group + stage + GENDER + AGE_AT_DIAGNOSIS +
+     ADJUVANT_CHEMO + ADJUVANT_RT, data = dat.test))

Call:
coxph(formula = Surv(month, death) ~ group + stage + GENDER +
    AGE_AT_DIAGNOSIS + ADJUVANT_CHEMO + ADJUVANT_RT, data = dat.test)

  n=152 (35 observations deleted due to missingness)

                    coef exp(coef) se(coef)     z Pr(>|z|)
groupHigh        0.68416   1.98210  0.32769 2.088 0.036815 *
stage            1.01596   2.76202  0.28999 3.503 0.000459 ***
GENDER           0.62878   1.87531  0.26840 2.343 0.019147 *
AGE_AT_DIAGNOSIS 0.01818   1.01835  0.01485 1.224 0.220862
ADJUVANT_CHEMO   0.70510   2.02405  0.29277 2.408 0.016024 *
ADJUVANT_RT      0.39077   1.47812  0.30839 1.267 0.205108
```

```
---
Signif. codes:  0 Ś***Š 0.001 Ś**Š 0.01 Ś*Š 0.05 Ś.Š 0.1 Ś Š 1

                 exp(coef) exp(-coef) lower .95 upper .95
groupHigh            1.982     0.5045    1.0428     3.768
stage                2.762     0.3621    1.5645     4.876
GENDER               1.875     0.5332    1.1082     3.174
AGE_AT_DIAGNOSIS     1.018     0.9820    0.9891     1.048
ADJUVANT_CHEMO       2.024     0.4941    1.1403     3.593
ADJUVANT_RT          1.478     0.6765    0.8076     2.705

Rsquare= 0.251   (max possible= 0.97 )
Likelihood ratio test= 43.98  on 6 df,   p=7.475e-08
Wald test            = 42.55  on 6 df,   p=1.432e-07
Score (logrank) test = 48.04  on 6 df,   p=1.160e-08
```

```
> {
+     plot(sf, conf.int = F, main = "Consortium Training to Testing",
+         xlab = "Survival time (Month)", ylab = "Survival", cex.lab = 1.2,
+         mark = c(1, 19), col = 1:2, cex = 1, lty = 1, lwd = 2)
+     text(100, 0.9, pv.expr(pv), cex = 1.5)
+ }
```

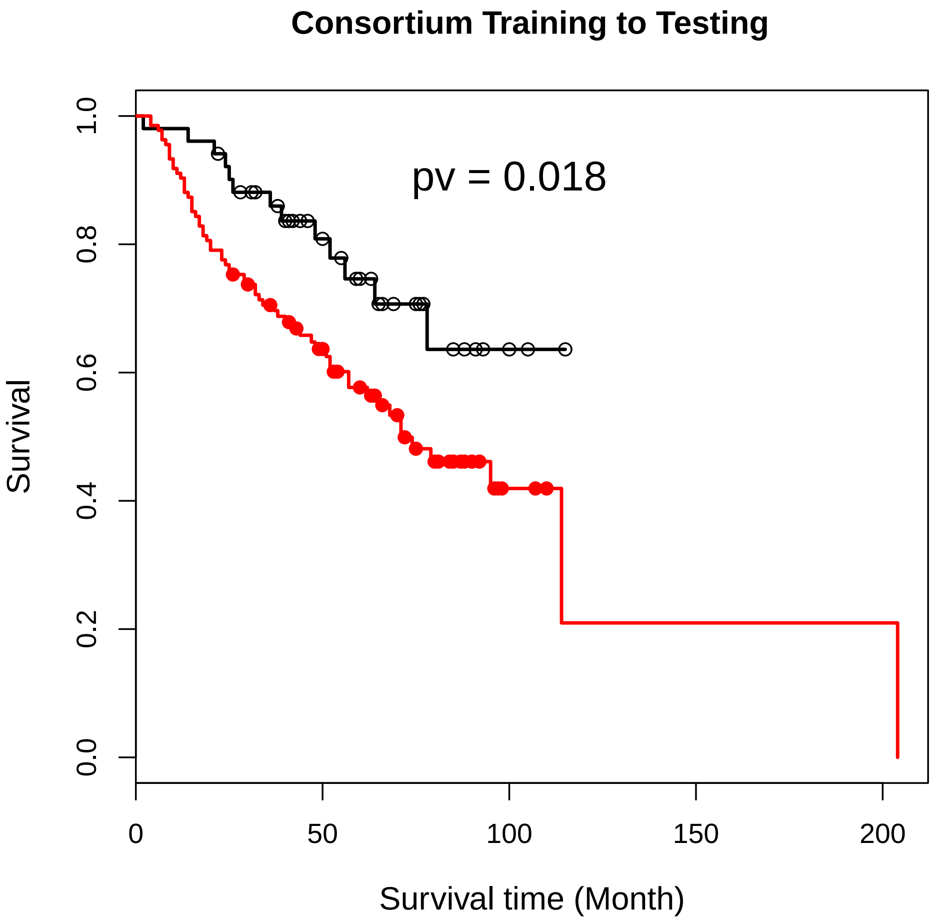

Figure 3D. Independent validation of the NR gene signature in the 442-sample cohort multi-institute consortium using RPART analysis. The microarray datasets were divided into two groups, one for the training and the other for the testing cohort.

```
> clin.train <- Consortium[Consortium$TESTTYPE == "Train", c(1,
+     2, 5:9)]
> clin.test <- Consortium[Consortium$TESTTYPE == "Test", c(1, 2,
+     5:9)]
> fit <- rpart(Surv(month, death) ~ ., data = clin.train)
> print(fit)

n=254 (1 observation deleted due to missingness)

node), split, n, deviance, yval
      * denotes terminal node

 1) root 254 383.72130 1.0000000
```

```
   2) stage< 1.5 156 202.73770 0.6682930
     4) AGE_AT_DIAGNOSIS< 60.5 44  54.22007 0.4087002 *
     5) AGE_AT_DIAGNOSIS>=60.5 112 141.08380 0.8089726 *
   3) stage>=1.5 98 137.29060 1.8965040
     6) AGE_AT_DIAGNOSIS< 74.5 80 108.28500 1.6995780
      12) AGE_AT_DIAGNOSIS< 65.5 49  59.73886 1.4721950
        24) AGE_AT_DIAGNOSIS>=62.5 10  14.32239 0.8072023 *
        25) AGE_AT_DIAGNOSIS< 62.5 39  41.29811 1.7437340 *
      13) AGE_AT_DIAGNOSIS>=65.5 31  46.28752 2.0954350
        26) AGE_AT_DIAGNOSIS>=70.5 15  28.37785 1.3484000 *
        27) AGE_AT_DIAGNOSIS< 70.5 16  11.20137 3.2701420 *
     7) AGE_AT_DIAGNOSIS>=74.5 18  22.72920 3.2184120 *
```

```
> group <- ifelse(predict(fit, newdat = clin.test) > 1, "High",
+     " Low")
> sf <- survfit(Surv(month, death) ~ group, data = clin.test)
> logrank <- survdiff(Surv(month, death) ~ group, data = clin.test)
> logrank
```

```
Call:
survdiff(formula = Surv(month, death) ~ group, data = clin.test)

n=186, 1 observation deleted due to missingness.

             N Observed Expected (O-E)^2/E (O-E)^2/V
group= Low 128       39     55.4      4.83      19.5
group=High  58       35     18.6     14.34      19.5

 Chisq= 19.5  on 1 degrees of freedom, p= 1.02e-05
```

```
> pv <- pchisq(logrank$chisq, 1, lower.tail = F)
> summary(coxph(Surv(month, death) ~ group, data = clin.test))
```

```
Call:
coxph(formula = Surv(month, death) ~ group, data = clin.test)

  n=186 (1 observation deleted due to missingness)

            coef exp(coef) se(coef)     z Pr(>|z|)
groupHigh 0.9972    2.7108   0.2349 4.245 2.18e-05 ***
---
Signif. codes:  0 Ś***Š 0.001 Ś**Š 0.01 Ś*Š 0.05 Ś.Š 0.1 Ś Š 1

          exp(coef) exp(-coef) lower .95 upper .95
groupHigh     2.711     0.3689     1.711     4.296

Rsquare= 0.088   (max possible= 0.975 )
Likelihood ratio test= 17.11  on 1 df,   p=3.524e-05
Wald test            = 18.02  on 1 df,   p=2.183e-05
Score (logrank) test = 19.54  on 1 df,   p=9.828e-06
```

```
> plot(sf, conf.int = F, main = "Clinical Only", xlab = "Survival time (Month)",
+     ylab = "Survival", cex.lab = 1.2, cex = 1, mark = c(1, 19),
+     col = 1:2, lwd = 2)
> text(100, 0.9, pv.expr(pv), cex = 1.5)
```

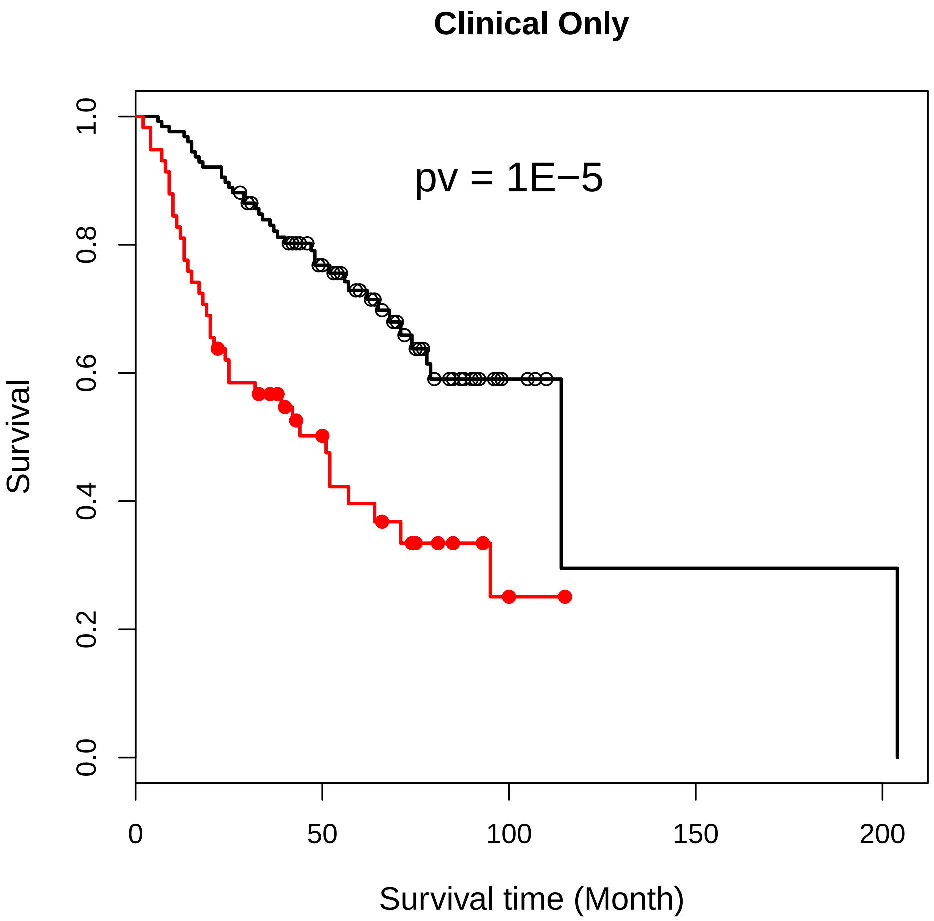

Figure S7A. The analysis for survival time was performed for clinical variables in the absence of NR expression.

```
> nr.train <- Consortium[Consortium$TESTTYPE == "Train", c(10:57)]
> pca.train <- prcomp(nr.train)
> nr.pc.train <- as.matrix(nr.train) %*% pca.train$rotation
> nr.test <- Consortium[Consortium$TESTTYPE == "Test", c(10:57)]
> nr.pc.test <- as.matrix(nr.test) %*% pca.train$rotation
> clin.train <- Consortium[Consortium$TESTTYPE == "Train", c(1,
+     2, 5:9)]
> clin.train$nr1 <- nr.pc.train[, 1]
> clin.train$nr2 <- nr.pc.train[, 2]
```

```
> clin.test <- Consortium[Consortium$TESTTYPE == "Test", c(1, 2,
+     5:9)]
> clin.test$nr1 <- nr.pc.test[, 1]
> clin.test$nr2 <- nr.pc.test[, 2]
> fit <- rpart(Surv(month, death) ~ ., data = clin.train)
> print(fit)

n=254 (1 observation deleted due to missingness)

node), split, n, deviance, yval
      * denotes terminal node

 1) root 254 383.721300 1.0000000
   2) stage< 1.5 156 202.737700 0.6682930
     4) AGE_AT_DIAGNOSIS< 60.5 44  54.220070 0.4087002 *
     5) AGE_AT_DIAGNOSIS>=60.5 112 141.083800 0.8089726 *
   3) stage>=1.5 98 137.290600 1.8965040
     6) AGE_AT_DIAGNOSIS< 74.5 80 108.285000 1.6995780
      12) nr2>=8.322201 68  99.069870 1.5448400
        24) AGE_AT_DIAGNOSIS< 65.5 41  51.818410 1.2702110 *
        25) AGE_AT_DIAGNOSIS>=65.5 27  43.662770 2.0734760
          50) AGE_AT_DIAGNOSIS>=70.5 13  27.925630 1.3459110 *
          51) AGE_AT_DIAGNOSIS< 70.5 14  10.693820 3.0711610 *
      13) nr2< 8.322201 12   5.180607 2.7309730 *
     7) AGE_AT_DIAGNOSIS>=74.5 18  22.729200 3.2184120 *

> group <- ifelse(predict(fit, newdat = clin.test) > 1, "High",
+     " Low")
> sf <- survfit(Surv(month, death) ~ group, data = clin.test)
> logrank <- survdiff(Surv(month, death) ~ group, data = clin.test)
> logrank

Call:
survdiff(formula = Surv(month, death) ~ group, data = clin.test)

n=186, 1 observation deleted due to missingness.

             N Observed Expected (O-E)^2/E (O-E)^2/V
group= Low 119       32     52.0       7.7      26.7
group=High  67       42     22.0      18.2      26.7

 Chisq= 26.7  on 1 degrees of freedom, p= 2.43e-07

> pv <- pchisq(logrank$chisq, 1, lower.tail = F)
> summary(coxph(Surv(month, death) ~ group, data = clin.test))

Call:
coxph(formula = Surv(month, death) ~ group, data = clin.test)
```

```
  n=186 (1 observation deleted due to missingness)

            coef exp(coef) se(coef)     z Pr(>|z|)
groupHigh 1.1658    3.2086   0.2382 4.895 9.82e-07 ***
---
Signif. codes:  0 Ś***Š 0.001 Ś**Š 0.01 Ś*Š 0.05 Ś.Š 0.1 Ś Š 1

          exp(coef) exp(-coef) lower .95 upper .95
groupHigh     3.209     0.3117     2.012     5.117

Rsquare= 0.121   (max possible= 0.975 )
Likelihood ratio test= 24.01  on 1 df,   p=9.561e-07
Wald test            = 23.96  on 1 df,   p=9.82e-07
Score (logrank) test = 26.7  on 1 df,   p=2.373e-07
```

```
> plot(sf, conf.int = F, main = "Clinical + NR", xlab = "Survival time (Month)",
+     ylab = "Survival", cex.lab = 1.2, cex = 1, mark = c(1, 19),
+     lwd = 2, col = 1:2)
> text(100, 0.9, pv.expr(pv), cex = 1.5)
```

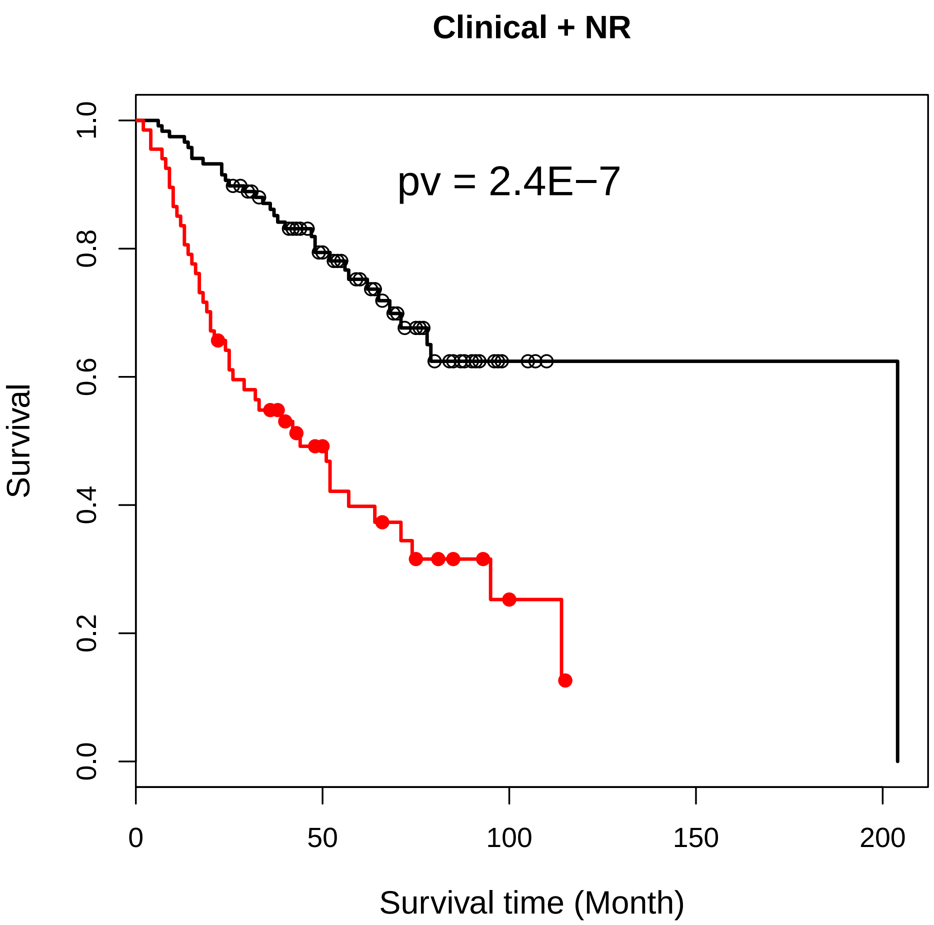

Figure S7B. The analysis for survival time was performed for clinical variables in the presence of NR expression.

Prediction across MDACC and Consortium

```
> Consortium.expr <- Consortium[, 10:57]
> Consortium.expr <- scale(Consortium.expr)
> mda.surv <- mda[, -(3:4)]
> mda.surv[, -(1:2)] <- scale(mda.surv[, -(1:2)])
> common.gene <- intersect(colnames(mda.surv)[-(1:2)], colnames(Consortium.expr))
> mda.data <- data.frame(type = "mda", Stage = NA, mda.surv[, 1:2],
+     mda.surv[, common.gene])
> Consortium.data <- data.frame(type = "Consortium", Stage = Consortium$stage,
+     Dead = Consortium$death, Survival_Time = Consortium$month,
+     Consortium.expr[, common.gene])
> combined <- data.frame(rbind(mda.data, Consortium.data))
```

```
> data.train <- combined[combined$type == "mda", ]
> data.test <- combined[combined$type == "Consortium", ]
> fit <- rpart(Surv(Survival_Time, Dead) ~ ., data = data.train)
> print(fit)

n= 30

node), split, n, deviance, yval
      * denotes terminal node

1) root 30 42.274540 1.0000000
  2) SHP>=0.4814558 13  6.053077 0.1735668 *
  3) SHP< 0.4814558 17 12.545500 2.2736220 *
```

The classification tree structure revealed that SHP expression was identified as the only covariable left in the final RPART prediction model built from the 30-patient MDACC dataset. In other words, the prognosis performance of the 48 NR gene signature (shown in Figures 3A and 3B) is the same as using SHP expression alone to build the models.

```
> group <- ifelse(predict(fit, newdat = data.test) > 1, "High",
+     " Low")
> sf <- survfit(Surv(Survival_Time, Dead) ~ group, data = data.test)
> summary(coxph(Surv(Survival_Time, Dead) ~ group, data = data.test))

Call:
coxph(formula = Surv(Survival_Time, Dead) ~ group, data = data.test)

  n=440 (2 observations deleted due to missingness)

            coef exp(coef) se(coef)     z Pr(>|z|)
groupHigh 0.4777    1.6123   0.1813 2.635  0.00843 **
---
Signif. codes:  0 Ś***Š 0.001 Ś**Š 0.01 Ś*Š 0.05 Ś.Š 0.1 Ś Š 1

          exp(coef) exp(-coef) lower .95 upper .95
groupHigh     1.612     0.6202      1.13     2.300

Rsquare= 0.017   (max possible= 0.997 )
Likelihood ratio test= 7.73  on 1 df,   p=0.005442
Wald test            = 6.94  on 1 df,   p=0.008426
Score (logrank) test = 7.07  on 1 df,   p=0.007824

> logrank <- survdiff(Surv(Survival_Time, Dead) ~ group, data = data.test)
> logrank

Call:
survdiff(formula = Surv(Survival_Time, Dead) ~ group, data = data.test)

n=440, 2 observations deleted due to missingness.
```

```
            N Observed Expected (O-E)^2/E (O-E)^2/V
group= Low  92       36       53      5.46      7.08
group=High 348      200      183      1.58      7.08

 Chisq= 7.1  on 1 degrees of freedom, p= 0.00779
```

```
> pv <- pchisq(logrank$chisq, 1, lower.tail = F)
> plot(sf, conf.int = F, main = "MDACC to consortium", xlab = "Survival time (Month)",
+     ylab = "Survival", cex.lab = 1.2, mark = c(1, 19), cex = 1,
+     col = 1:2, , lwd = 2)
> text(140, 0.9, pv.expr(pv), cex = 1.5)
```

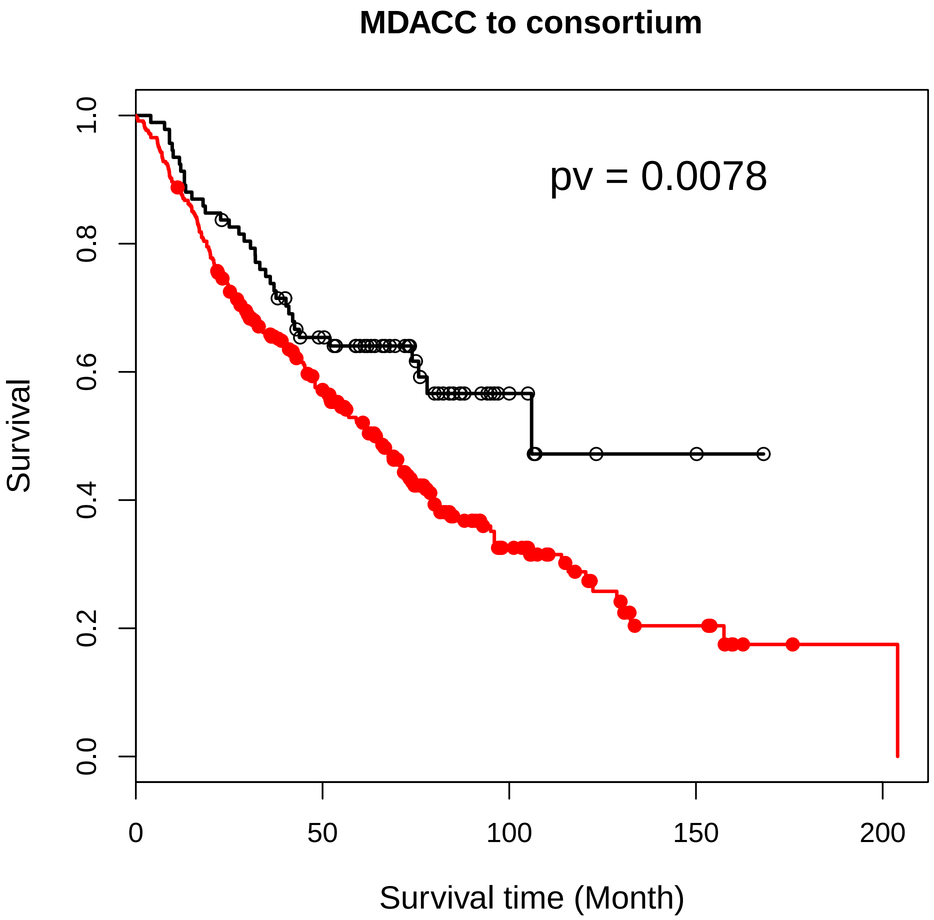

Figure 3B. Independent validation of the 48 NR gene-expression signature between the MDACC cohort and consortium cohort. The MDACC cohort training set was tested in the consortium cohort.

```
> data.train <- combined[combined$type == "Consortium", -2]
> data.test <- combined[combined$type == "mda", -2]
```

```
> fit <- rpart(Surv(Survival_Time, Dead) ~ ., data = data.train)
> print(fit)

n=440 (2 observations deleted due to missingness)

node), split, n, deviance, yval
      * denotes terminal node

   1) root 440 650.960700 1.0000000
     2) SF.1>=-1.600797 422 612.349500 0.9473605
       4) PPARd< 1.546829 393 557.598200 0.8889341
         8) RORa>=-1.110901 353 488.590600 0.8127724
          16) RARa>=-0.8263496 282 357.962100 0.7005085
            32) RARg< 0.7062089 206 236.112100 0.5957128
              64) NURR1< -1.126548 22   7.912262 0.1177802 *
              65) NURR1>=-1.126548 184 215.220500 0.6709268
               130) PXR>=1.269161 9   1.729126 0.1354368 *
               131) PXR< 1.269161 175 206.439800 0.7109939
                 262) TR2>=1.257944 11   4.844043 0.1918291 *
                 263) TR2< 1.257944 164 194.013000 0.7694938
                   526) LXRa>=-0.8594324 134 144.483400 0.6588147 *
                   527) LXRa< -0.8594324 30  42.461040 1.3332290
                    1054) ERa>=0.1125855 13  13.110410 0.6988573 *
                    1055) ERa< 0.1125855 17  21.932320 2.1376250 *
            33) RARg>=0.7062089 76 113.805200 1.0449810
              66) GR>=0.1164013 21  28.988230 0.4769354
               132) NGFIB3>=0.2269965 9   1.775888 0.1120559 *
               133) NGFIB3< 0.2269965 12  18.553220 1.0365880 *
              67) GR< 0.1164013 55  76.323680 1.3577180
               134) ERa>=1.44486 7   3.714183 0.2946938 *
               135) ERa< 1.44486 48  61.587110 1.6885060
                 270) FXR>=1.1885 7   7.610207 0.3961379 *
                 271) FXR< 1.1885 41  44.954860 2.0384140 *
          17) RARa< -0.8263496 71 118.121600 1.2993340
            34) SHP>=0.9231351 12  15.193210 0.4609037 *
            35) SHP< 0.9231351 59  95.018530 1.5203210
              70) DAX.1< -0.4961372 17  22.775660 0.6645850 *
              71) DAX.1>=-0.4961372 42  59.723230 2.1019450
               142) LXRb>=-0.3823468 15  17.205720 1.1617770 *
               143) LXRb< -0.3823468 27  32.151590 3.1673350 *
         9) RORa< -1.110901 40  54.464440 1.8193850
          18) AR>=-0.4973712 24  24.203220 1.1693540 *
          19) AR< -0.4973712 16  18.400040 3.6731330 *
       5) PPARd>=1.546829 29  40.620470 2.1935050
        10) PPARg< 0.3242693 19  22.356910 1.4967360 *
        11) PPARg>=0.3242693 10  11.316950 3.9286630 *
     3) SF.1< -1.600797 18  19.521260 3.1164310 *

> group <- ifelse(predict(fit, newdat = data.test) > 1, "High",
```

```
+      " Low")
> table(group)

group
 Low High
  21    9

> sf <- survfit(Surv(Survival_Time, Dead) ~ group, data = data.test)
> summary(coxph(Surv(Survival_Time, Dead) ~ group, data = data.test))

Call:
coxph(formula = Surv(Survival_Time, Dead) ~ group, data = data.test)

  n= 30

            coef exp(coef) se(coef)    z Pr(>|z|)
groupHigh 1.0041    2.7293   0.5516 1.82   0.0687 .
---
Signif. codes:  0 Ś***Š 0.001 Ś**Š 0.01 Ś*Š 0.05 Ś.Š 0.1 Ś Š 1

          exp(coef) exp(-coef) lower .95 upper .95
groupHigh     2.729     0.3664    0.9259     8.045

Rsquare= 0.097   (max possible= 0.963 )
Likelihood ratio test= 3.06  on 1 df,   p=0.08003
Wald test            = 3.31  on 1 df,   p=0.0687
Score (logrank) test = 3.57  on 1 df,   p=0.059

> logrank <- survdiff(Surv(Survival_Time, Dead) ~ group, data = data.test)
> logrank

Call:
survdiff(formula = Surv(Survival_Time, Dead) ~ group, data = data.test)

            N Observed Expected (O-E)^2/E (O-E)^2/V
group= Low 21       11    13.89     0.602      3.57
group=High  9        6     3.11     2.688      3.57

 Chisq= 3.6  on 1 degrees of freedom, p= 0.059

> pv <- pchisq(logrank$chisq, 1, lower.tail = F)

> plot(sf, conf.int = F, main = "Consortium to MDACC", xlab = "Survival time (Month)",
+     ylab = "Survival", cex.lab = 1.2, mark = c(1, 19), col = 1:2,
+     cex = 1, lwd = 2)
> text(20, 0.2, pv.expr(pv), cex = 1.5)
```

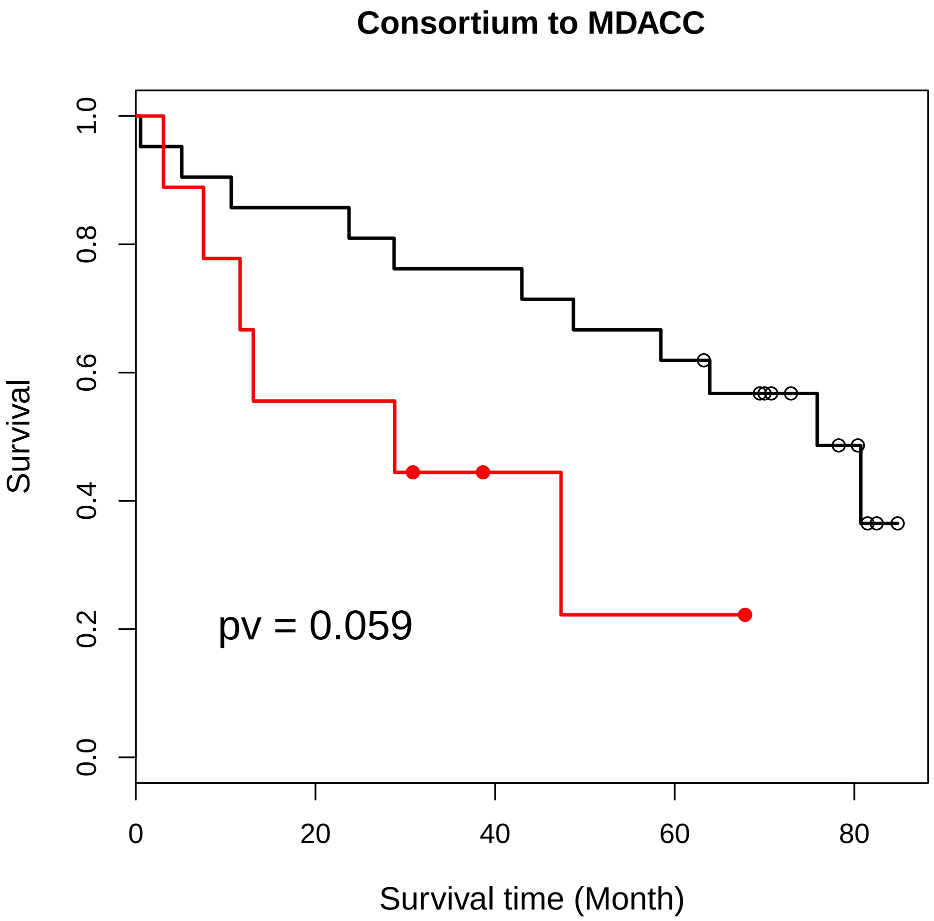

Figure 3C. Independent validation of the 48 NR gene-expression signature between the MDACC cohort and consortium cohort. The prediction model was build in the consortium cohort, and then validated in the MDACC cohort.

Refinement of the NR signature

Next, we removed SHP from the MDACC dataset in order to test the effect of other NR genes as biomarkers.

```
> ind.PR <- which(colnames(mda.surv) == "SHP")
> fit <- rpart(Surv(Survival_Time, Dead) ~ ., data = mda.surv[,
+     -ind.PR])
> print(fit)
```

```
n= 30

node), split, n, deviance, yval
```

```
      * denotes terminal node

1) root 30 42.274540 1.0000000
  2) PR>=0.04657526 17 12.407420 0.3528263 *
  3) PR< 0.04657526 13  8.020689 2.8993190 *
```

The classification tree structure revealed that when the prediction model excluded SHP, PR was now the single gene signature used.

```
> res <- rep(0, 30)
> for (i in 1:30) {
+     fit <- rpart(Surv(Survival_Time, Dead) ~ ., data = mda.surv[-i,
+         -ind.PR])
+     res[i] <- (predict(fit, newdat = mda.surv[i, ]) > 1)
+ }
> summary(coxph(Surv(Survival_Time, Dead) ~ res, data = mda.surv))

Call:
coxph(formula = Surv(Survival_Time, Dead) ~ res, data = mda.surv)

  n= 30

      coef exp(coef) se(coef)     z Pr(>|z|)
res 2.2593    9.5767   0.5934 3.808 0.000140 ***
---
Signif. codes:  0 Ś***Š 0.001 Ś**Š 0.01 Ś*Š 0.05 Ś.Š 0.1 Ś Š 1

    exp(coef) exp(-coef) lower .95 upper .95
res     9.577     0.1044     2.993     30.64

Rsquare= 0.44   (max possible= 0.963 )
Likelihood ratio test= 17.4  on 1 df,   p=3.027e-05
Wald test            = 14.5  on 1 df,   p=0.0001403
Score (logrank) test = 20.33  on 1 df,   p=6.52e-06

> sf <- survfit(Surv(Survival_Time, Dead) ~ res, data = mda.surv)
> logrank <- survdiff(Surv(Survival_Time, Dead) ~ res, data = mda.surv)
> logrank

Call:
survdiff(formula = Surv(Survival_Time, Dead) ~ res, data = mda.surv)

       N Observed Expected (O-E)^2/E (O-E)^2/V
res=0 17        5    12.75      4.71      20.3
res=1 13       12     4.25     14.15      20.3

 Chisq= 20.3  on 1 degrees of freedom, p= 6.52e-06

> pv <- pchisq(logrank$chisq, 1, lower.tail = F)
```

```
> plot(sf, conf.int = F, main = "MDACC LOOCV without SHP", xlab = "Survival time (month)",
+     ylab = "Survival", cex.lab = 1.2, mark = c(1, 19), lwd = 2,
+     cex = 1, col = 1:2)
> text(60, 0.6, pv.expr(pv), cex = 1.5)
```

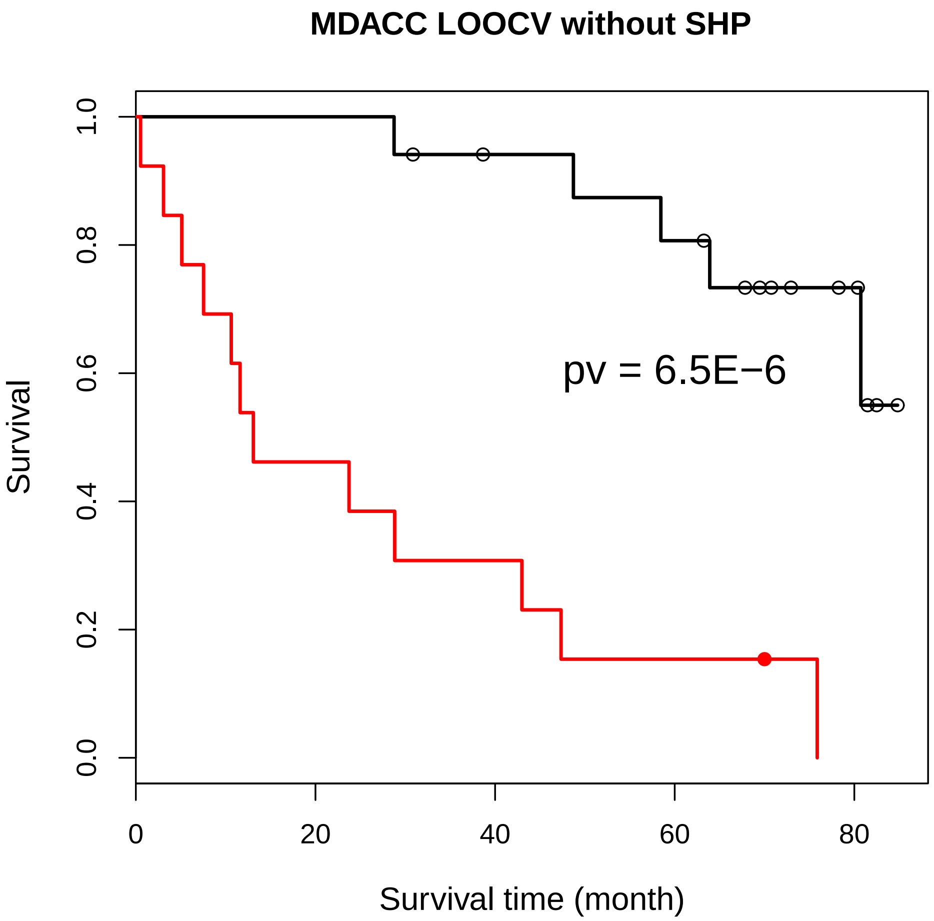

Figure 4A. The MDACC cohort was tested using LOOCV. For this analysis, mRNA expression values for SHP were removed from the dataset in order to test the effect of other NR genes as biomarkers.

```
> data.train <- combined[combined$type == "mda", colnames(combined) !=
+     "SHP"]
> data.test <- combined[combined$type == "Consortium", ]
> fit <- rpart(Surv(Survival_Time, Dead) ~ ., data = data.train)
> print(fit)

n= 30

node), split, n, deviance, yval
```

```
      * denotes terminal node

1) root 30 42.274540 1.0000000
  2) PR>=0.04657526 17 12.407420 0.3528263 *
  3) PR< 0.04657526 13  8.020689 2.8993190 *

> group <- ifelse(predict(fit, newdat = data.test) > 1, "High",
+     " Low")
> sf <- survfit(Surv(Survival_Time, Dead) ~ group, data = data.test)
> summary(coxph(Surv(Survival_Time, Dead) ~ group, data = data.test))

Call:
coxph(formula = Surv(Survival_Time, Dead) ~ group, data = data.test)

  n=440 (2 observations deleted due to missingness)

            coef exp(coef) se(coef)     z Pr(>|z|)
groupHigh 0.3774    1.4584   0.1347 2.801  0.00509 **
---
Signif. codes:  0 Ś***Š 0.001 Ś**Š 0.01 Ś*Š 0.05 Ś.Š 0.1 Ś Š 1

          exp(coef) exp(-coef) lower .95 upper .95
groupHigh     1.458     0.6857      1.12     1.899

Rsquare= 0.018   (max possible= 0.997 )
Likelihood ratio test= 8.03  on 1 df,   p=0.004595
Wald test            = 7.85  on 1 df,   p=0.005088
Score (logrank) test = 7.94  on 1 df,   p=0.004836

> logrank <- survdiff(Surv(Survival_Time, Dead) ~ group, data = data.test)
> logrank

Call:
survdiff(formula = Surv(Survival_Time, Dead) ~ group, data = data.test)

n=440, 2 observations deleted due to missingness.

             N Observed Expected (O-E)^2/E (O-E)^2/V
group= Low 209       91      112      4.11      7.97
group=High 231      145      124      3.74      7.97

 Chisq= 8  on 1 degrees of freedom, p= 0.00476

> pv <- pchisq(logrank$chisq, 1, lower.tail = F)

> {
+     plot(sf, conf.int = F, main = "MDACC to consortium, without SHP",
+         xlab = "Survival time (Month)", ylab = "Survival", cex.lab = 1.2,
+         mark = c(1, 19), cex = 1, col = 1:2, lwd = 2)
+     text(140, 0.9, pv.expr(pv), cex = 1.5)
+ }
```

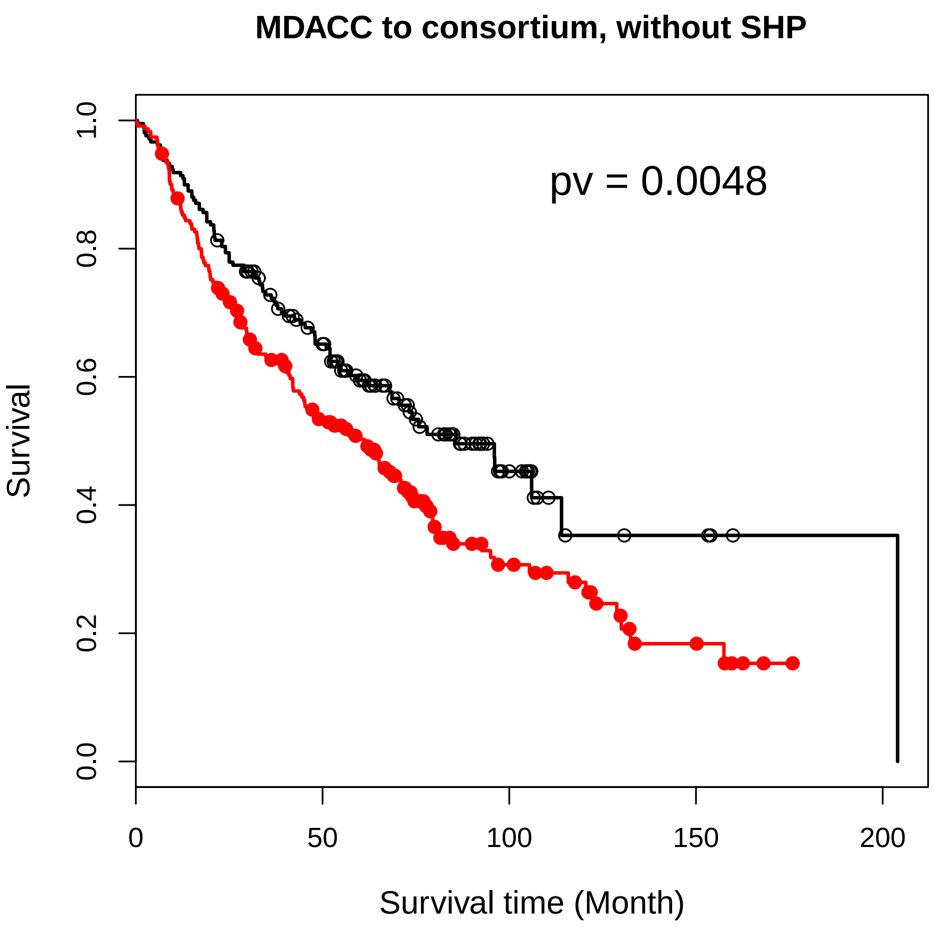

Figure 4B. Kaplan-Meier survival plot using PR in the single gene prediction model. The MDACC cohort was used as a training set and independently tested in the multi-site Consortium cohort. For this analysis mRNA expression values for SHP were removed from the dataset in order to test the effect of other NR genes as biomarkers. In this case, PR expression is the single covariable (or predictor) in the classification model that describes the survival differences, demonstrating that PR is a single-gene predictor that represents the NR gene profile when SHP expression is excluded.

Predicting survival in stage I lung cancer patients

```
> data.train <- combined[combined$type == "mda", ]
> fit <- rpart(Surv(Survival_Time, Dead) ~ ., data = data.train)
> print(fit)

n= 30

node), split, n, deviance, yval
      * denotes terminal node
```

```
1) root 30 42.274540 1.0000000
  2) SHP>=0.4814558 13  6.053077 0.1735668 *
  3) SHP< 0.4814558 17 12.545500 2.2736220 *
```

```
> data.test <- combined[combined$type == "Consortium" & combined$Stage ==
+     1, ]
> group <- ifelse(predict(fit, newdat = data.test) > 1, "High",
+     " Low")
> sf <- survfit(Surv(Survival_Time, Dead) ~ group, data = data.test)
> summary(coxph(Surv(Survival_Time, Dead) ~ group, data = data.test))
```

```
Call:
coxph(formula = Surv(Survival_Time, Dead) ~ group, data = data.test)

  n= 275

            coef exp(coef) se(coef)     z Pr(>|z|)
groupHigh 0.5566    1.7448   0.2641 2.108   0.0350 *
---
Signif. codes:  0 Ś***Š 0.001 Ś**Š 0.01 Ś*Š 0.05 Ś.Š 0.1 Ś Š 1

          exp(coef) exp(-coef) lower .95 upper .95
groupHigh     1.745     0.5731     1.040     2.928

Rsquare= 0.018   (max possible= 0.982 )
Likelihood ratio test= 5.03  on 1 df,   p=0.02493
Wald test            = 4.44  on 1 df,   p=0.03504
Score (logrank) test = 4.56  on 1 df,   p=0.03275
```

```
> logrank <- survdiff(Surv(Survival_Time, Dead) ~ group, data = data.test)
> logrank
```

```
Call:
survdiff(formula = Surv(Survival_Time, Dead) ~ group, data = data.test)

             N Observed Expected (O-E)^2/E (O-E)^2/V
group= Low  64       17     26.6      3.44      4.55
group=High 211       94     84.4      1.08      4.55

 Chisq= 4.6  on 1 degrees of freedom, p= 0.0328
```

```
> pv <- pchisq(logrank$chisq, 1, lower.tail = F)
```

```
> {
+     plot(sf, conf.int = F, main = "MDACC to consortium, SHP, stage I only",
+         xlab = "Survival time (Month)", ylab = "Survival", cex.lab = 1.2,
+         mark = c(1, 19), col = 1:2, cex = 1, lwd = 2)
+     text(140, 0.9, pv.expr(pv), cex = 1.5)
+ }
```

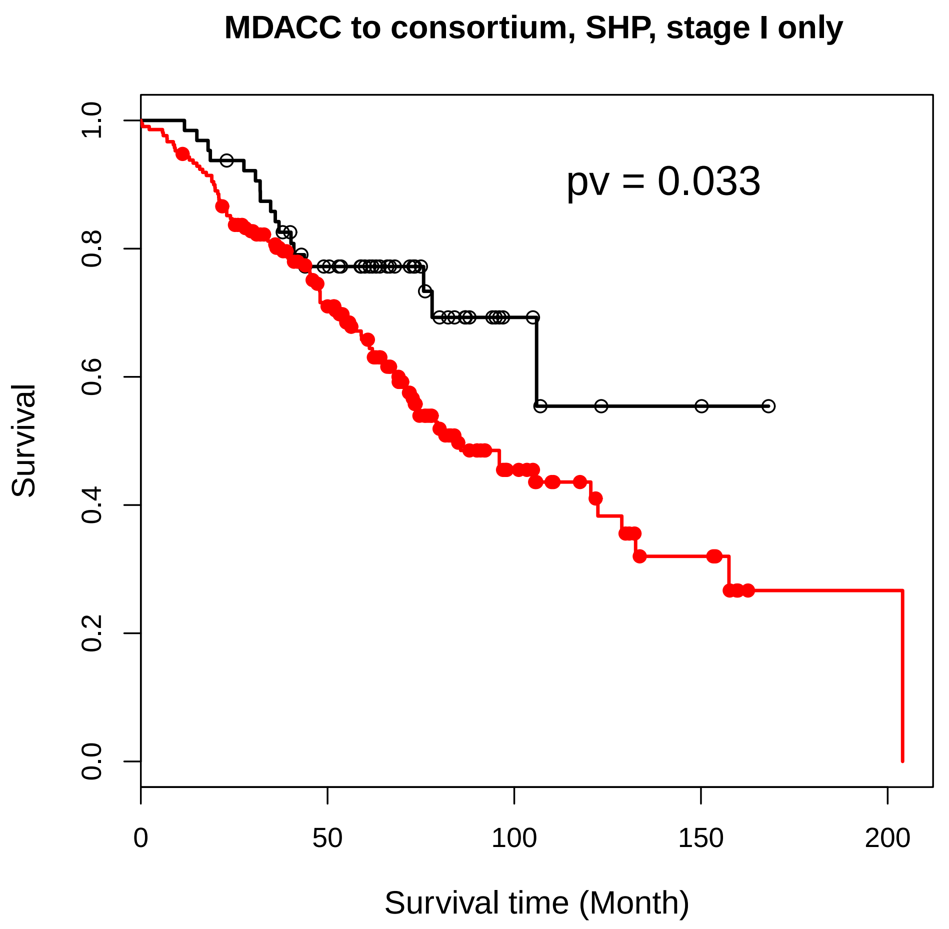

Figure 5A. Predictive model for SHP was trained in the MDACC samples and tested in the stage I lung cancer patients of the consortium cohort.

```
> data.train <- combined[combined$type == "mda", colnames(combined) !=
+     "SHP"]
> data.test <- combined[combined$type == "Consortium" & combined$Stage ==
+     1, ]
> fit <- rpart(Surv(Survival_Time, Dead) ~ ., data = data.train)
> print(fit)

n= 30

node), split, n, deviance, yval
      * denotes terminal node

1) root 30 42.274540 1.0000000
  2) PR>=0.04657526 17 12.407420 0.3528263 *
  3) PR< 0.04657526 13  8.020689 2.8993190 *
```

```
> group <- ifelse(predict(fit, newdat = data.test) > 1, "High",
+     " Low")
> sf <- survfit(Surv(Survival_Time, Dead) ~ group, data = data.test)
> summary(coxph(Surv(Survival_Time, Dead) ~ group, data = data.test))

Call:
coxph(formula = Surv(Survival_Time, Dead) ~ group, data = data.test)

  n= 275

            coef exp(coef) se(coef)     z Pr(>|z|)
groupHigh 0.3547    1.4257   0.1965 1.805   0.0711 .
---
Signif. codes:  0 Ś***Š 0.001 Ś**Š 0.01 Ś*Š 0.05 Ś.Š 0.1 Ś Š 1

          exp(coef) exp(-coef) lower .95 upper .95
groupHigh     1.426     0.7014      0.97     2.096

Rsquare= 0.012   (max possible= 0.982 )
Likelihood ratio test= 3.32  on 1 df,   p=0.0683
Wald test            = 3.26  on 1 df,   p=0.0711
Score (logrank) test = 3.29  on 1 df,   p=0.06967

> logrank <- survdiff(Surv(Survival_Time, Dead) ~ group, data = data.test)
> logrank

Call:
survdiff(formula = Surv(Survival_Time, Dead) ~ group, data = data.test)

             N Observed Expected (O-E)^2/E (O-E)^2/V
group= Low 137       44     53.5      1.67       3.3
group=High 138       67     57.5      1.55       3.3

 Chisq= 3.3  on 1 degrees of freedom, p= 0.0692

> pv <- pchisq(logrank$chisq, 1, lower.tail = F)

> {
+     plot(sf, conf.int = F, main = "MDACC to consortium, without SHP, stage I only",
+         xlab = "Survival time (Month)", ylab = "Survival", cex.lab = 1.2,
+         mark = c(1, 19), col = 1:2, cex = 1, lwd = 2)
+     text(140, 0.9, pv.expr(pv), cex = 1.5)
+ }
```

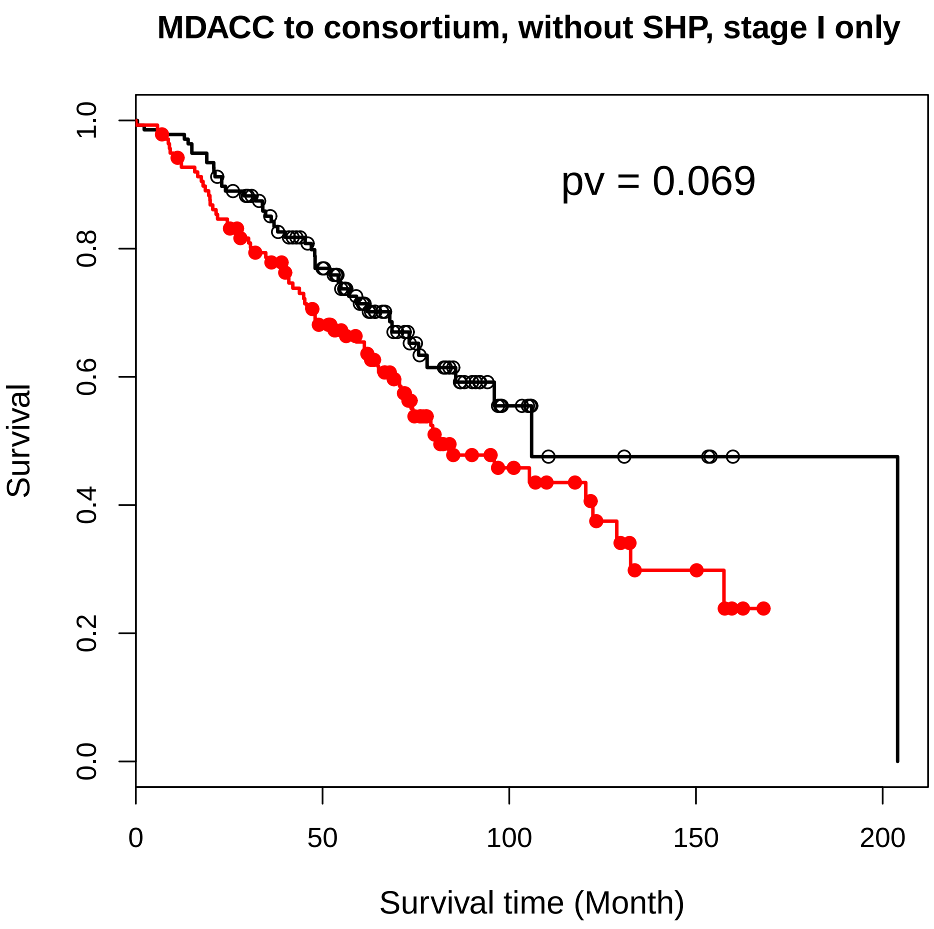

Figure 5B. Predictive model for PR was trained in the MDACC samples and tested in the stage I lung cancer patients of the consortium cohort.

Prediction with normal tissue data

```
> mda.normal <- read.csv("MDA_data_normal_Jan 24 2010.csv", row.names = 1)
> dim(mda.normal)
```

```
[1] 30 54
```

```
> mda.normal[1:4, 1:16]
```

```
    Dead Survival_Time Progression      TOE    COUP.TFb       TR4 DAX.1
737    0      84.81967           0 84.81967 0.42587400 0.4170522     0
739    1      63.90164           1 47.73770 0.71241461 0.7078501     0
749    0      30.85246           0 30.85246 0.09914638 0.5795992     0
756    0      72.95082           0 72.95082 0.49208097 0.6910128     0
```

```
        LXRb      RARa      RXRb  REV.ERBa  REV.ERBb  COUP.TFg      RORa
737 0.4245388 0.3603237 0.1484563 0.4978787 0.5986191 0.1063993 0.3778851
739 0.6956200 0.6239872 0.1837411 0.5680166 0.8705468 0.1573902 0.4897771
749 0.6110015 0.4097818 0.1818671 0.2851873 0.5119912 0.1322197 0.4820626
756 1.1302375 0.4390864 0.4070447 0.3802573 0.7447870 0.1530503 0.6154929
          GR     PPARg
737 0.4709204 0.1468401
739 0.5554739 0.6925421
749 0.4594592 0.5910218
756 0.7583135 1.1323468
```

```
> fit <- rpart(Surv(TOE, Progression) ~ ., data = mda.normal[,
+     -(1:2)])
> print(fit)
```

```
n= 30

node), split, n, deviance, yval
      * denotes terminal node

1) root 30 41.33263 1.0000000
  2) NGFIB3>=0.008717298 12 14.47527 0.3998498 *
  3) NGFIB3< 0.008717298 18 12.29514 1.9010720 *
```

The classification tree structure revealed that NGFIB3 was the single gene signature used.

```
> res <- rep(0, 30)
> for (i in 1:30) {
+     fit <- rpart(Surv(TOE, Progression) ~ ., data = mda.normal[-i,
+         -(1:2)])
+     res[i] <- (predict(fit, newdat = mda.normal[i, -(1:2)]) >
+         1)
+ }
> sf <- survfit(Surv(TOE, Progression) ~ res, data = mda.normal)
> logrank <- survdiff(Surv(TOE, Progression) ~ res, data = mda.normal)
> logrank
```

```
Call:
survdiff(formula = Surv(TOE, Progression) ~ res, data = mda.normal)

       N Observed Expected (O-E)^2/E (O-E)^2/V
res=0 12        6    13.43      4.11      10.8
res=1 18       17     9.57      5.77      10.8

 Chisq= 10.8  on 1 degrees of freedom, p= 0.000989
```

```
> pv <- pchisq(logrank$chisq, 1, lower.tail = F)
> summary(coxph(Surv(TOE, Progression) ~ res, data = mda.normal))
```

```
Call:
coxph(formula = Surv(TOE, Progression) ~ res, data = mda.normal)

  n= 30

      coef exp(coef) se(coef)     z Pr(>|z|)
res 1.5291    4.6142   0.4983 3.068  0.00215 **
---
Signif. codes:  0 Ś***Š 0.001 Ś**Š 0.01 Ś*Š 0.05 Ś.Š 0.1 Ś Š 1

    exp(coef) exp(-coef) lower .95 upper .95
res     4.614     0.2167     1.737     12.25

Rsquare= 0.306   (max possible= 0.987 )
Likelihood ratio test= 10.95  on 1 df,   p=0.000936
Wald test            = 9.42  on 1 df,   p=0.002152
Score (logrank) test = 10.87  on 1 df,   p=0.0009783

> plot(sf, main = "MDACC Normal Tissue LOOCV", xlab = "Time to recurrence (month)",
+     ylab = "Recurrence free survival", cex.lab = 1.5, mark = c(1,
+         19), cex = 1, col = 1:2, lwd = 2)
> text(60, 0.2, pv.expr(pv), cex = 1.5)
```

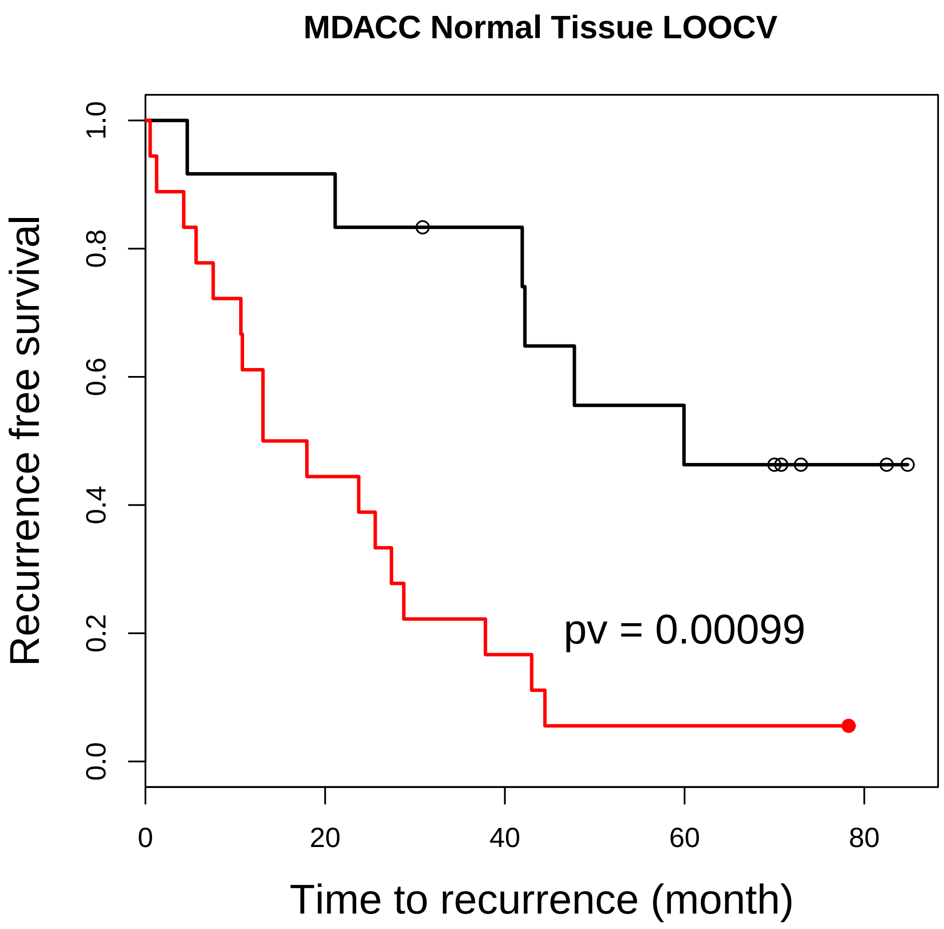

Figure S9A. Identification of NRs as prognostic biomarkers in normal lung tissue from lung cancer patients. LOOCV of recursive-partitioning tree model of the MDACC QPCR data in normal tissues shows that NGFI-B is the single gene left in the predictive model for disease progression.

```
> fit <- rpart(Surv(Survival_Time, Dead) ~ ., data = mda.normal[,
+     -(3:4)])
> print(fit)

n= 30

node), split, n, deviance, yval
      * denotes terminal node

1) root 30 42.27454 1.0000000
  2) MR>=0.04008524 18 15.84951 0.4885411 *
  3) MR< 0.04008524 12 12.15174 2.5686990 *
```

The classification tree structure revealed that MR was the single gene signature used.

```
> res <- rep(0, 30)
> for (i in 1:30) {
+     fit <- rpart(Surv(Survival_Time, Dead) ~ ., data = mda.normal[-i,
+         -(3:4)])
+     res[i] <- (predict(fit, newdat = mda.normal[i, -(3:4)]) >
+         1)
+ }
> sf <- survfit(Surv(Survival_Time, Dead) ~ res, data = mda.normal)
> logrank <- survdiff(Surv(Survival_Time, Dead) ~ res, data = mda.normal)
> logrank

Call:
survdiff(formula = Surv(Survival_Time, Dead) ~ res, data = mda.normal)

       N Observed Expected (O-E)^2/E (O-E)^2/V
res=0 20        9     12.1     0.794      2.80
res=1 10        8      4.9     1.960      2.80

 Chisq= 2.8  on 1 degrees of freedom, p= 0.0944

> pv <- pchisq(logrank$chisq, 1, lower.tail = F)
> summary(coxph(Surv(Survival_Time, Dead) ~ res, data = mda.normal))

Call:
coxph(formula = Surv(Survival_Time, Dead) ~ res, data = mda.normal)

  n= 30

      coef exp(coef) se(coef)     z Pr(>|z|)
res 0.7994    2.2243   0.4902 1.631    0.103

    exp(coef) exp(-coef) lower .95 upper .95
res     2.224     0.4496     0.851     5.814

Rsquare= 0.082   (max possible= 0.963 )
Likelihood ratio test= 2.56  on 1 df,   p=0.1098
Wald test            = 2.66  on 1 df,   p=0.1029
Score (logrank) test = 2.8  on 1 df,   p=0.09444

> plot(sf, main = "MDACC Normal Tissue LOOCV", xlab = "Survival time (month)",
+     ylab = "Survival", cex.lab = 1.5, mark = c(1, 19), cex = 1,
+     lwd = 2, col = 1:2)
> text(20, 0.2, pv.expr(pv), cex = 1.5)
```

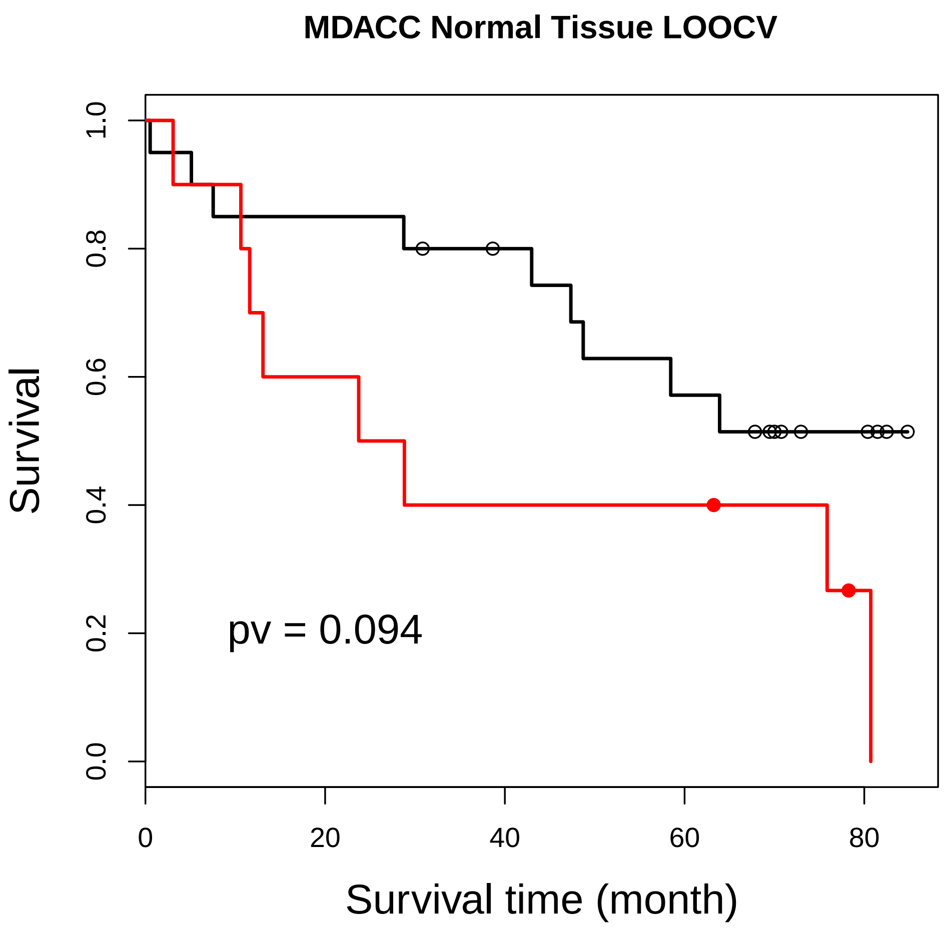

Figure S9B. Identification of NRs as prognostic biomarkers in normal lung tissue from lung cancer patients. LOOCV of recursive-partitioning tree model of the MDACC QPCR data in normal tissues shows that MR is the single gene left in the predictive model for overall survival.

Unsupervised clustering analysis for consortium data

```
> Consortium <- read.csv("Consortium_data.csv", row.names = 1)
> hc <- hclust(dist(Consortium[, 10:57]))
> plot(hc)
> cluster <- cutree(hc, k = 2)
> sf <- survfit(Surv(month, death) ~ cluster, data = Consortium)
> summary(coxph(Surv(month, death) ~ cluster, data = Consortium))

Call:
coxph(formula = Surv(month, death) ~ cluster, data = Consortium)
```

```
  n=440 (2 observations deleted due to missingness)

          coef exp(coef) se(coef)     z Pr(>|z|)
cluster 0.2662    1.3050   0.1440 1.849   0.0645 .
---
Signif. codes:  0 Ś***Š 0.001 Ś**Š 0.01 Ś*Š 0.05 Ś.Š 0.1 Ś Š 1

        exp(coef) exp(-coef) lower .95 upper .95
cluster     1.305     0.7663     0.984     1.730

Rsquare= 0.007   (max possible= 0.997 )
Likelihood ratio test= 3.29  on 1 df,   p=0.06987
Wald test            = 3.42  on 1 df,   p=0.06451
Score (logrank) test = 3.44  on 1 df,   p=0.06373

> logrank <- survdiff(Surv(month, death) ~ cluster, data = Consortium)
> logrank

Call:
survdiff(formula = Surv(month, death) ~ cluster, data = Consortium)

n=440, 2 observations deleted due to missingness.

            N Observed Expected (O-E)^2/E (O-E)^2/V
cluster=1 333      168    180.1      0.81      3.43
cluster=2 107       68     55.9      2.61      3.43

 Chisq= 3.4  on 1 degrees of freedom, p= 0.0638

> pv <- pchisq(logrank$chisq, 1, lower.tail = F)

> plot(sf, conf.int = F, main = "Consortium unsupervised clustering",
+     xlab = "Survival time (Month)", ylab = "Survival", cex.lab = 1.2,
+     mark = c(1, 19), col = 1:2, cex = 1.5, lty = 1, lwd = 2)
> text(100, 0.9, pv.expr(pv), cex = 1.5)
```

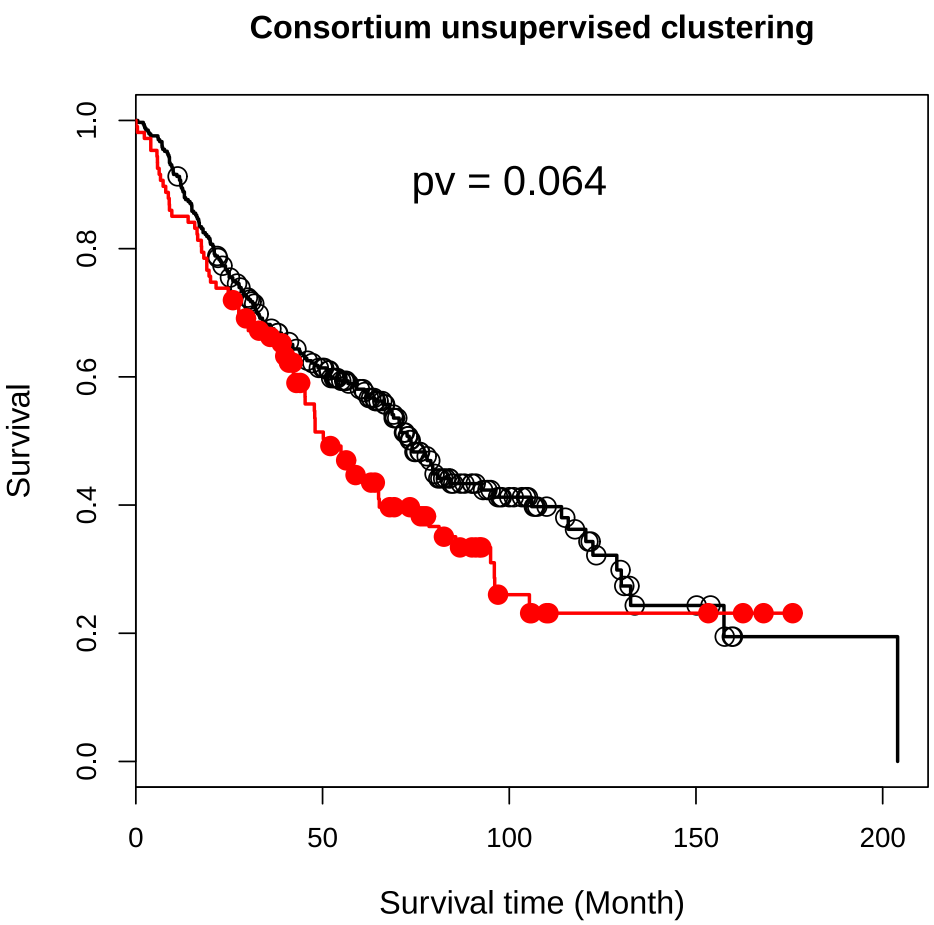

Figure S11. Unsupervised hierarchical cluster analysis of the microarray signature of the 48 NRs divides the consortium samples into two clusters.

Appendix

```
> sessionInfo()

R version 2.10.0 (2009-10-26)
i386-pc-mingw32

locale:
[1] LC_COLLATE=English_United States.1252
[2] LC_CTYPE=English_United States.1252
[3] LC_MONETARY=English_United States.1252
[4] LC_NUMERIC=C
[5] LC_TIME=English_United States.1252
```

```
attached base packages:
[1] splines   stats     graphics  grDevices utils     datasets  methods
[8] base

other attached packages:
[1] survivalROC_1.0.0 rpart_3.1-45      survival_2.35-7
```

Text S2: Sweave Document Part 2

Nuclear Receptor Expression Profiling Defines a Set of Prognostic Biomarkers for Lung Cancer

Yangsik Jeong, Yang Xie, Guanghua Xiao, Carmen Behrens, Luc Girard,
Ignacio I Wistuba, John D Minna & David J Mangelsdorf

```
> library(survival)
> library(rpart)
> library(survivalROC)
```

```
> pv.expr <- function(x, digits = 1) {
+     if (!x)
+         return(0)
+     exponent <- floor(log10(x))
+     base <- round(x/10^exponent, digits)
+     ifelse(x > 1e-04, paste("pv = ", base * (10^exponent), sep = ""),
+         paste("pv = ", base, "E", exponent, sep = ""))
+ }
```

Tomida et al dataset

Preprocess of Tomida et al dataset

The GSE3141 (Series Matrix File), platform annotation file and patient clinical information file were downloaded from GEO website(http://www.ncbi.nlm.nih.gov/geo/) and saved as csv files

```
> expr <- read.csv("GSE13213_series_matrix.csv", row.names = 1,
+     na.strings = "null")
> dim(expr)
```

```
[1] 41000   117
```

```
> expr[1:4, 1:6]
```

```
             GSM333673 GSM333674 GSM333675 GSM333676 GSM333677 GSM333678
A_23_P100001    0.4340    1.3516   -1.3959   -0.4620   -0.4403   -0.7784
A_23_P100011   -1.1297   -1.3921   -2.9324   -1.8783   -2.4189   -2.4422
A_23_P100022   -4.0023   -4.5064   -4.5907   -4.0565   -4.4643   -4.2189
A_23_P100056   -0.6304   -2.7661   -1.5951   -0.6439   -0.4639   -0.2863
```

```
> range(expr, na.rm = T)

[1] -6.6439 12.4653

> id <- read.csv("NR probe ID New.csv")
> head(id)

  Probe.Set.ID mRNA.Accession Formal.Name Receptor
1  211110_s_at      NM_000044       NR3C4       AR
2    211621_at      NM_000044       NR3C4       AR
3    207007_at      NM_005122       NR1I3      CAR
4    209505_at      NM_005654       NR2F1 COUP.TFa
5  209506_s_at      NM_005654       NR2F1 COUP.TFa
6  209119_x_at      NM_021005       NR2F2 COUP.TFb

> dim(id)

[1] 110   4

> length(unique(id$Receptor))

[1] 48

> first <- function(x) {
+     x[1]
+ }
> uid <- aggregate(id[, -1], by = list(acc = id$mRNA.Accession),
+     first)
```

Extract NR expression from Tomida data. If there are multiple probes corresponding to a single NR, then we take the average expression of those probes.

```
> acc <- read.csv("Tomida array annotation.csv")
> head(acc)

           ID    GB_ACC GENE_SYMBOL
1 A_23_P100001 NM_207446     FAM174B
2 A_23_P100011 NM_005829       AP3S2
3 A_23_P100022 NM_014848        SV2B
4 A_23_P100056 NM_194272      RBPMS2
5 A_23_P100074 NM_020371        AVEN
6 A_23_P100092 NM_152455     ZSCAN29

> length(intersect(uid$acc, acc$GB_ACC))

[1] 35

> nr.id <- merge(uid, acc, by.x = "acc", by.y = "GB_ACC", all = F)
> length(unique(nr.id$Receptor))

[1] 35
```

```
> expr1 <- merge(nr.id, expr, by.x = "ID", by.y = "row.names",
+     all = F)
> expr1[1:3, 1:8]

           ID       acc mRNA.Accession Formal.Name Receptor GENE_SYMBOL
1 A_23_P108326 NM_005234      NM_005234       NR2F6 COUP.TFg       NR2F6
2 A_23_P109785 NM_003889      NM_003889       NR1I2      PXR       NR1I2
3 A_23_P113111 NM_000044      NM_000044       NR3C4       AR          AR
  GSM333673 GSM333674
1   -0.4131   -0.3978
2    0.5656        NA
3    0.0621    2.5155

> nr.expr <- aggregate(expr1[, -(1:6)], by = list(gene = expr1$Receptor),
+     mean, na.rm = T)
> dim(nr.expr)

[1]  35 118

> expr2 <- t(nr.expr[, -1])
> colnames(expr2) <- nr.expr[, 1]
> expr2[is.na(expr2)] <- 0
> head(expr2)

               AR COUP.TFa COUP.TFb COUP.TFg   DAX.1     ERa    ERRa    ERRb
GSM333673  0.0621   0.3707 -1.16340  -0.4131 -6.6439 1.57545 -0.4759 -0.3129
GSM333674  2.5155   2.3491 -0.76735  -0.3978 -6.6439 1.66615  0.1230 -0.3548
GSM333675  0.3311   0.8098 -1.11700  -0.6827 -6.6439 1.59650  0.0426 -0.3129
GSM333676 -0.6943   1.9084 -0.55090  -0.6897 -6.6439 1.82285 -0.4620 -0.5821
GSM333677  1.1622   1.9873 -0.87300  -1.3076 -6.6439 0.63430 -0.7155 -0.5270
GSM333678  2.3802   0.6489 -1.11425  -0.1633 -6.6439 0.71520 -0.3129 -0.0544
              FXR  HNF4a   HNF4g   LXRa     LXRb     MR  NGFIB3  NURR1
GSM333673  0.0000 0.9568  0.0000 1.5859  0.00515 1.9657  1.8856 1.6794
GSM333674  0.0000 0.1124  0.0000 1.1177 -0.13730 2.2126  3.0533 3.2096
GSM333675  0.0000 0.0000 -0.6439 1.1757 -0.48500 1.3482  2.3716 1.6443
GSM333676  0.0909 0.0468  0.0000 1.9358 -0.62265 2.0963  1.9366 1.4356
GSM333677  0.0000 0.0000  0.5566 1.7732 -0.93290 2.6645  2.3843 1.1667
GSM333678 -1.4501 0.5945  0.0000 0.5636 -0.46235 1.6767 -0.3273 0.7364
                PPARa   PPARd      PR    PXR    RARb     RARg REV.ERBb   RORb
GSM333673  0.37810000 1.18010 1.13605 0.5656  0.8976 -0.39870   0.5099 1.0065
GSM333674 -0.35563333 0.50690 1.31845 0.0000 -0.5821 -0.13160  -0.1250 1.6677
GSM333675 -0.18176667 1.27530 1.78100 0.0000 -1.2481 -0.56450  -0.1203 0.0000
GSM333676 -0.34030000 1.35470 0.83125 0.0000 -0.1714 -0.56460  -0.4860 0.1401
GSM333677  0.13425000 0.88390 3.31495 0.0000 -0.5522 -0.63655   0.8229 2.7119
GSM333678 -0.02793333 2.06465 0.33400 0.0000 -0.9296  0.12905  -0.4325 0.0000
              RORg     RXRa    RXRb RXRg   SF-1     SHP     TLX     TR2     TR4
GSM333673  0.43705  0.81815 -1.4383    0 0.5351 -3.8262 -0.0710 -0.5208 -0.1219
GSM333674  1.03510  0.16365 -1.0801    0 0.3896 -4.0856 -1.3846 -0.3622 -0.6104
GSM333675  0.92105  0.19785 -0.6712    0 0.0000 -4.2905 -1.4941  0.0881 -0.4719
```

```
GSM333676  0.34520 -0.01020 -1.5735    0 0.7407 -3.0710 -1.3511 -0.3040 -0.6574
GSM333677 -0.46575  0.43630 -1.2481    0 0.8718 -1.4422 -2.6804 -0.1376 -0.6712
GSM333678  1.64575  0.77720 -1.5778    0 0.7390 -4.2962  0.5811 -0.1763  0.2216
              TRa     TRb
GSM333673 -0.6104 -1.1329
GSM333674  0.5200 -0.9078
GSM333675  0.5821 -3.1329
GSM333676  0.0101  0.1622
GSM333677  0.0342 -1.1203
GSM333678 -1.7322 -0.7394
```

Read clinical information, merge the clinical information and NR expression, and output the results as csv file.

```
> clin <- read.csv("AD117_patient_info.csv", row.names = 1)
> head(clin)
```

```
         Cohort Age Sex Histology Smoking..BI. TNM..Pathological.
AD001 dataset I  71   M        AD         1020             T2N0M0
AD002 dataset I  49   F        AD            0             T1N0M0
AD003 dataset I  51   F        AD            0             T2N2M0
AD004 dataset I  51   F        AD            0             T1N1M0
AD005 dataset I  67   F        AD            0             T1N2M0
AD006 dataset I  66   M        AD          100             T3N1M0
      Stage..Pathological.. Status Survival..days. Evidence.of.relapse
AD001                    IB   Dead            1326                   Y
AD002                    IA  Alive            3275                   N
AD003                  IIIA   Dead            1687                   Y
AD004                   IIA  Alive            3214                   N
AD005                  IIIA   Dead            1200                   Y
AD006                  IIIA   Dead             223                   Y
      Site.of.relapse EGFR.status K.ras.Status p53.Status
AD001              PM         Mut           Wt         Wt
AD002                          Wt           Wt         Wt
AD003              PM          Wt           Wt        Mut
AD004                          Wt           Wt         Wt
AD005              PM         Mut           Wt        Mut
AD006           Brain         Mut           Wt         Wt
```

```
> death <- as.numeric(clin$Status) - 1
> month <- clin$Survival..days./30.5
> stage <- rep(0, dim(clin)[1])
> stage[clin$Stage..Pathological.. %in% c("IA", "IB")] <- 1
> stage[clin$Stage..Pathological.. %in% c("IIA", "IIB")] <- 2
> stage[clin$Stage..Pathological.. %in% c("IIIA", "IIIB")] <- 3
> out <- data.frame(month, death, stage, expr2)
> write.csv(out, "Tomida data.csv", row.names = T)
```

Use MDACC data to predict the survival in Tomida et al dataset

Read MDACC and Tomida datasets.

```
> mda <- read.csv("MDA_data_Jan 24 2010.csv", row.names = 1)
> mda.pcr <- mda[, -(1:4)]
> mda.pcr[mda.pcr == 0] <- min(mda.pcr[mda.pcr != 0])
> mda[, -(1:4)] <- mda.pcr <- log2(mda.pcr)
```

```
> Tomida <- read.csv("Tomida data.csv", row.names = 1)
> head(Tomida)
```

```
               month death stage      AR COUP.TFa COUP.TFb COUP.TFg   DAX.1
GSM333673  43.475410     1     1  0.0621   0.3707 -1.16340  -0.4131 -6.6439
GSM333674 107.377049     0     1  2.5155   2.3491 -0.76735  -0.3978 -6.6439
GSM333675  55.311475     1     3  0.3311   0.8098 -1.11700  -0.6827 -6.6439
GSM333676 105.377049     0     2 -0.6943   1.9084 -0.55090  -0.6897 -6.6439
GSM333677  39.344262     1     3  1.1622   1.9873 -0.87300  -1.3076 -6.6439
GSM333678   7.311475     1     3  2.3802   0.6489 -1.11425  -0.1633 -6.6439
              ERa    ERRa    ERRb     FXR  HNF4a   HNF4g   LXRa     LXRb     MR
GSM333673 1.57545 -0.4759 -0.3129  0.0000 0.9568  0.0000 1.5859  0.00515 1.9657
GSM333674 1.66615  0.1230 -0.3548  0.0000 0.1124  0.0000 1.1177 -0.13730 2.2126
GSM333675 1.59650  0.0426 -0.3129  0.0000 0.0000 -0.6439 1.1757 -0.48500 1.3482
GSM333676 1.82285 -0.4620 -0.5821  0.0909 0.0468  0.0000 1.9358 -0.62265 2.0963
GSM333677 0.63430 -0.7155 -0.5270  0.0000 0.0000  0.5566 1.7732 -0.93290 2.6645
GSM333678 0.71520 -0.3129 -0.0544 -1.4501 0.5945  0.0000 0.5636 -0.46235 1.6767
           NGFIB3  NURR1       PPARa   PPARd      PR    PXR    RARb     RARg
GSM333673  1.8856 1.6794  0.37810000 1.18010 1.13605 0.5656  0.8976 -0.39870
GSM333674  3.0533 3.2096 -0.35563333 0.50690 1.31845 0.0000 -0.5821 -0.13160
GSM333675  2.3716 1.6443 -0.18176667 1.27530 1.78100 0.0000 -1.2481 -0.56450
GSM333676  1.9366 1.4356 -0.34030000 1.35470 0.83125 0.0000 -0.1714 -0.56460
GSM333677  2.3843 1.1667  0.13425000 0.88390 3.31495 0.0000 -0.5522 -0.63655
GSM333678 -0.3273 0.7364 -0.02793333 2.06465 0.33400 0.0000 -0.9296  0.12905
          REV.ERBb   RORb     RORg     RXRa    RXRb RXRg   SF.1     SHP     TLX
GSM333673   0.5099 1.0065  0.43705  0.81815 -1.4383    0 0.5351 -3.8262 -0.0710
GSM333674  -0.1250 1.6677  1.03510  0.16365 -1.0801    0 0.3896 -4.0856 -1.3846
GSM333675  -0.1203 0.0000  0.92105  0.19785 -0.6712    0 0.0000 -4.2905 -1.4941
GSM333676  -0.4860 0.1401  0.34520 -0.01020 -1.5735    0 0.7407 -3.0710 -1.3511
GSM333677   0.8229 2.7119 -0.46575  0.43630 -1.2481    0 0.8718 -1.4422 -2.6804
GSM333678  -0.4325 0.0000  1.64575  0.77720 -1.5778    0 0.7390 -4.2962  0.5811
              TR2     TR4     TRa     TRb
GSM333673 -0.5208 -0.1219 -0.6104 -1.1329
GSM333674 -0.3622 -0.6104  0.5200 -0.9078
GSM333675  0.0881 -0.4719  0.5821 -3.1329
GSM333676 -0.3040 -0.6574  0.0101  0.1622
GSM333677 -0.1376 -0.6712  0.0342 -1.1203
GSM333678 -0.1763  0.2216 -1.7322 -0.7394
```

Merge the MDACC and Tomida datasets.

```
> Tomida.expr <- Tomida[, -(1:3)]
> Tomida.expr <- scale(Tomida.expr)
> mda.surv <- mda[, -(3:4)]
> mda.surv[, -(1:2)] <- scale(mda.surv[, -(1:2)])
> common.gene <- intersect(colnames(mda.surv)[-(1:2)], colnames(Tomida.expr))
> setdiff(colnames(mda.surv)[-(1:2)], colnames(Tomida.expr))

 [1] "RARa"     "REV.ERBa" "RORa"     "GR"       "PPARg"    "PPARd2"
 [7] "NOR1"     "VDR"      "GCNF"     "ERb"      "PNR"      "LRH.1"
[13] "ERRg"     "PPARg2"   "CAR"

> setdiff(colnames(Tomida.expr), colnames(mda.surv)[-(1:2)])

character(0)

> length(common.gene)

[1] 35

> mda.data <- data.frame(type = "mda", Stage = NA, mda.surv[, 1:2],
+     mda.surv[, common.gene])
> Tomida.data <- data.frame(type = "Tomida", Stage = Tomida$stage,
+     Dead = Tomida$death, Survival_Time = Tomida$month, Tomida.expr[,
+         common.gene])
> combined <- data.frame(rbind(mda.data, Tomida.data))

> data.train <- combined[combined$type == "mda", ]
> data.test <- combined[combined$type == "Tomida", ]
> fit <- rpart(Surv(Survival_Time, Dead) ~ ., data = data.train)
> print(fit)

n= 30

node), split, n, deviance, yval
      * denotes terminal node

1) root 30 42.274540 1.0000000
  2) SHP>=0.4814558 13  6.053077 0.1735668 *
  3) SHP< 0.4814558 17 12.545500 2.2736220 *

> group <- ifelse(predict(fit, newdat = data.test) > 1, "High",
+     " Low")
> sf <- survfit(Surv(Survival_Time, Dead) ~ group, data = data.test)
> summary(coxph(Surv(Survival_Time, Dead) ~ group, data = data.test))

Call:
coxph(formula = Surv(Survival_Time, Dead) ~ group, data = data.test)

  n= 117
```

```
            coef exp(coef) se(coef)     z Pr(>|z|)
groupHigh 1.0869    2.9651   0.4088 2.659  0.00784 **
---
Signif. codes:  0 Ś***Š 0.001 Ś**Š 0.01 Ś*Š 0.05 Ś.Š 0.1 Ś Š 1

          exp(coef) exp(-coef) lower .95 upper .95
groupHigh     2.965     0.3373     1.331     6.607

Rsquare= 0.074   (max possible= 0.976 )
Likelihood ratio test= 8.99  on 1 df,   p=0.002712
Wald test            = 7.07  on 1 df,   p=0.007842
Score (logrank) test = 7.79  on 1 df,   p=0.00526
```

```
> logrank <- survdiff(Surv(Survival_Time, Dead) ~ group, data = data.test)
> logrank
```

```
Call:
survdiff(formula = Surv(Survival_Time, Dead) ~ group, data = data.test)

            N Observed Expected (O-E)^2/E (O-E)^2/V
group= Low 34        7     16.2      5.19      7.79
group=High 83       42     32.8      2.55      7.79

 Chisq= 7.8  on 1 degrees of freedom, p= 0.00526
```

```
> pv <- pchisq(logrank$chisq, 1, lower.tail = F)
```

```
> plot(sf, conf.int = F, main = "MDACC to Tomida dataset", xlab = "Survival time (Month)",
+     ylab = "Survival", cex.lab = 1.2, mark = c(1, 19), cex = 1,
+     col = 1:2, , lwd = 2)
> text(60, 0.2, pv.expr(pv), cex = 1.5)
```

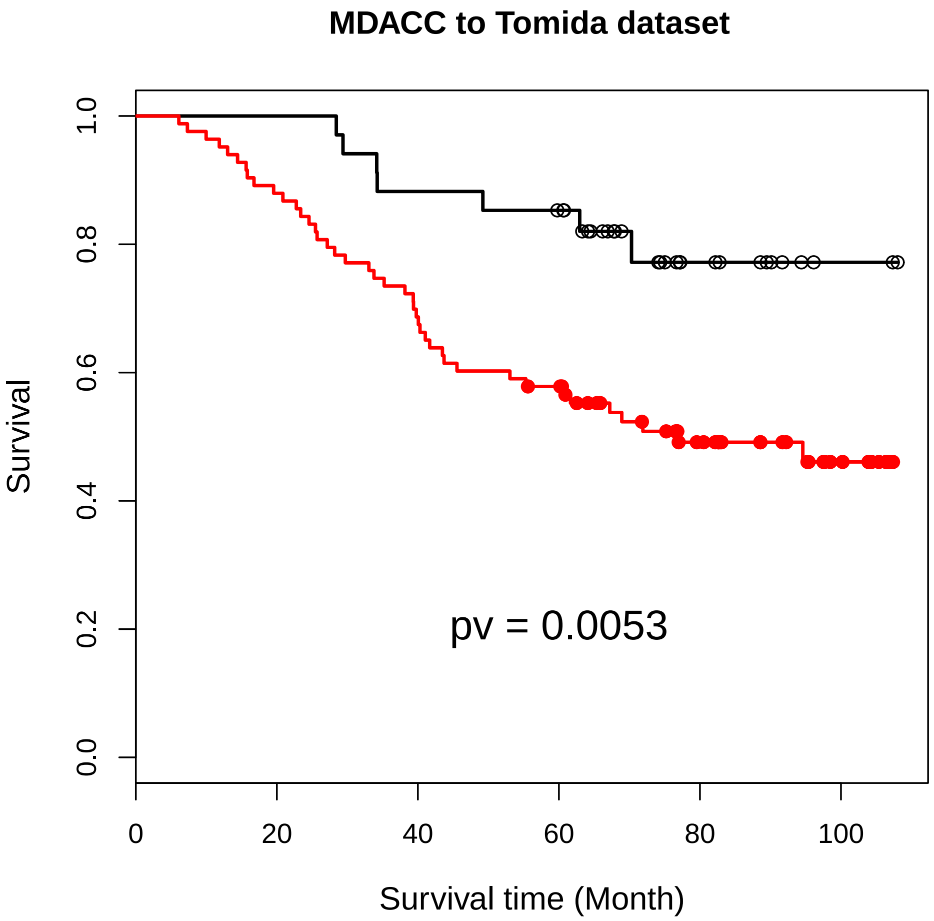

Figure S4. Use NR gene signature developed from MDACC QPCR dataset and validated on Tomida et al microarray dataset.

Raponi et al dataset

Preprocess of Raponi et al dataset

The GDS2373 (SOFT file)and patient clinical information file were downloaded from GEO and saved as csv files

```
> dat <- read.csv("GDS2373.csv", row.names = 1)
> range(dat[, -1])

[1]      0.2 92227.6

> dat[, -1] <- log2(dat[, -1])
```

```
> id <- read.csv("NR probe ID New.csv")
> head(id)

  Probe.Set.ID mRNA.Accession Formal.Name Receptor
1  211110_s_at      NM_000044       NR3C4       AR
2    211621_at      NM_000044       NR3C4       AR
3    207007_at      NM_005122       NR1I3      CAR
4    209505_at      NM_005654       NR2F1 COUP.TFa
5  209506_s_at      NM_005654       NR2F1 COUP.TFa
6  209119_x_at      NM_021005       NR2F2 COUP.TFb

> dim(id)

[1] 110   4

> length(unique(id$Receptor))

[1] 48
```

Extract NR expression from Raponi data. If there are multiple probes corresponding to a single NR, then we take the average expression of those probes.

```
> nr <- merge(id, dat, by.x = "Probe.Set.ID", by.y = "row.names")
> dim(nr)

[1] 110 135

> nr[1:4, 1:6]

  Probe.Set.ID mRNA.Accession Formal.Name Receptor IDENTIFIER GSM102191
1      1316_at         X55005        THRA      TRa       THRA  7.768846
2      1487_at      NM_004451       NR3B1     ERRa      ESRRA  9.134170
3  201865_x_at      NM_000176       NR3C1       GR   AI432196 10.066224
4  201866_s_at      NM_000176       NR3C1       GR      NR3C1  8.622418

> nr.expr <- aggregate(nr[, -(1:5)], by = list(gene = nr$Receptor),
+     mean)
> dim(nr.expr)

[1]  48 131

> nrs <- data.frame(t(nr.expr[, -1]))
> colnames(nrs) <- nr.expr[, 1]
```

Read clinical information, merge the clinical information and NR expression, and output the results as csv file.

```
> ann <- read.csv("Raponi clinical.csv", row.names = 1)
> head(ann)
```

```
          RNA.array.SCC.ID Histology Operation.date Last.visit.time
GSM102114             LS-1       SCC      12/2/1991                
GSM102182            LS-10       SCC      9/29/1992                
GSM102225           LS-100       SCC      6/11/2001       2/10/2003
GSM102160           LS-101       SCC      4/30/2001       3/26/2004
GSM102226           LS-102       SCC      9/12/2001       2/17/2004
GSM102161           LS-103       SCC      7/27/2001                
          Date.of.death Survival.time..mo.
GSM102114     2/25/1993               15.0
GSM102182     6/20/1993                9.7
GSM102225                             20.3
GSM102160                             35.4
GSM102226                             29.6
GSM102161    11/27/2003               28.4
                                Other.disease STAGE T N M differentiation AGE
GSM102114                                 unknown   IIb 3 0 0        mod-poor  75
GSM102182                         hyperthyroidism    Ib 2 0 0            poor  61
GSM102225                                diabetes    Ib 2 0 0             mod  72
GSM102160                                 unknown   IIb 2 1 0             mod  75
GSM102226 Chronic obstructive pulmonary disease    Ib 2 0 0             mod  76
GSM102161     diabetes, coronary artery disease   IIb 2 1 0        well-mod  58
          SEX RACE        SMOKING.HX
GSM102114   M    w           40 pk/yr
GSM102182   F    w        Non-smoker
GSM102225   M    w  2pk/day - 25 yrs
GSM102160   M    w           unknown
GSM102226   F    w   1pk/day - 40yrs
GSM102161   M    w 1.5pk/day - 40yrs
```

```
> ann$death <- rep(NA, dim(ann)[1])
> ann$death[ann$Last.visit.time != ""] <- 0
> ann$death[ann$Date.of.death != ""] <- 1
> ann$stage <- rep(NA, dim(ann)[1])
> ann$stage[as.character(ann$STAGE) %in% c("Ia", "Ib")] <- 1
> ann$stage[as.character(ann$STAGE) %in% c("IIa", "IIb")] <- 2
> ann$stage[as.character(ann$STAGE) %in% c("IIIa", "IIIb")] <- 3
> out <- merge(ann[, c(6, 17, 18)], nrs, by = "row.names")
> dim(out)
```

```
[1] 130  52
```

```
> colnames(out)[1:2] <- c("NR", "month")
> write.csv(out, "Raponi data.csv", row.names = F)
```

Use Consortium dataset to predict the survival in Raponi et al dataset

Read Consortium and Raponi datasets.

```
> Consortium <- read.csv("Consortium_data.csv", row.names = 1)
> Consortium.expr <- Consortium[, 10:57]
```

```
> Consortium.expr <- scale(Consortium.expr)
> Raponi <- read.csv("Raponi data.csv", row.names = 1)
```

Merge the Consortium and Raponi datasets.

```
> Raponi.expr <- Raponi[, -(1:3)]
> Raponi.expr <- scale(Raponi.expr)
> common.gene <- intersect(colnames(Raponi)[-(1:3)], colnames(Consortium.expr))
> length(common.gene)

[1] 48

> Raponi.data <- data.frame(type = "Raponi", Stage = Raponi$stage,
+     Dead = Raponi$death, Survival_Time = Raponi$month, Raponi.expr[,
+         common.gene])
> Consortium.data <- data.frame(type = "Consortium", Stage = Consortium$stage,
+     Dead = Consortium$death, Survival_Time = Consortium$month,
+     Consortium.expr[, common.gene])
> combined <- data.frame(rbind(Raponi.data, Consortium.data))

> data.train <- combined[combined$type == "Consortium", -2]
> data.test <- combined[combined$type == "Raponi", -2]
> fit <- rpart(Surv(Survival_Time, Dead) ~ ., data = data.train)
> print(fit)

n=440 (2 observations deleted due to missingness)

node), split, n, deviance, yval
      * denotes terminal node

   1) root 440 650.960700 1.0000000
     2) SF.1>=-1.600797 422 612.349500 0.9473605
       4) PPARd< 1.546829 393 557.598200 0.8889341
         8) RORa>=-1.110901 353 488.590600 0.8127724
          16) RARa>=-0.8263496 282 357.962100 0.7005085
            32) RARg< 0.7062089 206 236.112100 0.5957128
              64) NURR1< -1.126548 22   7.912262 0.1177802 *
              65) NURR1>=-1.126548 184 215.220500 0.6709268
               130) PXR>=1.269161 9   1.729126 0.1354368 *
               131) PXR< 1.269161 175 206.439800 0.7109939
                 262) TR2>=1.257944 11   4.844043 0.1918291 *
                 263) TR2< 1.257944 164 194.013000 0.7694938
                   526) LXRa>=-0.8594324 134 144.483400 0.6588147 *
                   527) LXRa< -0.8594324 30  42.461040 1.3332290
                    1054) ERa>=0.1125855 13  13.110410 0.6988573 *
                    1055) ERa< 0.1125855 17  21.932320 2.1376250 *
            33) RARg>=0.7062089 76 113.805200 1.0449810
              66) GR>=0.1164013 21  28.988230 0.4769354
               132) NGFIB3>=0.2269965 9   1.775888 0.1120559 *
```

```
              133) NGFIB3< 0.2269965 12  18.553220 1.0365880 *
             67) GR< 0.1164013 55  76.323680 1.3577180
              134) ERa>=1.44486 7   3.714183 0.2946938 *
              135) ERa< 1.44486 48  61.587110 1.6885060
                270) FXR>=1.1885 7   7.610207 0.3961379 *
                271) FXR< 1.1885 41  44.954860 2.0384140 *
         17) RARa< -0.8263496 71 118.121600 1.2993340
           34) SHP>=0.9231351 12  15.193210 0.4609037 *
           35) SHP< 0.9231351 59  95.018530 1.5203210
             70) DAX.1< -0.4961372 17  22.775660 0.6645850 *
             71) DAX.1>=-0.4961372 42  59.723230 2.1019450
              142) LXRb>=-0.3823468 15  17.205720 1.1617770 *
              143) LXRb< -0.3823468 27  32.151590 3.1673350 *
        9) RORa< -1.110901 40  54.464440 1.8193850
         18) AR>=-0.4973712 24  24.203220 1.1693540 *
         19) AR< -0.4973712 16  18.400040 3.6731330 *
      5) PPARd>=1.546829 29  40.620470 2.1935050
       10) PPARg< 0.3242693 19  22.356910 1.4967360 *
       11) PPARg>=0.3242693 10  11.316950 3.9286630 *
    3) SF.1< -1.600797 18  19.521260 3.1164310 *

> group <- ifelse(predict(fit, newdat = data.test) > 1, "High",
+     " Low")
> table(group)

group
 Low High
  70   60

> sf <- survfit(Surv(Survival_Time, Dead) ~ group, data = data.test)
> summary(coxph(Surv(Survival_Time, Dead) ~ group, data = data.test))

Call:
coxph(formula = Surv(Survival_Time, Dead) ~ group, data = data.test)

  n= 130

            coef exp(coef) se(coef)     z Pr(>|z|)
groupHigh 0.5759    1.7787   0.2460 2.342   0.0192 *
---
Signif. codes:  0 Ś***Š 0.001 Ś**Š 0.01 Ś*Š 0.05 Ś.Š 0.1 Ś Š 1

          exp(coef) exp(-coef) lower .95 upper .95
groupHigh     1.779     0.5622     1.098     2.881

Rsquare= 0.041   (max possible= 0.988 )
Likelihood ratio test= 5.51  on 1 df,   p=0.01895
Wald test            = 5.48  on 1 df,   p=0.01920
Score (logrank) test = 5.63  on 1 df,   p=0.01767
```

```
> logrank <- survdiff(Surv(Survival_Time, Dead) ~ group, data = data.test)
> logrank

Call:
survdiff(formula = Surv(Survival_Time, Dead) ~ group, data = data.test)

            N Observed Expected (O-E)^2/E (O-E)^2/V
group= Low 70       31     40.5      2.23      5.62
group=High 60       37     27.5      3.28      5.62

 Chisq= 5.6  on 1 degrees of freedom, p= 0.0177

> pv <- pchisq(logrank$chisq, 1, lower.tail = F)
```

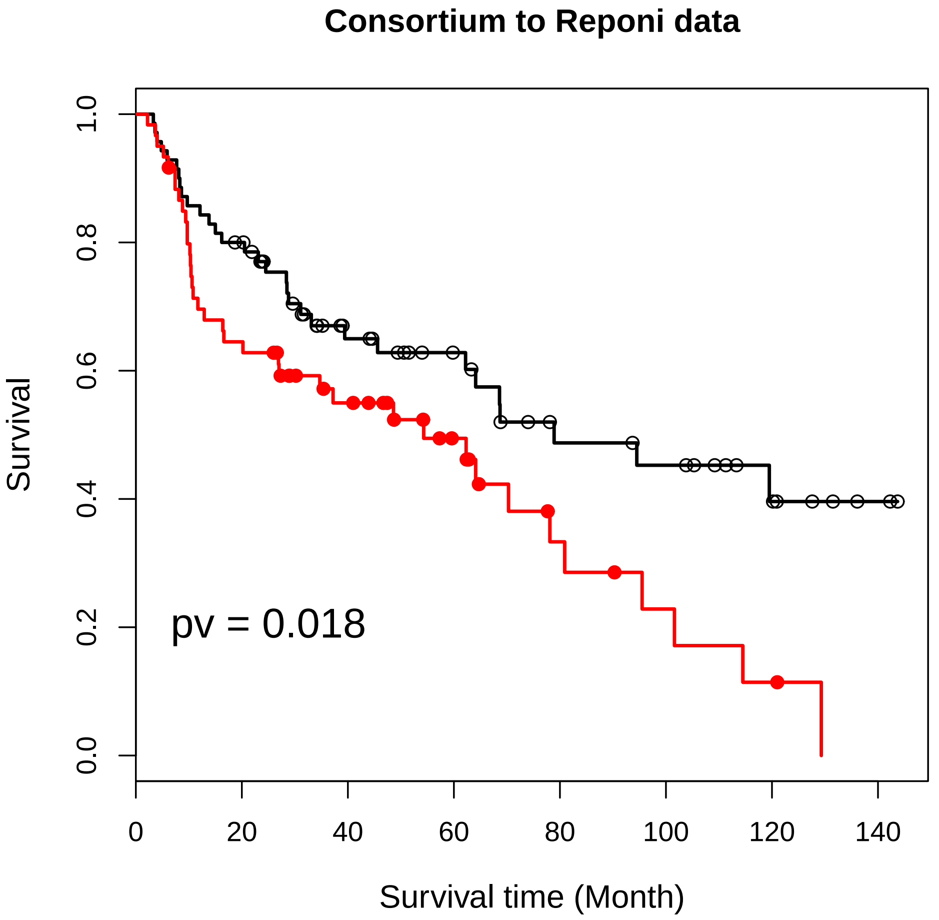

Figure S5A. Use NR gene signature developed from Consortium datasetand validated on Raponi et al dataset .

Use Raponi et al dataset to predict the survival in Consortium dataset

Read Consortium and Raponi datasets.

```
> Consortium <- read.csv("Consortium_data.csv", row.names = 1)
> Consortium.expr <- Consortium[, 10:57]
> Consortium.expr <- scale(Consortium.expr)
> Raponi <- read.csv("Raponi data.csv", row.names = 1)
```

Merge the Consortium and Raponi datasets.

```
> Raponi.expr <- Raponi[, -(1:3)]
> Raponi.expr <- scale(Raponi.expr)
> common.gene <- intersect(colnames(Raponi)[-(1:3)], colnames(Consortium.expr))
> length(common.gene)

[1] 48

> Raponi.data <- data.frame(type = "Raponi", Stage = Raponi$stage,
+     Dead = Raponi$death, Survival_Time = Raponi$month, Raponi.expr[,
+         common.gene])
> Consortium.data <- data.frame(type = "Consortium", Stage = Consortium$stage,
+     Dead = Consortium$death, Survival_Time = Consortium$month,
+     Consortium.expr[, common.gene])
> combined <- data.frame(rbind(Raponi.data, Consortium.data))

> data.train <- combined[combined$type == "Raponi", -2]
> data.test <- combined[combined$type == "Consortium", -2]
> fit <- rpart(Surv(Survival_Time, Dead) ~ ., data = data.train)
> print(fit)

n= 130

node), split, n, deviance, yval
      * denotes terminal node

 1) root 130 184.919700 1.00000000
   2) PPARd< 1.066366 109 139.431700 0.84502580
     4) CAR< -0.7709035 20  13.109090 0.25879570
       8) ERRg< -0.003237025 13   1.823978 0.08801078 *
       9) ERRg>=-0.003237025 7   5.396460 0.78524370 *
     5) CAR>=-0.7709035 89 114.511700 1.04077300
      10) NURR1< 0.4254167 65  70.532190 0.75905210
        20) CAR< 0.4008374 34  30.647770 0.45627820
          40) ERa>=0.2549306 13   1.815843 0.09207855 *
          41) ERa< 0.2549306 21  19.497330 0.77204860
            82) TRb>=-0.07715187 8   3.819955 0.33823440 *
            83) TRb< -0.07715187 13  12.157210 1.07126600 *
        21) CAR>=0.4008374 31  32.249810 1.21840700
          42) REV.ERBb>=0.8799166 7   5.689462 0.40269600 *
```

```
        43) REV.ERBb< 0.8799166 24  21.242360 1.52630600
          86) RARg< 0.141417 10   9.368298 0.94773020 *
          87) RARg>=0.141417 14   8.300546 2.14440600 *
      11) NURR1>=0.4254167 24  29.435320 2.33715800
        22) COUP.TFg< -0.3199922 11  11.589670 1.32667200 *
        23) COUP.TFg>=-0.3199922 13  11.491460 3.68556400 *
   3) PPARd>=1.066366 21  35.062900 2.12680100
     6) TR2< 0.3905393 14  19.708670 1.50652800 *
     7) TR2>=0.3905393 7  10.336000 3.70052200 *

> group <- ifelse(predict(fit, newdat = data.test) > 1, "High",
+     " Low")
> sf <- survfit(Surv(Survival_Time, Dead) ~ group, data = data.test)
> summary(coxph(Surv(Survival_Time, Dead) ~ group, data = data.test))

Call:
coxph(formula = Surv(Survival_Time, Dead) ~ group, data = data.test)

  n=440 (2 observations deleted due to missingness)

            coef exp(coef) se(coef)     z Pr(>|z|)
groupHigh 0.3547    1.4258   0.1321 2.685  0.00725 **
---
Signif. codes:  0 Ś***Š 0.001 Ś**Š 0.01 Ś*Š 0.05 Ś.Š 0.1 Ś Š 1

          exp(coef) exp(-coef) lower .95 upper .95
groupHigh     1.426     0.7014     1.101     1.847

Rsquare= 0.016   (max possible= 0.997 )
Likelihood ratio test= 7.29  on 1 df,   p=0.00695
Wald test            = 7.21  on 1 df,   p=0.007246
Score (logrank) test = 7.29  on 1 df,   p=0.006952

> logrank <- survdiff(Surv(Survival_Time, Dead) ~ group, data = data.test)
> logrank

Call:
survdiff(formula = Surv(Survival_Time, Dead) ~ group, data = data.test)

n=440, 2 observations deleted due to missingness.

             N Observed Expected (O-E)^2/E (O-E)^2/V
group= Low 203      102      123      3.45      7.28
group=High 237      134      113      3.73      7.28

 Chisq= 7.3  on 1 degrees of freedom, p= 0.00698

> pv <- pchisq(logrank$chisq, 1, lower.tail = F)
```

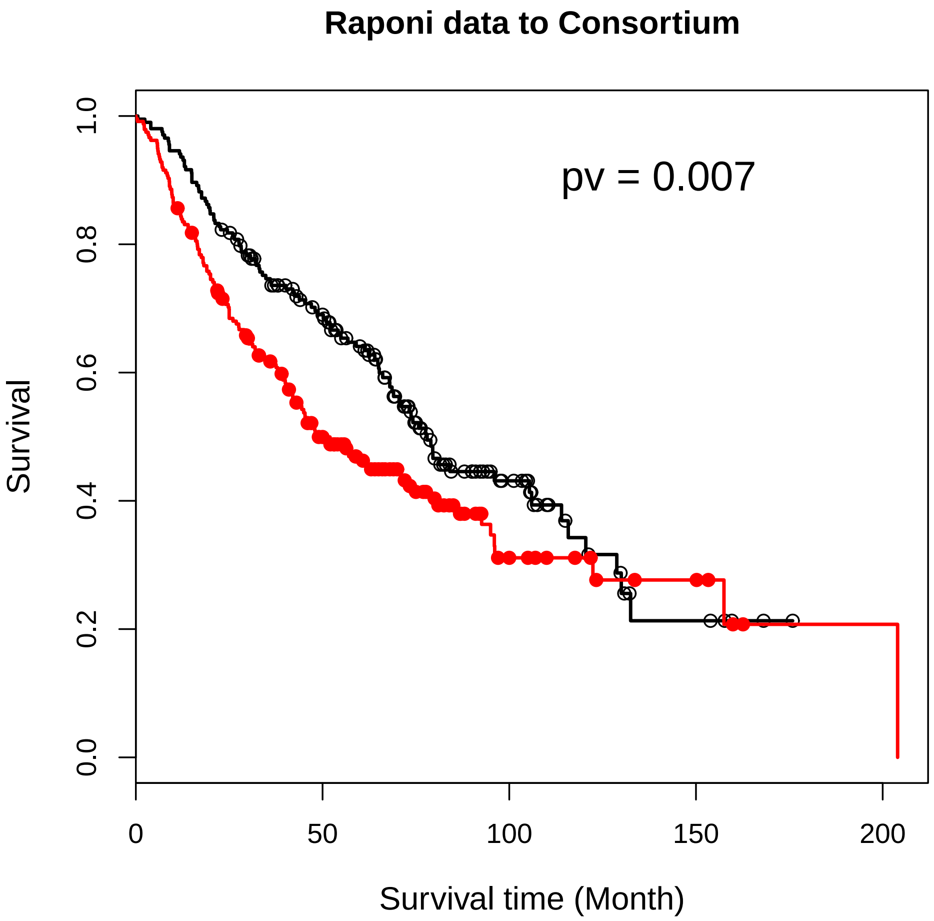

Figure S5B. Use NR gene signature developed from Raponi et al dataset and validated on Consortium dataset.

Expression correlation between tumor tissue and adjacent normal tissue

MDACC QPCR dataset

```
> dat.n <- read.csv("MDA_data_normal_Jan 24 2010.csv")
> dat.t <- read.csv("MDA_data_Jan 24 2010.csv")
> all(dat.n$SporeID == dat.t$SporeID)
```

```
[1] TRUE
```

```
> dim(dat.n)
```

```
[1] 30 55
```

```
> dim(dat.t)
```

```
[1] 30 55
```

```
> dat.cb <- data.frame(rbind(dat.t, dat.n))
```

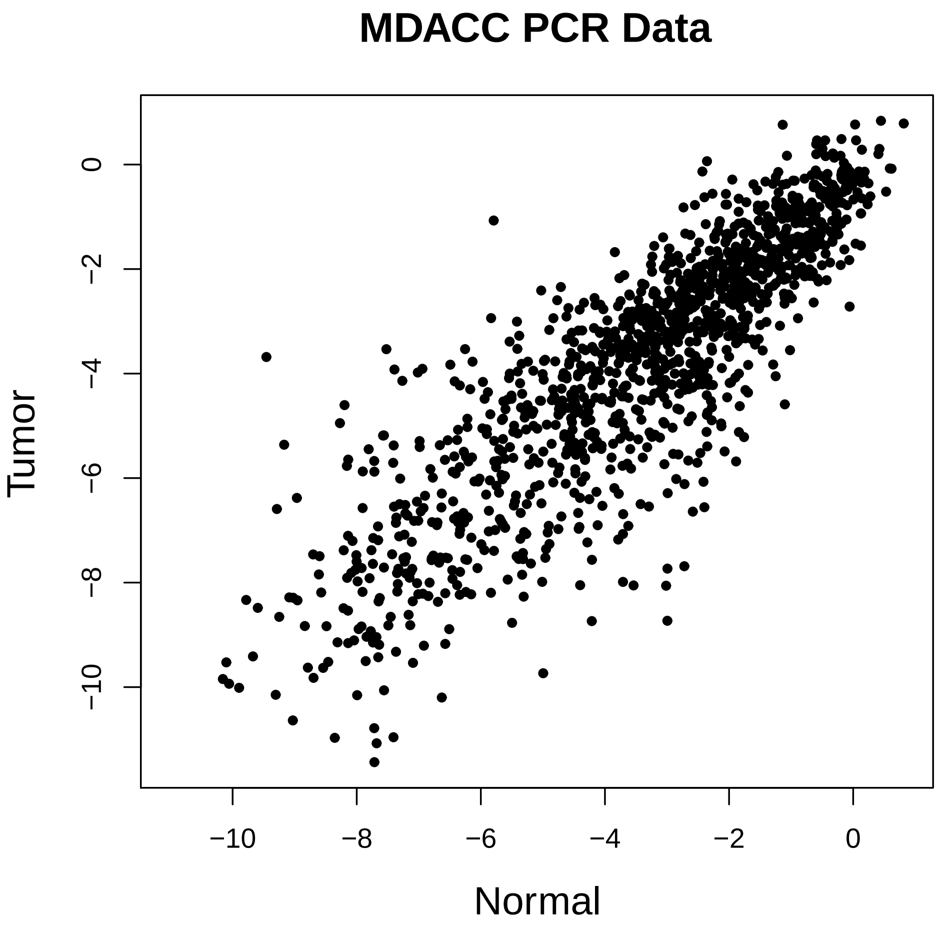

Figure S10A. Scatter plot for NR gene expression between tumor and adjacent normal samples from MDACC dataset.

Landi et al dataset

The GSE10072 (series matrix)and patient clinical information file were downloaded from GEO and saved as csv files

```
> expr <- read.csv("GSE10072_series_matrix.csv", row.names = 1)
> dim(expr)
```

```
[1] 22283   107
```

```
> expr[1:4, 1:6]

          GSM254625 GSM254626 GSM254627 GSM254628 GSM254629 GSM254630
1007_s_at 10.927084 10.416978 10.628538 10.151180 10.988512 10.778205
1053_at    6.895217  6.924856  7.550245  6.699557  6.826031  6.718372
117_at     8.110190  7.760228  7.974676  7.712676  7.775592  7.777087
121_at     9.451286  9.520943  9.807597  9.522087  9.855061  9.861055

> range(expr)

[1]  3.670126 15.248225

> id <- read.csv("NR probe ID New.csv")
> head(id)

  Probe.Set.ID mRNA.Accession Formal.Name Receptor
1  211110_s_at      NM_000044       NR3C4       AR
2    211621_at      NM_000044       NR3C4       AR
3    207007_at      NM_005122       NR1I3      CAR
4    209505_at      NM_005654       NR2F1 COUP.TFa
5  209506_s_at      NM_005654       NR2F1 COUP.TFa
6  209119_x_at      NM_021005       NR2F2 COUP.TFb

> dim(id)

[1] 110   4

> length(unique(id$Receptor))

[1] 48
```

Extract NR expression from Raponi data. If there are multiple probes corresponding to a single NR, then we take the average expression of those probes.

```
> nr <- merge(id, expr, by.x = "Probe.Set.ID", by.y = "row.names")
> dim(nr)

[1] 110 111

> expr1 <- aggregate(nr[, -(1:4)], by = list(gene = nr$Receptor),
+     mean)
> dim(expr1)

[1]  48 108

> expr2 <- t(expr1[, -1])
> colnames(expr2) <- expr1[, 1]
```

Read clinical information, merge the clinical information and NR expression.

```
> clin <- read.csv("Landi Clinical.csv")
> head(clin)
```

```
   Array.ID Pathology patient
1 GSM254625     Tumor GT00006
2 GSM254626    Normal GT00006
3 GSM254627     Tumor GT00007
4 GSM254628    Normal GT00007
5 GSM254629     Tumor GT00022
6 GSM254630     Tumor GT00042
```

```
> dim(clin)
```

```
[1] 107   3
```

```
> paired <- names(which(table(clin$patient) == 2))
> clin <- clin[clin$patient %in% paired, ]
> clin <- clin[order(clin$patient, clin$Pathology), ]
> common <- intersect(clin$Array.ID, rownames(expr2))
> length(common)
```

```
[1] 66
```

```
> out <- data.frame(clin, expr2[clin$Array.ID, ])
```

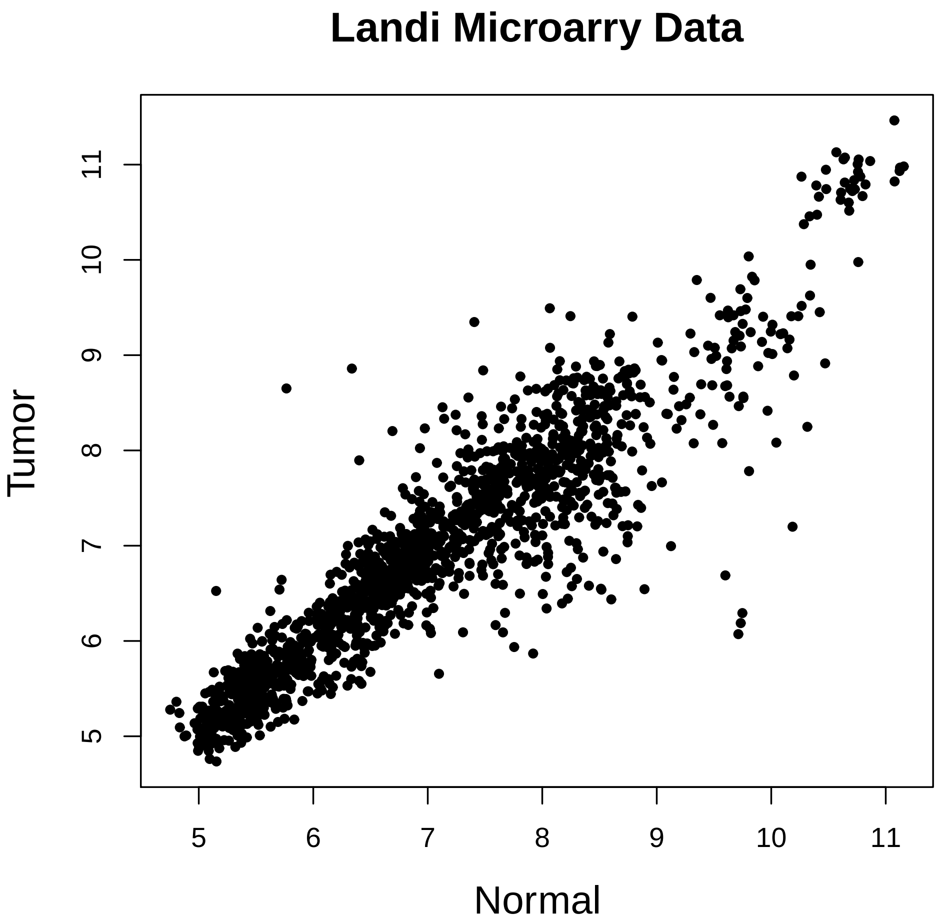

Figure S10B. Scatter plot for NR gene expression between tumor and adjacent normal samples from Landi et al dataset.

Appendix

```
> sessionInfo()

R version 2.10.0 (2009-10-26)
i386-pc-mingw32

locale:
[1] LC_COLLATE=English_United States.1252
[2] LC_CTYPE=English_United States.1252
[3] LC_MONETARY=English_United States.1252
[4] LC_NUMERIC=C
[5] LC_TIME=English_United States.1252
```

```
attached base packages:
[1] splines   stats     graphics  grDevices utils     datasets  methods
[8] base

other attached packages:
[1] survivalROC_1.0.0 rpart_3.1-45      survival_2.35-7
```
